# Synthesis of a *retro*-GFOGER Adamantane-Based Collagen Mimetic Peptide Imbibed in a Hyaluronic Acid Hydrogel for Enhanced Wound Healing

**DOI:** 10.1021/acsabm.4c01895

**Published:** 2025-02-19

**Authors:** Variksha Singh, Thashree Marimuthu, Ntlama F. Lesotho, Maya M. Makatini, Thandokuhle Ntombela, Armorel Van Eyk, Yahya E. Choonara

**Affiliations:** † Wits Advanced Drug Delivery Platform Research Unit, Department of Pharmacy and Pharmacology, School of Therapeutic Sciences, Faculty of Health Sciences, 37707 University of the Witwatersrand, 7 York Road, Parktown, Johannesburg 2193, South Africa; ‡ Molecular Sciences Institute, School of Chemistry, University of the Witwatersrand, Private Bag 3, PO WITS, Johannesburg 2050, South Africa; § Division of Pharmacology, Department of Pharmacy and Pharmacology, School of Therapeutic Sciences, Faculty of Health Sciences, University of the Witwatersrand, 7 York Road, Parktown, Johannesburg 2193, South Africa

**Keywords:** type I collagen mimetic peptide, adamantane, hyaluronic acid, hydrogel, wound healing

## Abstract

This study reported the synthesis and formulation of an adamantane-based collagen mimetic peptide (CMP) hydrogel containing the integrin-binding motif *retro*-GFOGER, designed to enable the controlled delivery of CMPs with the ability of direct wound healing for the potential treatment of acute wounds. Initially, two adamantane-functionalized CMPs (peptides NL008 and NL010) were synthesized, characterized, and comparatively screened for their in vitro biocompatibility and bioactivity. In vitro evaluations of scratch closure and biocompatibility were assessed on human-derived keratinocytes. Release and permeation of the peptides were evaluated in vitro and ex vivo. Wound closure rates and histological evaluations were performed on male Sprague-Dawley rats over 3, 7, and 14 days for the NL010-HAgel formulation. Peptide NL010 was found to be the most suitable candidate among the adamantane CMPs. For a comparative study, peptide NL010 and its palmitic acid analogue, NL009, were loaded into a hyaluronic acid (HA) hydrogel and lyophilized. The CMP hydrogels exhibited porosity (<30 μm) and were viscoelastic solids. The physicomechanical properties of the formulations showed optimal characteristics for application as wound dressings in terms of textural profile. Peptide NL008 exhibited lower bioactivity and cell viability compared to NL009 and NL010 across various concentrations and cell lines. Peptide release from NL009-HAgel and NL010-HA gel was 74% and 83%, respectively. Across an ex vivo porcine skin membrane, the CMP-HAgel showed good permeation and was retained in the epidermis and superficial dermis. CMP-HAgel at 0.1% (w/v) showed better HaCaT cell viabilities. In vitro assays demonstrated that the NL010-HA gel achieved scratch closure (99.9%) within 24 h, while the NL009-HAgel showed scratch closure (93.7%) within the same time frame. In vivo, NL010-HAgel improved healing by enhancing epithelialization and granulation tissue deposition (via fibroblast and collagen responses). The findings of this study suggested that the CMP cell-instructive hydrogel is a promising platform with the potential to accelerate wound healing.

## Graphical abstract

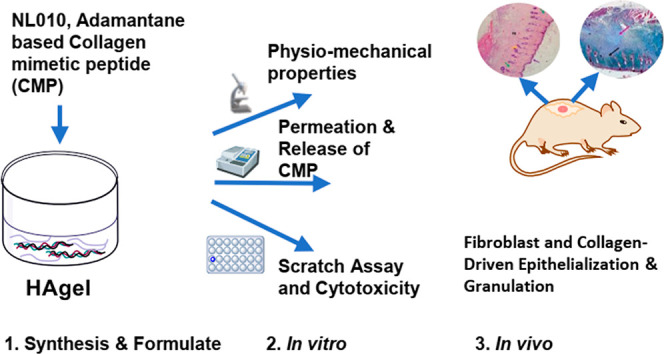

## Introduction

The skin is a complex, multilayered organ that acts as a protective barrier between the internal and external environments. However, it is prone to damage that can compromise its protective function, necessitating rapid and effective healing. Wound healing is a biological response characterized by four well-coordinated stages.^1^ Initially, clot formation results, and growth factors and cytokines are produced. Then inflammation and white blood cell infiltration follows. The proliferative phase then begins, during which newly formed cells secrete the vascular endothelial growth factor, platelet-derived growth factor, and fibroblast growth factor. In the final remodeling stage, the extracellular matrix (ECM) matures by producing collagen-rich scar tissue. Dysregulation of these processes can lead to acute wounds, which typically heal within 4–6 weeks and are characterized by soft, pink wound beds and healthy surrounding tissue, or chronic wounds, which take longer than 6 weeks to heal and may display dark red beds with excessive granulation, necrosis and signs of infection such as heat, redness, pain, and swelling.^2^

Wound management is a significant part of healthcare budgets, and effective treatment is essential to prevent poor prognoses that could lead to severe outcomes, including amputations.^3^ Common treatments include topical creams, antibiotics, growth factors, and silver sulfadiazine; however, these treatments face practical limitations. Therefore, innovative therapeutic approaches are needed to accelerate wound healing, focusing on the interactions between various cell types, biochemical cues, growth factors, and the ECM.^4^ In general, type I collagen plays a key role in skin regeneration by providing tensile strength and stability to wound sites, supporting the formation of durable scars. Wound dressings incorporating type I collagen are widely used to promote skin regeneration for chronic wounds, burns, and surgical incisions as they support tissue repair and accelerate healing. As such, they provide a solid foundation for the development of innovative therapeutic approaches for wound healing.

Tissue engineering approaches aim to replicate the healing wound’s microenvironment through various construct designs such as nanofibrous matrices, hydrogels, and 3D printed grafts. A key strategy involves creating bioplatforms that incorporate cell-instructive cues to regenerate damaged tissue. Among these, collagen mimetic peptides (CMPs) have gained attention due to their wound healing properties. CMPs can be synthesized to mimic collagen’s amino acid sequence and bioactivity, overcoming the limitations associated with natural collagen derived from animals. Moreover, CMPs can be manipulated in terms of physicochemical properties and biological signaling by modifying the amino acid sequence. Consequently, CMPs have been applied for tissue engineering^5^ and wound healing.^6^ Numerous studies have examined the design of CMPs^7^ incorporated with growth factors,^8^ integrin-targeting sequences such as GFOGER,^9,10^ and sequences like TTK (threonine-threonine-lysine) to stimulate collagen production, as reported for pentapeptide-4 (palmitoylated Lys–Thr–Thr–Lys–Ser–OH)^11,12^ or palmitoyl peptide (Pal–Lys–Thr–Thr–Lys–Ser).^13^ As such, the approach for designing the CMP is to couple hierarchical structure and biofunctionality and then investigate the effects of structure and bioactivity. A similar strategy was reported for the design of more stable peptides.^14^

CMPs are often synthesized to a (Gly-Xxx-Yyy)_*n*_ sequence pattern, and to promote thermal stability, there are characteristic glycine units at every third position. However, the size and pattern of the peptides can limit their bioactivity, and while they show promise in wound healing, they are susceptible to enzymatic breakdown, necessitating formulation or encapsulation within suitable carriers.^15,16^ Additionally, various pharmacological barriers hinder their clinical application including stability, toxicity, and effective delivery. Nonetheless, a recent comprehensive analysis of global trends in peptides and wound healing highlighted the need for chemically modified peptides for successful translation.^17^

An ideal wound dressing should exhibit excellent biocompatibility and biodegradability to minimize toxicity, maintain moisture for cellular growth, possess strong mechanical properties to prevent rupture and infection, and function as a drug delivery system to release growth factors and other bioactive substances that promote healing.^18^ To this end, hydrogels have emerged as innovative wound dressings, mimicking the natural ECM and maintaining a moist healing environment. They enhance epithelialization and cell migration while minimizing pain during dressing changes. Hyaluronic acid (HA) is a key component of the ECM that significantly contributes to wound healing and tissue repair.^19^ Moreover HA-hydrogels serve as effective wound dressings because they allow for complete filling of wound sites, in situ encapsulation of bioactive molecules, and adequate adherence to wounds.^20^

This study focuses on replicating type I collagen’s role in wound healing by designing bioactive CMPs to enhance wound healing, where synthetic peptides were designed to signal macromolecules such as growth factors and collagen that are essential for the wound healing process. The candidate CMP in question, NL010, is a collagen 1 mimetic peptide with modifications to the amino acid sequences to potentially improve the wound healing activity of the CMP (Figure 1A). It contains a DGD (aspartic acid–glycine–aspartic acid) polyanionic sequence which can attract growth factors (VEGFRs) and potentiate binding to collagen fibers through electrostatic interactions.^8^ The *retro*-GFOGER sequence is the integrin recognition sequence, which is surrounded by glycine–glycine (GG) spacer sequences on either side to reduce steric hindrance around the GFOGER sequence.^21^ The *retro*-threonine–threonine–lysine (*retro*-TTK) sequence stimulates collagen production. Adamantane was also utilized to facilitate membrane permeability since it is a fatty acid.^22^ Formulation of the novel CMPs with HA yielded CMP-HAgels (Figure 1B).

**Figure 1:**
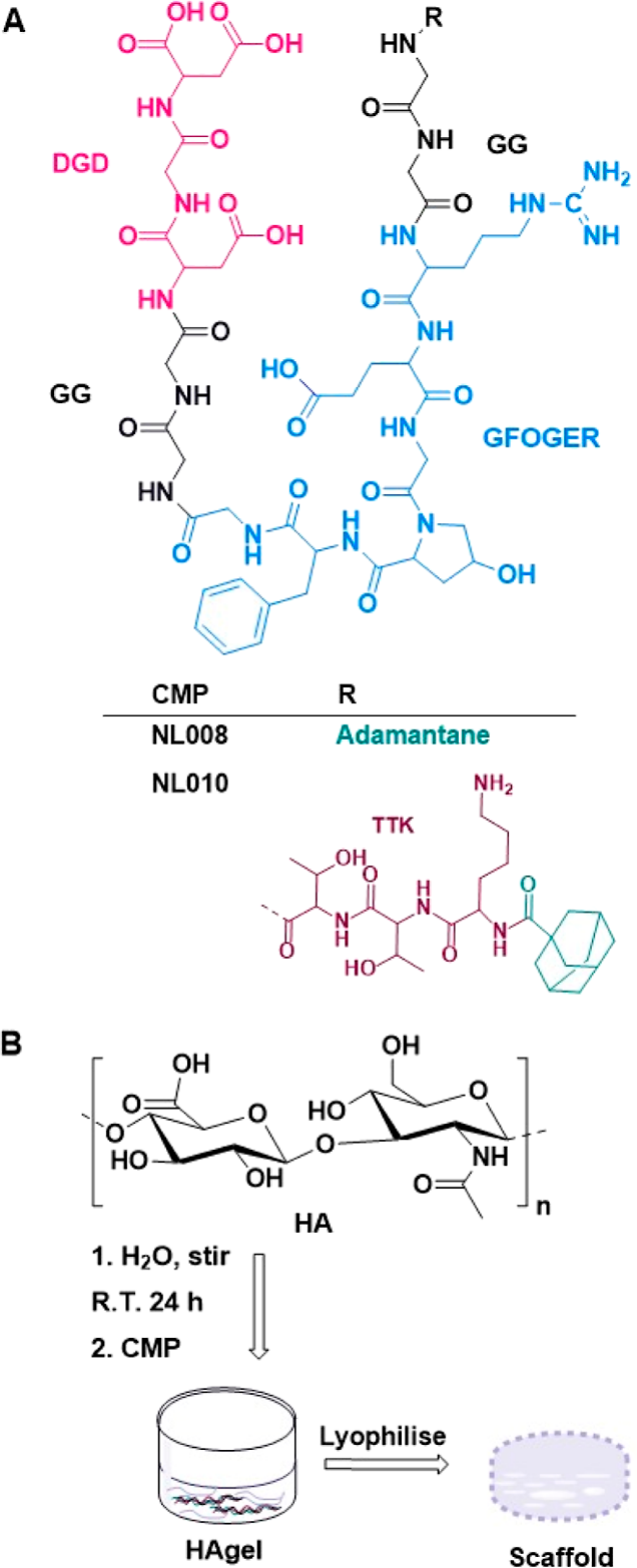
(A) Chemical structure of synthesized adamantane-based CMPs and (B) graphical illustration of the formulation of the hyaluronic acid (HA) and CMP hydrogel (HAgel) and scaffold used for characterization.

The study establishes a proof of concept for the formulation, characterization, and evaluation of HA and CMP hydrogels that could direct wound healing and improve repair outcomes for acute wounds, such as surgical, puncture, and traumatic wounds. To this end, we report for the first time the synthesis and formulation of adamantane-based CMPs containing the integrin-binding motif *retro*-GFOGER within instructive HA hydrogels. The candidate adamantane CMPs and HA hydrogels were formulated, characterized, and screened for their bioactivity and compatibility. Additionally, the sustained-release capability of the hydrogels and the healing effect of the hydrogels loaded with the newly designed CMPs on wounds caused by punch biopsy were also assessed.

## Experimental Section

### Materials

A polymer comprising of hyaluronic acid sodium salt (>95%, 1.5 × 10^6^ Da, Streptococcus equi), Fmoc-amino acids, peptide coupling agents comprising of hexafluorophosphate azabenzotriazole tetramethyl uronium (HATU), hexafluorophosphate benzotriazole tetramethyluronium (HBTU), and 2-chlorotrityl chloride resin were obtained from DLD Scientific (Durban, South Africa). Reagents and solvents for peptide synthesis and purification including piperidine, diisopropylethylamine (DIPEA), formic acid, adamantane, triisopropylsilane (TIS), dichloromethane, trifluoroacetic acid (TFA), dimethylformamide, methanol, acetonitrile, dimethyl sulfoxide, and diethyl ether were procured from Sigma-Aldrich (Germany) and Radchem and Pyramid Scientific (Gauteng, South Africa) respectively. Sigma-Aldrich (Germany) also supplied salts used to prepare simulated wound fluid like sodium chloride (NaCl), sodium hydrogen carbonate (NaHCO_3_), potassium chloride (KCl), calcium chloride (CaCl_2_), bovine albumin, Dulbecco’s modified Eagle’s medium (DMEM), Roswell Park Memorial Institute (RPMI) medium, fetal bovine serum (FBS), penicillin–streptomycin (10,000 U/mL), trypsin–EDTA solution (0.25%), phosphate-buffered saline (PBS), and thiazolyl blue tetrazolium bromide. Adhesive surgical tape was purchased from a commercial and local pharmacy (South Africa).

### Methods

#### Synthesis of Adamantane-Based Collagen Mimetic Peptides NL008 and NL010

The synthesis of NL008 and NL010 was adapted from Lesotho et al.^23^ In a typical solid-phase peptide synthesis, 600 mg of chlorotrityl resin was placed in a reaction vessel and activated overnight at room temperature with thionyl chloride (2 mL, 10 mL of anhydrous DCM). In preparation for the first coupling at the C-terminus of the resin with Fmoc-Asp­(OtBu)–OH, the resin was filtered and washed with five portions of DCM (4 mL each). A solution of 10 mL of Fmoc-Asp­(OtBu)–OH (2.40 mmol) in DCM and 2.0 mL of 1 M DIPEA was then introduced to the resin and bubbled with nitrogen for 45 min under gentle agitation. Resins were washed three times with small portions of DMF and then DCM to remove any unreacted reagents or side products. Deprotection was carried out twice in succession with a piperidine solution (20% v/v, 10 mL), under nitrogen bubbling for five min. The resin was washed three times with DMF and DCM in succession. Thereafter, the next coupling mixture of 1:0.9:5 amino acid, HBTU, and DIPEA was dissolved and added to the resin. After shaking for 45 min under nitrogen, the resin was washed as per the previous washing procedure. The coupling steps were repeated to obtain the desired sequences prior to the addition of adamantane. For this step, a coupling mixture of 1:0.9:5 adamantane (DMF 10.74 mL), HBTU, and DIPEA was added to the resin. Once reacted, the same washing procedure was carried out. The peptides were cleaved off the resin using a TFA mix (95% TFA, 2.5% H_2_O, 2.5% TIS) for 3 h after which the TFA mix was removed by filtration. The resin was washed with TFA (5 mL) and the combined filtrates were stored in 50 mL Eppendorf tubes. The crude CMP was precipitated with a cold diethyl ether. Upon centrifugation for 10 min at 5000 rpm, precipitation with cold diethyl ether was repeated three times. The CMPs were purified on a preparative high-performance liquid chromatograph, characterized using nuclear magnetic resonance (NMR) (AVANCE 300, 400 or AVANCE III 500 MHz Advance, Bruker, Germany) and circular dichroism (CD) spectroscopy (JASCO J-815 Spectropolarimeter). The CMPs were also analyzed using a Thermo Scientific Dionex UltiMate 3000 UHPLC system coupled to a Bruker Compact Qq-Time-of-Flight high-resolution mass spectrometer (Bruker Daltonics, Bremen, Germany). The detailed methods are outlined in the Supporting Information. Table 1 presents an overview of key experimental data obtained for the synthesized adamantane CMPs and the previously reported analogue. The CMP NL009 used for screening with the sequence *retro*-DGD-GG-GFOGER-GG-TTK-palmitic acid and a mass of 1760.8945 g/mol was prepared and characterized as previously reported by Lesotho et al.^23^

**Table 1:** Peptide Design and Mass Spectrometry Results for Synthesized Peptides

CMP	sequence	calculated mass (g/mol)	experimental mass (g/mol)	yield (%)	retention time (min)
NL008	*retro*-DGD-GG-GFOGER-GG-Adamantane	C_58_H_82_N_16_O_22_ 1354.5790	[M + H]^+^, 1355.5960	34	4.91
NL010	*retro*-DGD-GG-GFOGER-GG-TTK-Adamantane	C_72_H_108_N_20_O_27_ 1684.7693	[M + H]^2+^, 843.3929	30	6.01

#### Determination of CMP Stability Established via UHPLC-MS

CMPs were subjected to stress conditions to assess their stability via the verification of the amino acid composition and sequence. They were dissolved in phosphate buffer (pH 8.3) and stored at room temperature for 30 days. Weekly analyses of the CMP solutions were conducted using UHPLC as described in the Supporting Information.

#### Computational Modeling of NL008, NL009, and NL010

The linear extended peptide structures were constructed using the sequence command in the LEaP module of AMBER18.^24^ The adamantane and palmitic moieties were manually incorporated into the peptides using Discovery Studio.^25^ Subsequently, the peptide conjugates were prepared by assigning the parameters at pH 7.4 via the protein preparation workflow implemented in the Schrödinger package. The heavy atoms were energy-minimized by using the OPLS4 force field with a root-mean-square deviation (rmsd) of 0.30 Å. The system was then prepared for molecular dynamics (MD) simulations using Schrödinger’s system setup utility, where the peptides were placed in an orthorhombic box with explicit TIP3P water molecules as the solvent. To neutralize the system and balance the charge, sodium and chloride ions were added at a concentration of 0.15 M. Subsequently, the peptides were subjected to 200 ns MD simulations at 325 K (see the detailed method provided with the Supporting Information). Upon completion, the MD trajectories were analyzed to establish the peptides’ stability and flexibility using root-mean-square deviation (rmsd) and root-mean-square fluctuation (RMSF) throughout the simulation time. Also, MD snapshots of the most sampled conformations were clustered to generate a representative structure.

#### In Vitro Screening for Cytotoxicity of the CMP and CMP-HAgels

Initially, solutions of the three CMPs (NL0010, NL008, and NL009) were prepared and diluted to concentrations ranging from 100 to 6.25 μM. A humidified carbon dioxide (CO_2_) incubator set to 5% CO_2_ at 37 °C was employed for all cell culture studies. HaCaT or 3T3 cells were seeded (approximately 5 × 10^4^/well) in 96-well plates, respectively, and the cells were placed in the incubator under set conditions for 48 and 72 h, respectively. Then, each of the test concentrations for each CMP was added to the respective cells in triplicate. Cells with addition of 10 μL of 5-fluorouracil (10 μg/mL) served as a positive control (*n* = 3). The plates designated for 48 and 72 h incubation were subsequently placed in the incubator. Thereafter, 10 μL of MTT was added to the wells and incubated for 3 h. After incubation, the formed formazan crystals were dissolved in 100 μL of the solubilization solution (acidified isopropanol) and then incubated. The optical density was read at the test wavelength of 570 nm on a PerkinElmer VICTOR 2030 multilabel plate reader. The experiment was then repeated for NL009-HAgels and NL010-HAgels using concentrations ranging from 1 to 0.01% w/v. The findings obtained were used to determine the cell viability against the negative control, using the following equation.^26^ The blank represents wells with growth media only.

1 $$cell\;viability\;\left(\%\right)=\left[\frac{{OD}_{treated}-{OD}_{blank}}{{OD}_{negative\;control}}\right]\times 100$$

#### Formulation of the HA Hydrogels, CMP-Loaded Hydrogels, and Scaffolds

Hyaluronic acid (2% w/v) was dissolved in deionized water and left to gently stir at 25 rpms, on a magnetic stirrer, overnight to yield HAgels. To purify the CMPs, postcleavage exchange of trifluoroacetic acid for HCL was achieved by dissolving the peptide in 0.1 M HCl followed by lyophilization. To the HAgel solution, respective CMP NL009 and NL010, at a concentration of 0.1% w/v, were added and allowed to stand. The resulting NL009-HAgel and NL010-HAgel were thus prepared by physically mixing the CMP peptides with HAgels. The formed gel was placed in a −80 °C freezer overnight. The formulations were then lyophilized (Free Zone 12, Labcono, Kansas City, USA) for 12 h to form scaffolds.

#### Scratch Assay for Assessment of Cell Migration for CMPs and CMP-HAgels

Scratch assays are an established, cost-effective, and well-reported assay for assessing cell migration in vitro.^27^ HaCaT and NIH-3T3 cells were grown in a regular growth medium (DMEM and RPMI) supplemented with 10% FBS and 1% penicillin–streptomycin. Cells were seeded 1 × 10^5^/well in 6-well plates and incubated until confluency was attained. A vertical scratch was made using a pipet tip. Each well was then washed with 1 mL of PBS to remove any debris. Solutions of the respective CMPs were prepared at two concentrations of 12.5 and 25 μM and were used to treat the cells. An Olympus CKX53 microscope (Tokyo, Japan) was used to take images at time 0 and 12 h intervals until 72 h. The experiment was then repeated for NL010-HAgel and NL0009-HAgel at 0.1% and 0.01% w/v. Wound closure % was determined using the following equation, and the scratch area was quantified through the use of the wound healing plugin, accessible via ImageJ software (v. 1.53n).^28^

2 $$Migration\;\left(\%\right)=\left[{Area}_{at\;OHr}-{Area}_{at\;nHr}/{Area}_{at\;0Hr}\right]\times 100$$

#### Rheological Analysis of the CMP Hydrogels

Rheological analysis of NL009-HAgels and NL010-HAgels was conducted using the Haake Modular Advanced Rheometer System (II) (Thermo Fisher Scientific, Johannesburg, South Africa) with a cone and plate configuration (Rotor C35/1) at specified gap height (0.050 mm).

Prior to analysis at 37 °C, CMP HAgels were freshly prepared and kept at 10 °C overnight. Amplitude sweeps were conducted by applying a logarithmically increasing shear stress rate, ranging from 0.05 to 100 Pa while maintaining a constant frequency of 1 Hz. Frequency sweeps were then conducted using parameters obtained from the stress sweep.

#### Morphological Analysis of the CMP Scaffold

Morphological analysis was performed using field emission scanning electron microscopy (FESEM) (Zeiss Sigma, 300 VP, ZEISS Research Microscopy Solutions, Jena, Germany). Adequate resolution was obtained at acceleration voltages of 10 and 15 kV. Scaffolds were sectioned into 1 mm-by-1 mm samples, mounted onto aluminum stubs using carbon adhesive tape, sputter-coated with gold/palladium at a ratio of 2:1, and visualized at magnifications of 100, 300, and 600×. Porosity data and morphological images were analyzed using ImageJ software.

### Textural Profile Analysis of the CMP Hydrogels and Scaffolds

The bioadhesiveness, hardness, and matrix resilience of the system were evaluated using a texture analyzer (Stable Micro Systems, Vienna Court, United Kingdom) equipped with a 50 kg load cell.^29^ Deformations were applied twice in succession with an analytical probe at a depth of 15.0 mm and a speed of 2 mm/s. The times between compressions were 15 s apart. Mechanical parameters were analyzed based on the force–time curve.

### Viscoelastic Evaluation of the CMP Hydrogels

The viscoelastic properties of the NL009-HAgels and NL010-HAgels were evaluated using the ElastoSensTM Bio2 (Rheolution Instruments, Canada). Upon gelation, 3 mL of each sample was poured into the sample holder at 37 °C. The gelling and viscoelastic (*G*′ and G*) properties of the hydrogels were evaluated over 6 h at 37 °C.

### Swelling, Degradation, and Water Vapor Transmission Rate Studies

Simulated wound fluid (SWF) containing NaCl (100 mM), NaHCO_3_ (40 mM), KCl (4 mM), CaCl_2_ (2.5 mM), and bovine albumin (33 g/L) was prepared in 1 L of deionized water as described by Bradford et al.^30^ The pH was adjusted to 7.4. The NL009 scaffolds and NL010 scaffolds were placed in 5 mL of SWF. The scaffolds were weighed prior to the start of the experiment and reweighed after the removal of excess SWF every 30 min. The swelling capacity (%) was determined using the following equation

4 $$\%\;swelling\;capacity=\frac{final\;weight-initial\;weight}{initial\;weight}\times 100$$

Water vapor transmission rate (WVTR) tests were performed by placing preweighed NL009 scaffold and NL010 scaffold samples on top of a glass vial with an area of 144 mm^2^, containing 10 mL of SWF. The scaffolds were kept in an oven at 35 °C for 24 h. Scaffolds were reweighed every 2 h, and a graph of weight loss against time was constructed. The transmission rate was determined using the following equation

$$WVTR=\frac{w_{i}-w_{t}}{A}\times 10^{6}\frac{g}{m^{2}}/day$$ 3

WVTR is expressed in g^2^ h; *A*: area of the vial opening (mm^2^); *w*_i_ and *w*_*t*_: the weight of the vial before and after being placed in the oven, respectively.

### In Vitro CMP Encapsulation, Loading, and Release Assay

A standard curve was obtained from the dissolution of the peptide at varying concentrations in simulated wound fluid (pH 7.5) and phosphate buffer (pH 7.4) by using the Implen NanoPhotometer at 214 nm. The NL009-HAgels and NL010-HAgels were washed to allow for the release of surface-bound or nonencapsulated peptides. The peptide/HA ratio was indirectly calculated in order to get the expected milligrams of peptide in any given scaffold by weight. This amount of CMP was quantified by using the standard curve. From this, the encapsulation efficiency was determined. The following equations were applied to calculate the %EE and DL

5 $$EE\;\left(\%\right)=\left(M_{i}/M_{f}\right)\times 100$$

6 $$DL\;\left(\%\right)=\left(M_{i}/M_{t}\right)\times 100$$

where *M*_f_: the amount (mass) of CMP used to formulate the HAgel; *M*_i_: the actual amount (mass) of CMP found in the HAgel; and *M*_t_: the total weighed HAgel mass.

The formulated NL009 and NL010-HAgels were placed in a dialysis tube (Snakeskin 3.5 kDa, 22 mm, Thermo Fisher Scientific Inc.). The ends of the tube were sealed off, placed into 20 mL of SWF, and kept at 37 °C with stirring at 30 rpm on the orbital shaking incubator (LM-530-2, MRC Laboratory Instruments Ltd. Hahistadrut, Holon, Israel). The concentration of the respective CMP in the dissolution media was measured on Implen NanoPhotometer NP80 UV/Visible Spectrophotometer (Implen, München, Germany) at 214 nm.^31^ The cumulative release of NL0010 was calculated using the following equation.

7 cumulativerelease=[samplevolume/bathvolume]×p(t−1)+pt

where *pt*: % of CMP released at time (*t*) and *p*(*t* – 1): the % CMP released before time t.

### Ex Vivo Peptide Permeation and Deposition Studies

Porcine skin samples were collected from pigs and euthanized for other purposes from the Wits Research Animal Facility (Waiver number 18-01-2022-O). Skin samples were transported in transport fluid (PBS buffer) within 1 h of euthanasia, sectioned into 4 × 1 cm sections, placed into cryovials, snap-frozen in liquid nitrogen, and stored at −70 °C. Prior to experiments, skin samples were thawed in warmed phosphate buffer, sectioned into seven 1 × 1 cm pieces, and placed epithelial side up into 7 in-line flow-through diffusion cells (0.0397 cm^2^ area). The tissue discs were equilibrated for 10 min in PBS (pH 7.4) at 32.5 °C, after which NL009-HAgel and NL010-HAgel were applied to the donor compartments and flow cells secured with aluminum clamps. The donor compartments were covered with parafilm to prevent evaporation. PBS (pH 7.4) was pumped through the acceptor compartments at a flow rate of 1.5 mL/h. Every 2 h, 3 mL samples were collected for 24 h using a fraction collector under sink conditions, and bioactive peptide release was measured using an Implen nanophotometer. After 24 h, skin samples from the receptor chamber were pulverized and analyzed on the nanophotometer to assess CMP retention. Graphs were constructed μg/cm^2^ vs time (min) to account for the amount of peptides that permeated across the skin.

Tape stripping was employed to evaluate the skin penetration profiles of NL009-HAgels and NL010-Hagels as reported previously.^32^ Porcine skin, prepared according to the Franz diffusion assay, was blotted dry, stretched on polystyrene plates, and secured. A 2 cm^2^ area was marked for treatment and stripping, and formulations were applied as a thin layer with an 8 h residence time. Micropore adhesive surgical tape (1.5 cm width) was carefully applied to the area, ensuring consistent technique. Gentle pressure was applied using a gloved thumb to prevent contamination. After three rolling motions, the tape was removed swiftly. This process was repeated for 20 strips, with each tape (1.5 cm × 3 cm) dissolved in 1.5 mL of 96% ethanol. The samples were homogenized for 20 min, centrifuged at 15,000 rpm for 5 min, and analyzed using an Implen Nanophotometer at 214 nm. A blank tape sample was used as a control, and dilutions were performed as needed. Quantification was carried out via the pre-established calibration curve for UV–vis spectroscopy analysis.

### In Vivo Evaluation of Wound Closure Rates and Histological Analysis

Male Sprague-Dawley rats (*n* = 30) were sourced from the Wits Research Animal Facility, with approval from the University of the Witwatersrand Animal Research Ethics Committee (ethics number: 2022/02/03/C). An intramuscular injection of ketamine (40 mg/kg) and xylazine (5 mg/kg) was administered to rats under anesthesia (2% isoflurane). Two cutaneous wounds, each 6 mm in diameter and 3 mm in depth, were created on the dorsal region by using a skin punch biopsy tool. The excised skin plugs were preserved in formalin for histological analysis. Prior to treatment, images of the wounds were taken.

All treatments were sterilized and prepared under strict aseptic conditions. Microbial contamination of formulations was tested by streaking on agar plates prior to application. The study established three experimental groups with 10 rats. In each group, five rats had the left wound treated with Corning PuraMatrix peptide hydrogel (1% w/v, 0.1–0.2 mL), while the wound on the right served as a control and was covered with gauze. Similarly, in the other group of (*n* = 5), wounds on the left received treatment of hyaluronic-NL010-HAgel (0.1% w/v, 0.1–0.2 mL) and the right wound received the control of HAgel (0.1% w/v, 0.1–0.2 mL). Thereafter a gauze dressing was carefully placed over both wounds; the wounds were bandaged and held in place with adhesive tape. While the wounds only received a once-off treatment, bandages were replaced weekly. On days 3, 7, and 14 post-treatment, the wound areas were measured with digital calipers and scaled images captured. ImageJ software was used to quantitatively determine the wound areas, and the closure rates were calculated using the following equation

8 $$\%\;wound\;closure=\left(\frac{{wound\;area\;day}_{0}-{wound\;area\;day}_{n}}{{wound\;area\;day}_{0}}\right)\times 100$$

To comparatively assess the degree of wound healing, wound beds, along with the surrounding tissue, were excised at predetermined time points (days 3, 7, and 14). Histological analysis was performed on these samples to assess tissue responses, morphological changes, and pathological alterations. Following appropriate fixation in 10% buffered formalin, samples were processed according to standard procedures (IdexxSA-AP-SOP-26). To measure collagen deposition, the excised tissue was stained using Masson’s Trichrome stain.

### Statistical Analysis

All data are presented as mean ± standard deviation (SD) for *n* = 3 or with (*p*-value). Statistical analysis was conducted using OriginPro and SPSS 18.0, employing one-way ANOVA with Tukey’s test for group comparisons. A *p*-value of <0.05 was deemed to be statistically significant, denoted by *. For in vivo macroscopic analysis, results were evaluated using ImageJ software (v. 1.53n) and also presented as mean ± SD (*p*-value).

## Results and Discussion

### Design and Characterization of NL008 and NL010 by NMR and Mass Spectroscopy

The global advanced wound care market is projected to reach $18.7 billion by 2027, with significant contributions from countries like the United States, Canada, Japan, and China.^33^ It is crucial for South Africa to establish its position in the growing global market. In this study, hyaluronic acid (HA) was used, produced through fermentation by Streptococcus species to yield higher quantities at lower costs.^34^ Similar efforts to improve cost efficiency were applied to the design and synthesis of NL010. NL010 has a relatively short peptide with a simpler sequence, with solid-phase peptide synthesis (SPPS) used as a primary manufacturing method offering high efficiency.^35^ Key materials required for synthesis include protected amino acids, resins, and highly active coupling reagents to afford higher yields. Cost optimization can also be achieved through scaled production, outsourcing to specialized facilities with large-scale capabilities, and recycling reagents, ensuring proper storage conditions to minimize material losses.

While collagen is an excellent, naturally sourced material for many applications, CMPs offer distinct advantages in terms of design flexibility, specificity, and potential for innovation in wound therapy.^36^ Peptide synthesis using chemical methods offers greater consistency and scalability compared with collagen extraction from animals. The ability of CMPs to form triple helices similar to collagen enables them to bind to partially denatured collagen, which may contribute to their effectiveness as wound-healing agents.

CMPs can be synthesized with diverse structural properties, enabling the formation of stable triple helices and advancing bioengineering applications. Collagen’s hallmark structure, consisting of three polypeptide chains arranged in a triple helix, is often mimicked by peptides with repeating (Gly-Pro-Hyp)_*n*_ sequences that self-assemble into stable helices. Solid-phase synthesis is a well-established method for CMP synthesis, such as FAM-(Gly-Pro-Hyp)_7_-Gly.^37^

In this study, two structurally modified CMPs, NL008 and NL010, were designed with sequences *retro*-DGD-GG-GFOGER-GG-adamantane and *retro*-DGD-GG-GFOGER-GG-TTK-adamantane, respectively. These sequences were engineered to include epitopes that enhance cell adhesion, interact with growth factors, and promote collagen production (Table 1). The calculated and experimental masses for NL008 show excellent agreement, confirming the accuracy of the synthesis (Figure S3). For NL010, the experimental mass of 843.3929 Da [M + H]^2+^ is consistent with the calculated mass of 1684.7693 Da, taking the double charge into account, again supporting the successful synthesis of both CMPs (Figure S4). The yield for NL010 was 30% (Table 1), which is moderate for CMP synthesis.^38^ Differences in retention time (4.91 min for NL008 and 6.01 min for NL010) suggest variations in hydrophobicity, likely due to the structural modifications introduced by the *retro*-TTK sequence in NL010 (Table 1).

The structural elucidation of CMP NL008 and NL010 (Figures S1 and S2) was achieved using ^13^C NMR, DEPT 135, COSY, HSQC, and HMBC, as detailed in Tables S1 and S2 with spectra shown in (Figures S5–S7 and S10–12). All alpha protons of the spin systems identified in the TOCSY experiment (Figures S8 and S13) were detected in the ROESY spectrum (Figures S9 and S14). The ROESY spectrum reveals insights into the dipeptides present, showing two alpha protons in the alpha region: one corresponding to the alpha proton of the self-amino acid residue (i) and the other from a neighboring amino acid residue (*i* – 1). Table 2 presents the dipeptides identified for CMP entry NL0010.

**Table 2:** Determination of the Tertiary Structure of NL0010 at a Medium Coupling Range

Hyp-Arg (α-helix)	*d*_αβ_ (*i*, *i* + 3)	*d*_αN_ (*i*, *i* + 3)	*d*_NN_ (*i*, *i* + 3)	*d*_αN_ (*i*, *i* + 4)
expected interaction	4.43, 1.89	4.43, 7.69	8.32, 7.69	4.43, 8.12
observed interaction	4.43, 1.89	4.43, 7.69	8.32, 7.69	4.33, 8.12

The interactions noted for two amino acid residues that are three residues away are indicative of an α-helical structural arrangement, which aligns with the findings from the circular dichroism experiments. However, no long-range interactions were detected, suggesting that the determination of the tertiary structure indicates the potential for a helical conformation with no other distinct structures identified. Structural characterization via NMR corroborated the presence of the helical structure observed through circular dichroism analysis. Additionally, NMR studies alluded to the type of amino acids that are most likely involved in the helical conformation.

### Characterization of Triple Helix through Circular Dichroism Spectroscopy

The secondary structure of NL010 and NL009 was determined using circular dichroism analysis (Figure S15). Structurally, collagen is characterized by a right-handed bundle of three parallel, left-handed polyproline II-type helices.^39^ As such, the parallel arrangement observed for NL010 and NL009 corroborates with characteristics of natural collagen (Table 3).

**Table 3:** Characterization of the Secondary Structure of Prepared CMPs

peptide	helix (%)	antiparallel (%)	parallel (%)	turn (%)	other (%)
NL010	18.1	6.5	35.0	0	40.4
NL009	28.3	0.0	29	0	42.7

### Computational Modeling

The three-dimensional peptide structures were unveiled through molecular dynamics simulations to elucidate their structural conformations over time. The MD simulation results revealed that the NL008, NL009, and NL010 peptides adopted α-helices and β-sheet conformations (Figure S16). As per anticipated conformations of the peptides in Figure 2, NL008 adopted the β-sheet, while NL009 and NL010 adopted mixed α-helix and β-sheet conformations, which were predominated by α-helix conformations as confirmed by the Ramachandran plots (Figure S17). The Ramachandran plots provide crucial information concerning the statistical distribution of the psi (Ψ) and phi (Φ) dihedral angles, often used to verify the determined structural conformations. Likewise, the peptides’ activities are often associated with their conformations, mostly stabilized by strong intermolecular hydrogen bond interactions observed in all the peptides. Furthermore, the peptides’ stability and flexibility were measured using the root-mean-square deviations (rmsd) and root-mean-square fluctuation (RMSF) of the C-α backbone throughout the simulation and revealed conformational transitions and variations, likely influenced by the incorporated moieties. Hence, the insight from computational modeling concurs with CD measurement findings regarding the peptides’ conformations.

**Figure 2:**
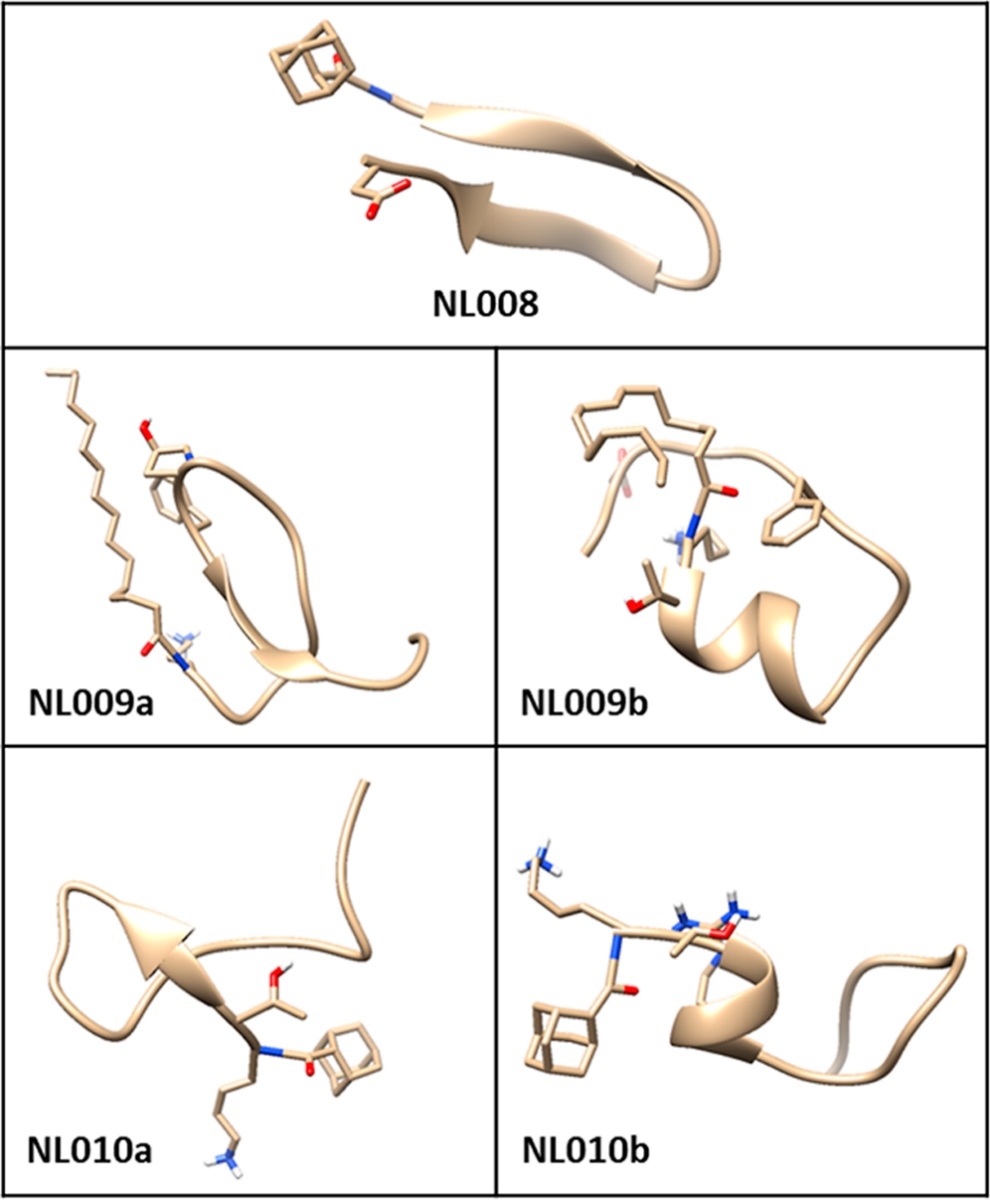
Three-dimensional structural conformations of the investigated CMPs showing their folded states obtained through MD simulations. CMP stability established via HPLC-MS.

Peptide stability is an important consideration in formulation development. Physical stability of the peptide relates to biological activity, toxicity, and immunogenicity.^40^ As a result, we investigated the stability of the CMPs to ensure their safety and efficacy. The CMPs were found to be stable in solution for a 14 day period (Figure S4.2), and thereafter, peaks uncharacteristic of the CMPs were noted, indicating some CMP fragments.

### In Vitro Cytotoxicity and Biocompatibility of CMPS on HaCaT Keratinocytes and 3T3 Fibroblasts

Preliminary screening of CMP bioactivity was established on human-derived keratinocyte HaCaT and murine-derived NIH 3T3 fibroblasts. Keratinocytes and fibroblasts account for the main sources of in vitro wound healing work owing to their central role in the physiological process. The HaCaT and NIH-3T3 cell lines were selected to evaluate the wound healing efficacy and safety of the test formulations in a wound-like environment due to their critical roles in the wound healing process.^41^ HaCaTs, as keratinocytes, are essential for epidermal regeneration, with their proliferation and migration driving re-epithelialization, making them widely used in wound healing studies.^42^ NIH-3T3 fibroblasts, on the other hand, are crucial for maintaining structural integrity by secreting ECM components like collagen and fibronectin, with their recruitment and migration being key to wound healing.^43^

All CMPs maintained or enhanced cell viability at lower concentrations, demonstrating a dose-dependent relationship (Figure 3A,B). Higher concentrations (100 μM) generally resulted in reduced viability and potential cytotoxic effects, indicating that optimal peptide dosages should remain relatively low. As such, the optimal concentrations for the CMPs at 12.5 and 25 μM were carried forward for further evaluation in scratch assays to assess their role in promoting cell migration and proliferation. These findings illustrated the potential of these peptides to enhance cell viability while highlighting the importance of concentration to maintain biocompatibility.

**Figure 3:**
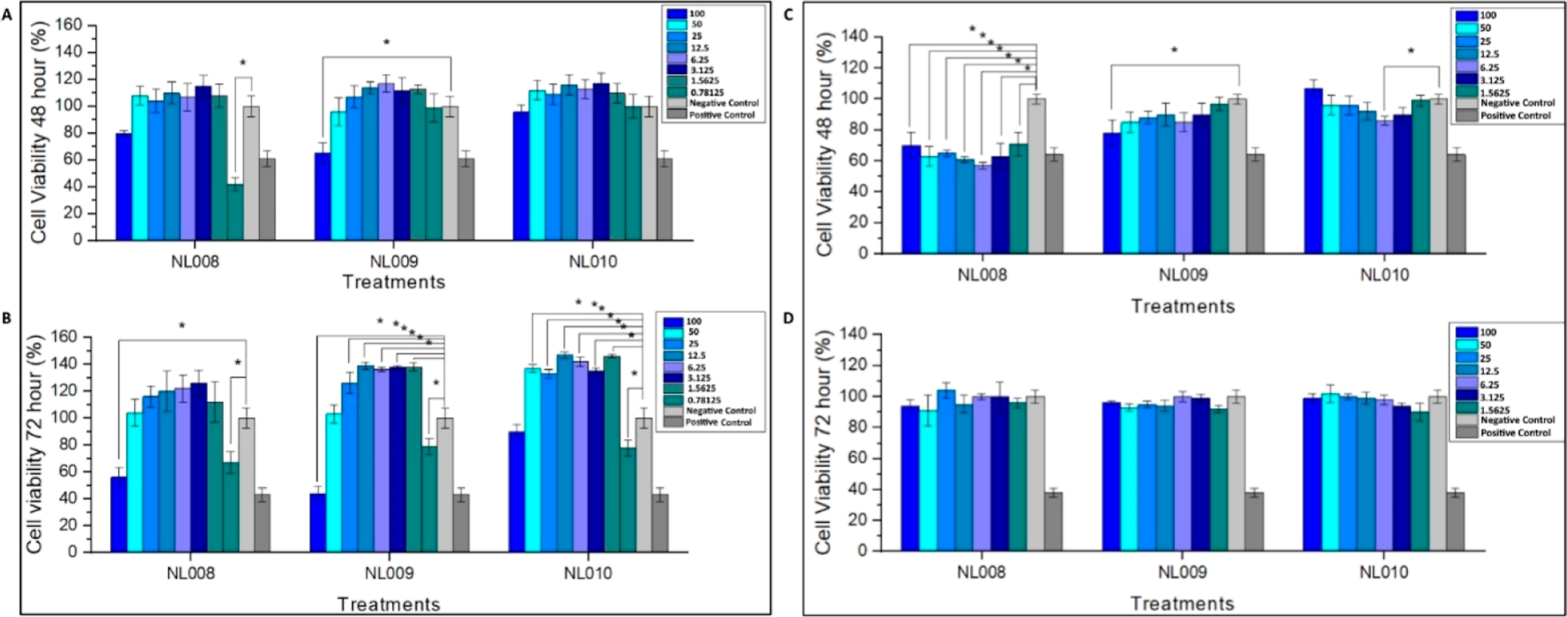
Cytotoxicity assays to assess cell viability for 5 concentrations ranging from 100 to 0.7813 μM over 48 (a,c) and 72 h (b,d) on HaCaT cells (a,b) and NIH 3T3 (c,d) cells (*n* = 3).

Unlike the HaCaT cell line, the NIH-3T3 cell line exhibited an inverted bell-shaped trend in cytotoxicity with higher concentrations leading to increased viability followed by a central dip and then an increase at the tail end (Figure 3C,D). Overall, cell viability was slightly lower than (48 h) or similar to (72 h) that of the negative control. Based on the cytotoxicity results, optimal concentrations of 50 and 25 μM were selected for further investigation in scratch assays to assess the CMPs’ roles in promoting cell migration and proliferation. The evaluation of CMPs on the NIH-3T3 cell line revealed that, while some improvement in cell viability was noted after 72 h, the overall viability remained lower compared to the HaCaT cell line. The findings suggest a complex relationship between CMP concentration and cytotoxic effects, warranting further exploration into their potential roles in cell migration and proliferation.

### Peptide Bioactivity Assessed on HaCaT Keratinocytes and 3T3 Fibroblasts

The study assessed the migration effects of peptides NL008, NL009, and NL010 on cell scratch closure rates over 24, 48, and 72 h at varying concentrations (Figure 4). Software analysis, computed via ImageJ, outputted a % migration graph, whereby all peptides of varying concentrations were evaluated simultaneously (see Figure S18 Microscopic scratch closures). At the 24 h mark, peptide NL009 at 12.5 μM (43.2% ± 5.6) and 25 μM (48.4% ± 3.3) showed better scratch closure compared to NL010 at corresponding concentrations. NL009 at 25 μM demonstrated statistically significant improvement compared to the control (*p* = 0.02). By 48 h, NL008 showed moderate closure rates similar to the control, while NL009 at 12.5 μM outperformed NL010, achieving 81.6% ± 2.1 closure compared to NL010s at 71.1% ± 3.2 (*p* = 0.022). However, NL010 at 25 μM achieved better closure (82.3% ± 3.3) than NL009 at 25 μM (79.3% ± 5.2), though the difference was not significant (*p* = 0.92). Both NL009 and NL010 displayed significant improvements over the control (*p* < 0.05). At 72 h, NL010 at 25 μM achieved nearly complete scratch closure (99.9% ± 4.1), surpassing NL009 (97.1% ± 2.8), though this was not statistically significant (*p* = 0.95). NL008 remained less effective, with a minimal difference from the control. Overall, NL010 at 25 μM showed superior scratch closure, particularly within the first 48 h, indicating heightened bioactivity early on.^44^ NL008 was the least effective peptide, while NL009 performed well but did not surpass NL010 at higher concentrations (Figure 4A). Based on this analysis and cytotoxicity, NL010 at 25 μM emerged as the most effective peptide, demonstrating strong cell-migration-promoting effects that significantly enhanced scratch closure in vitro.

**Figure 4:**
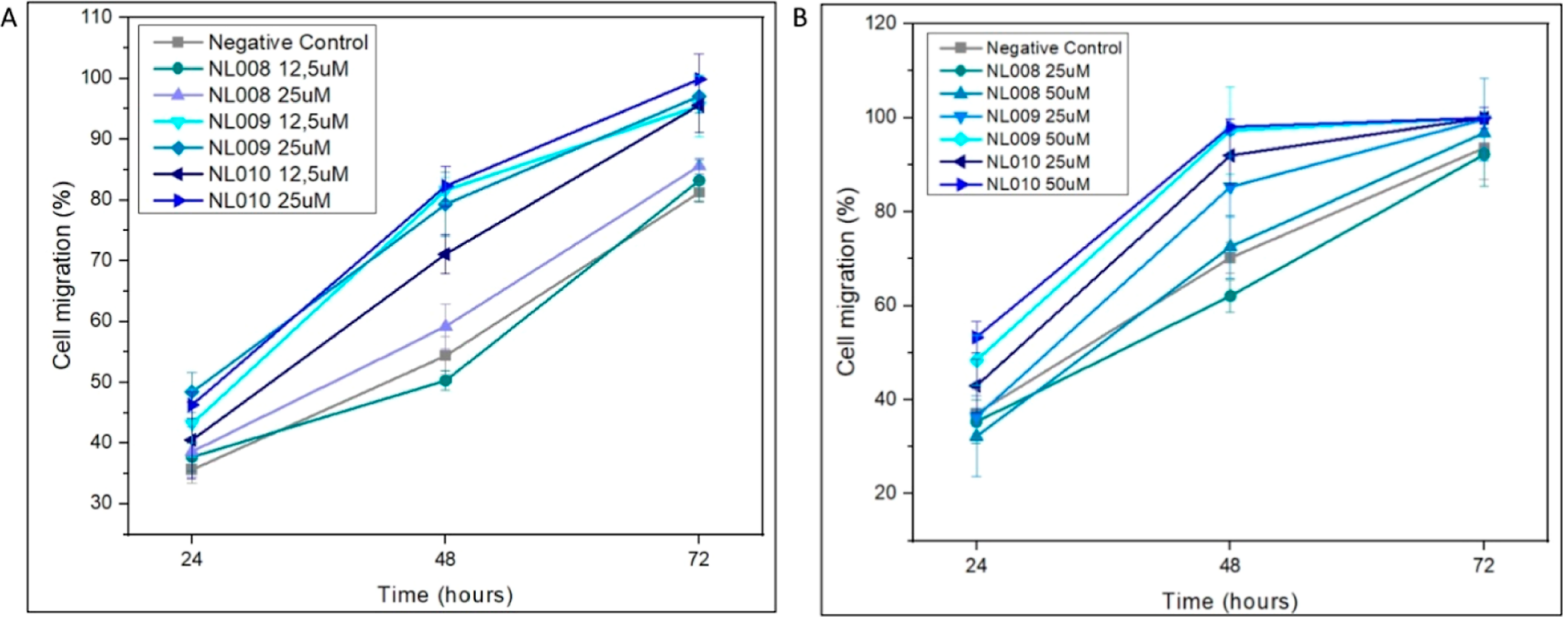
Scratch closure rates NL008, NL009, and NL010 on the (a) HaCaT and (b) NIH 3T3 cell lines expressed as % migration (*n* = 3).

The study evaluated the effects of the three CMPs on scratch closure rates in NIH 3T3 cells (Figure 4B). At 24 h, only NL010 at 50 μM showed statistically significant improvement compared to the negative control (*p* = 0.028). By 48 h, NL009 at 50 μM (97.3% ± 9.4) and NL010 at 50 μM (98.1% ± 1.6) showed comparable results, with both peptides demonstrating statistical significance against the negative control (*p* < 0.05). At 72 h, the scratch closure rates increased progressively from 24 to 48 h, followed by a tapering effect from 48 to 72 h, suggesting peptide degradation and an initial burst of cell proliferation. NL010 at 50 μM was the most effective peptide, showing superior cell-migratory effects and promoting enhanced scratch closure in vitro. Both NL009 and NL010 exhibited faster scratch closure on NIH 3T3 cells compared to HaCaT cells, highlighting their efficacy in promoting cell migration and healing (refer to Figure S19. Microscopic scratch closures).

These screening results showed that CMPs NL009 and NL010 induce significant cell migration and proliferation (Figure 4A,B), while NL008 showed minimal bioactivity and was excluded from further studies due to its poor performance and high formulation costs (Figure 4B). Scratch closure rates peaked within 24–48 h, followed by decreased activity, likely due to peptide degradation.

Peptides with α-helical structures are often more stable and interact more effectively with membrane-bound proteins,^45^ which can play a crucial role in regulating collagen stability and structure during the wound-healing process.^46^ For example, NL008 adopts a β-sheet conformation, while NL009 and NL010 exhibit mixed α-helix and β-sheet conformations. α-Helical structures predominate in NL009 and NL010, rendering NL008 the least active. The shorter peptide NL010, modified with adamantane, showed relatively higher diffusion rates compared to NL009, likely leading to improved cellular uptake and keratinocyte proliferation.^47^ In NL0010, the cage-like and hydrophobic nature of adamantane enhances its interaction with cell membranes without interacting with the peptide sequence. Furthermore, the unique cage-like structure of adamantane adds hydrophobicity, potentially enhancing cellular uptake.^48^ However, in peptide NL009, the flexible chain-like structure of palmitic acid may interact with the peptide sequence hindering optimal binding with integrin. Future computational work should explore CMP integrin binding models to further substantiate these findings.

### CMP Hydrogels and Scaffolds

Through the defined method, a transparent clear hydrogel was formed, as seen in Figure 5A­(i,ii). Biopolymer-based hydrogels are three-dimensional matrices capable of absorbing large amounts of biological fluids such as wound exudate. The porous structure of hydrogels, as seen by the SEM imaging, fashion an ideal microenvironment to hold cells in place and transport nutrients, wastes, and other fundamental cellular cues.^49^ Upon lyophilization by the defined method, a spongy white three-dimensional hydrogel-based scaffold was formed as seen in Figure 5A­(iii,iv).

**Figure 5:**
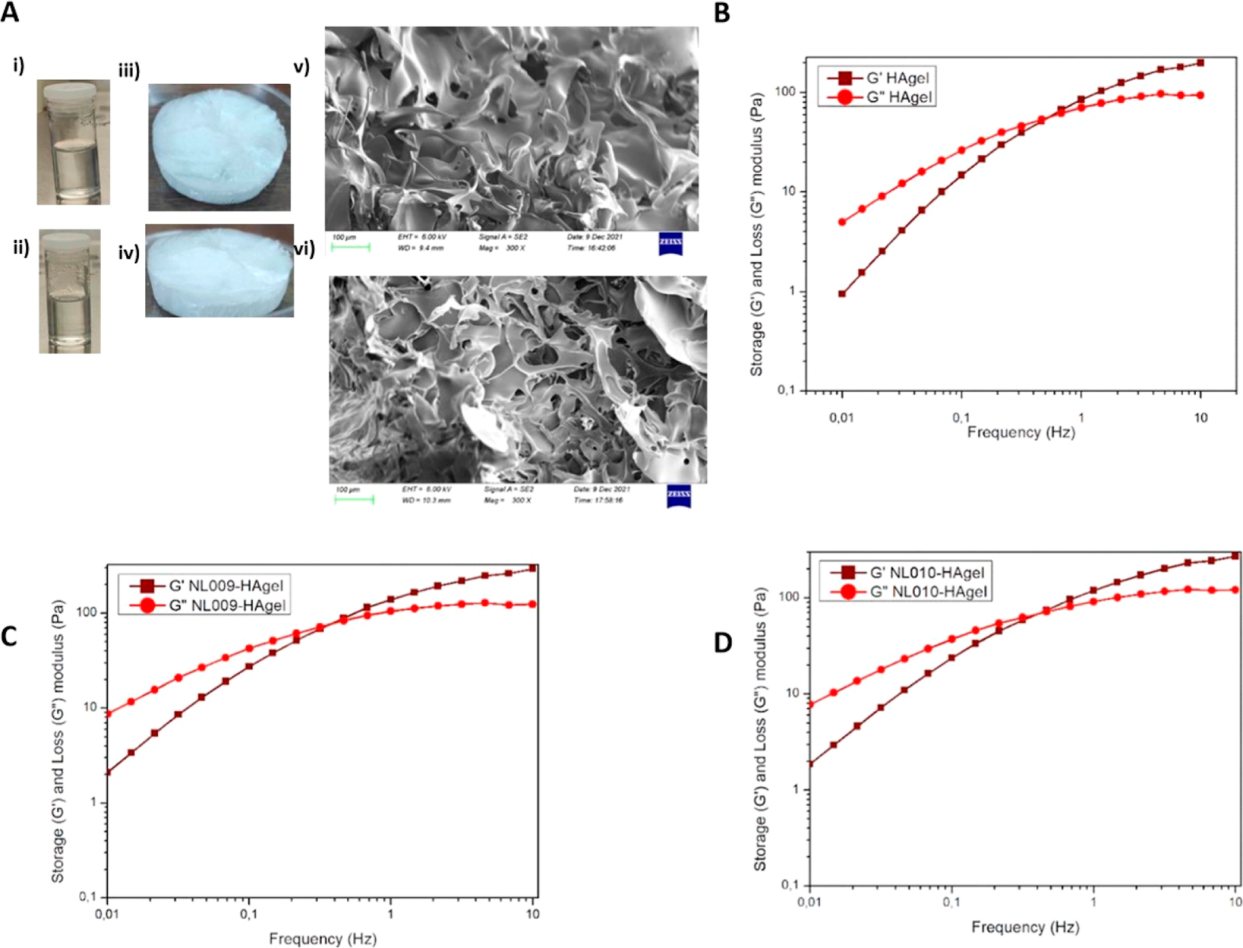
(A) From left to right: NL009 and NL010 HAgel (i,ii), scaffold (iii,iv), and SEM images of scaffolds (v,vi) respectively. Frequency sweep (Pa) as a function of the storage and loss moduli of (B) HAgel, (C) NL009-HAgel, and (D) NL010-HAgel.

### Rheological Analysis

The rheological properties of HAgel, NL009-HAgel, and NL010-HAgel across a frequency range of 0–10 Hz were observed. Both moduli increased with frequency before plateauing, with *G*′ surpassing *G*″ at the crossover point, indicating the gels’ viscoelastic solid nature due to interactions between CMPs and HA. HAgel exhibited the lowest *G*′ (198 Pa) and *G*″ (93 Pa) (Figure 5B), followed by NL009-HAgel (*G*′ 291 Pa, *G*″ 124 Pa) (Figure 5C) and NL010-HAgel (*G*′ 271 Pa, *G*″ 120 Pa) (Figure 5D). The addition of CMPs improved gel strength, with NL009-HAgel and NL010-HAgel reaching the *G*′ > *G*″ crossover at lower frequencies than HAgel, showing potentially stronger CMP–HA interactions.

An appropriate pore size for wound dressing materials ranges from 20 to 120 μm.^50^ Within this range, the wound dressing is able to regulate the migration speed of cells and allows for optimal biological conditions such as optimal cellular function and viability, maximizing wound repair mechanisms. While minor differences were noted in the scaffolds, both displayed resounding similarities and a porous, microlayered nature. The freeze-drying process effectively generated and controlled porosity in hydrogel-based scaffolds, as observed in SEM images of NL010 and NL009 scaffolds (Figure 5A­(v,vi)). Rapid cooling and sublimation created porous voids, with pores largely maintained under 30 μm due to the lyophilization technique. Both scaffolds exhibited a rough, filamentous morphology with microlayered structures and microspaces that contribute to structural support, wound exudate absorption, and cellular anchorage. Pore sizes ranged from 14.73 to 24.01 μm for NL010 and 4.517 to 20.3 μm for NL009, with minor differences in surface roughness and fiber dimensions. These features align with the requirements for wound dressing materials, ensuring optimal cell migration and biological functionality for enhanced wound healing.

### Textural Profile of the CMP HAgels and Scaffolds

Key mechanical properties, including hardness, compressibility, adhesiveness, cohesiveness, and elasticity, were evaluated using textural profile analysis.^51–53^ Due to the high water content and gelling properties of hyaluronic acid hydrogels, these formulations offer reasonable mechanical characteristics.^51^ The gel parameters of the dressings were evaluated using the force–time plot and are presented in the Supporting Information (Figure S20). Results are listed in Table 4. CMP hydrogels showed greater hardness than the HAgel control, with NL009-HAgel being harder than NL010-HAgel. CMP hydrogels also demonstrated superior compressibility, adhesiveness, and elasticity^54^ compared to the control, with NL009-HAgel outperforming NL010-HAgel. CMP-HAgels exhibited stronger cohesiveness, suggesting improved structural integrity and resistance to shear stress. Overall, CMPs enhanced the gel strength by forming physical interactions with hyaluronic acid.

**Table 4:** Mechanical Properties of the Hydrogels

formulation	hardness (Newtonian force ± SD)	compressibility (Newtonian force ± SD)	adhesiveness (Newtonian force ± SD)	minimum retracting force (Newtonian force ± SD)	cohesiveness (Newtonian force ± SD)	elasticity (sec ± SD)
HA	0.1098 ± 0.02	0.419 ± 0.03	0.42 ± 0.003	0.04 ± 0.064	1.022 ± 0.03	1.14 ± 0.032
NL009-HAgel	0.1304 ± 0.001	0.602 ± 0.001	0.558 ± 0.0001	0.06 ± 0.036	1.0545 ± 0.021	0.875 ± 0.057
NL010-HAgel	0.1205 ± 0.0006	0.455 ± 0.008	0.441 ± 0.009	0.05 ± 0.086	1.065 ± 0.046	0.88 ± 0.041

The viscoelastic properties of hydrogels were assessed, showing both elastic and viscous behaviors. Elastic materials regain shape after stress, while viscous materials deform permanently. All hydrogels exhibited *G*′ > *G*″, indicating gel formation, with CMP hydrogels displaying higher *G*′ values than HAgels (Figure 6): 1500 Pa for NL009-HAgel (Figure 6B) and 1200 Pa for NL010-HAgel (Figure 6C), compared to 1000 Pa for HAgel (Figure 6A). This suggests stronger mechanical strength due to possible CMP interactions. NL009-HAgel showed the highest elasticity, which was attributed to its crystalline structure. Tan (δ), representing the ratio of viscosity to elasticity, was lower in the CMP hydrogels, indicating more solid structures. NL009-HAgel had the lowest tan (δ), reflecting a structured gel network, while NL010-HAgel exhibited a similar but slightly lesser performance (Figure 6D).

**Figure 6:**
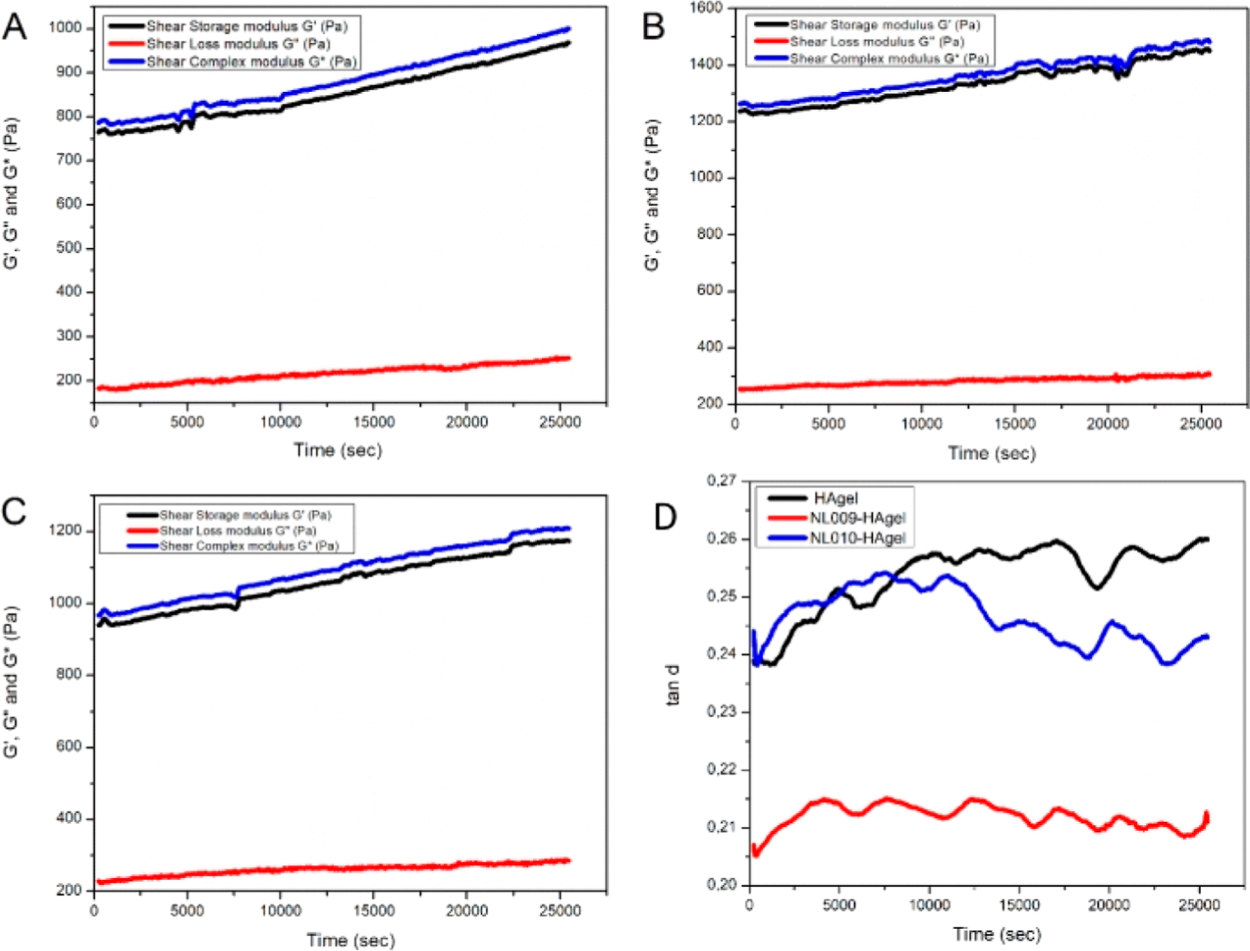
Viscoelastic properties (*G*′, *G*″, and G* Pa) of (A) HAgel; (B) NL009-HAgel; (C) NL010-HAgel; and (D) the loss moduli of the three hydrogels.

Swelling and degradation of biomaterials were assessed. The HAgel scaffold swelled quickly, reaching 200%, but began degrading into a liquid after 25–30 min (Figure 7A,B). CMP HAgels swelled more slowly but reached higher swelling levels of 400% for NL009 and 397% for NL010. NL009 swelled more gradually, indicating a more structured form. Degradation profiles showed an initial lag phase, with the HA scaffold degrading to 10% and CMP scaffolds degrading to 20%, highlighting the CMPs’ greater integrity. Rapid degradation within 20 min suggests quick biodegradation and potential for bioactive release in response to the wound exudate. Swelling behavior strongly correlates with SEM-determined porosity as both provide complementary insights into the scaffold’s structural and functional properties. SEM images reveal the presence of micropores and interconnected voids, which directly influence the material’s ability to absorb and retain fluids. Larger or more numerous pores, as visualized in the SEM micrographs, facilitate greater swelling by allowing for increased fluid infiltration into the porous matrix. For instance, the microlayered and filamentous structures observed in the NL010 and NL009 scaffolds create channels and voids that enhance permeability, supporting fluid absorption. The swelling behavior therefore reflects the scaffold’s porosity and materials with higher porosity exhibit greater swelling ratios (Figure 7A,B). This alignment confirms that the porous microstructure contributes to the scaffold’s ability to absorb wound exudates effectively, reinforcing its suitability for applications requiring high fluid retention such as wound healing. The observed swelling behavior aligns with the structural observations from SEM, validating the relationship between porosity and functional performance.

**Figure 7:**
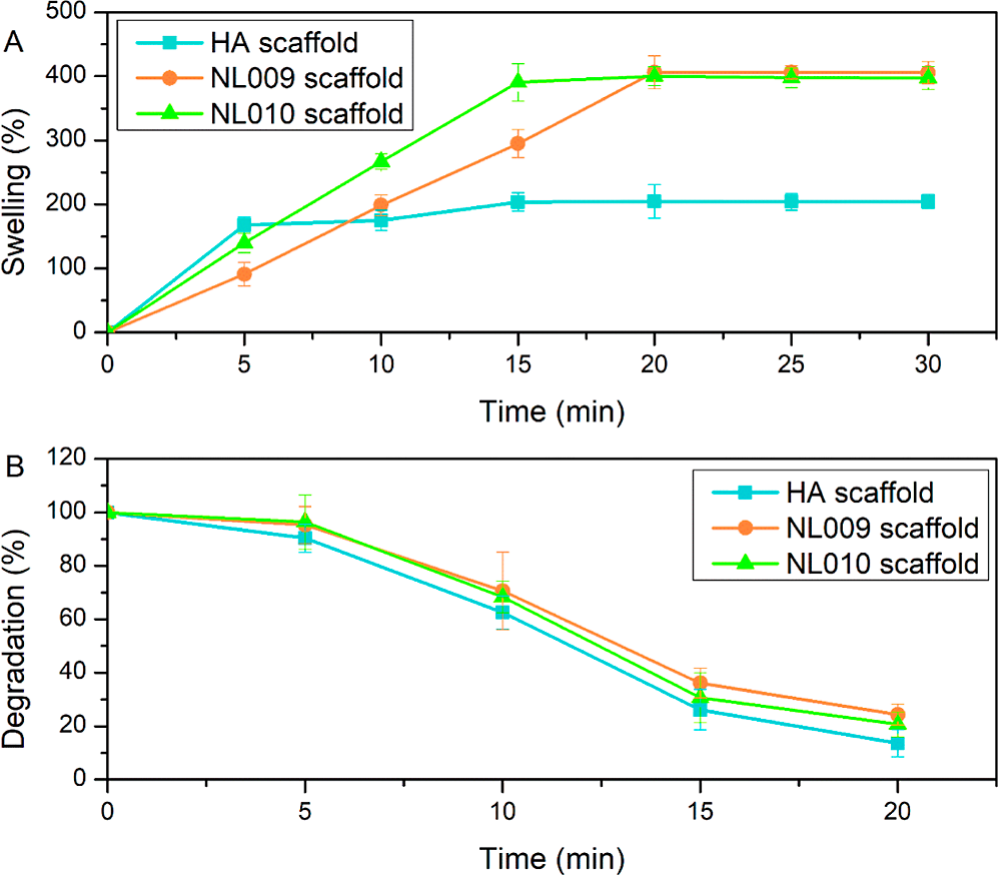
Swelling (A) and degradation (B) profiles of the HA, NL009, and NL010 scaffolds.

Water vapor transmission rate (WVTR) is crucial in biomaterials for wound healing as it regulates gas and nutrient exchange. A moist wound environment accelerates healing,^55^ but excessive WVTR can dehydrate the wound, while too low a rate risks bacterial contamination. Normal skin has a WVTR of 204 g/m^2^/day, with optimal wound healing requiring around 2500 g/m^2^/day.^56,57^ In this study, HA scaffolds exhibited a WVTR of 184 g/m^2^/day, while NL009 and NL010 scaffolds had rates of 163 g/m^2^ and 165 g/m^2^/day, respectively—below the recommended rate.^58^ These values are influenced by scaffold properties such as thickness, porosity, water uptake, and degradation, highlighting the need to balance the WVTR for effective wound biomaterial design.

### Encapsulation, Drug Loading, and In Vitro Bioactive Peptide Release Studies

The encapsulation efficiency for NL009 and NL010 hydrogels was 82 ± 3.2% and 86 ± 6.8%, respectively, while their drug load capacity was 12 ± 2.6% and 11 ± 1.6%. In wound care, frequent dressing changes can be tedious and costly, making controlled drug release with fewer changes the ideal solution. This study aimed to develop a hyaluronic-acid-based system capable of controlled bioactive release. In vitro studies revealed typical diffusion-controlled release, following Fick’s diffusion theory,^59^ with an initial burst followed by a slow release (Figure 8). NL009-HAgel, with a more structured network, showed 74% cumulative release, while NL010-HAgel exhibited 83%, with a more rapid burst release.

**Figure 8:**
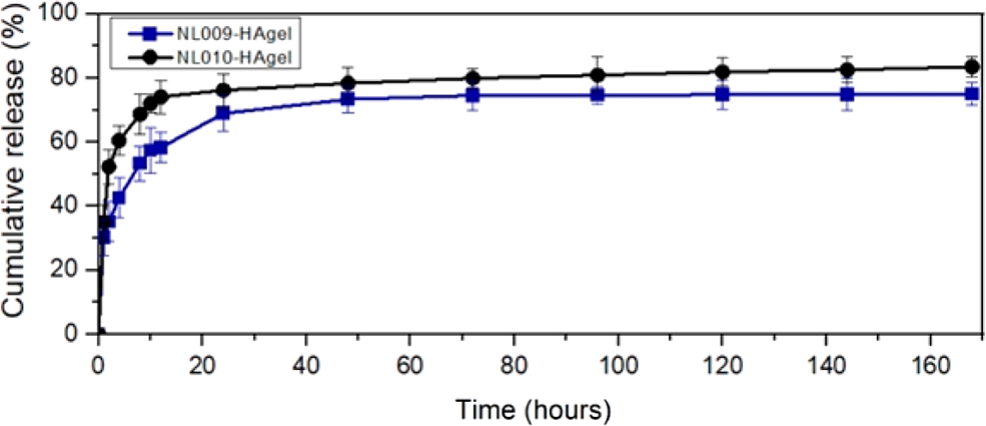
In vitro cumulative (%) bioactive release of NL009 and NL010-HAgels.

### Ex Vivo Peptide Permeation and Deposition Studies

To evaluate the ex vivo efficacy of the wound healing system, the local bioavailability of the CMPs was assessed by using excised porcine skin. Measuring peptide concentration in skin layers after topical application poses challenges due to the large molecular weights of the CMPs (1761.93 Da for NL009 and 1685.75 Da for NL010).^60^ To enhance permeation, NL009 and NL010 were modified with palmitic acid and adamantane moieties, respectively. Adamantane has been well established as a lipid solubilizing group in the development of drug candidates.^61^ Furthermore, adamantane has recently been reviewed as an emerging tool for the formulation of adamantane-based nanoparticles and polymers to improve the pharmacokinetic profiles of bioactives.^62^ Palmitic acid is known to disrupt stratum corneum lipids, facilitating diffusion.^63^ Figure 9 illustrates the peptide permeation profiles. NL009-HAgel displayed a general profile with a slight plateau between 22 and 24 h, achieving a maximum cumulative amount of 158 μg/cm^2^ (Figure 9A). In contrast, NL010-HAgel demonstrated superior permeation, reaching a maximum cumulative amount of 228 μg/cm^2^, indicating the greater efficacy of the adamantane moiety for skin penetration (Figure 9A). The relatively smaller molecular weight of NL010 (76.18 Da less than NL009) further contributed to its enhanced permeation. Both hydrogels provide hydration to the skin, improving permeation while remaining localized in the epidermis and superficial dermis (Figure 9B), which is ideal for targeted topical drug delivery and helps avoid systemic absorption and associated toxicity.

**Figure 9:**
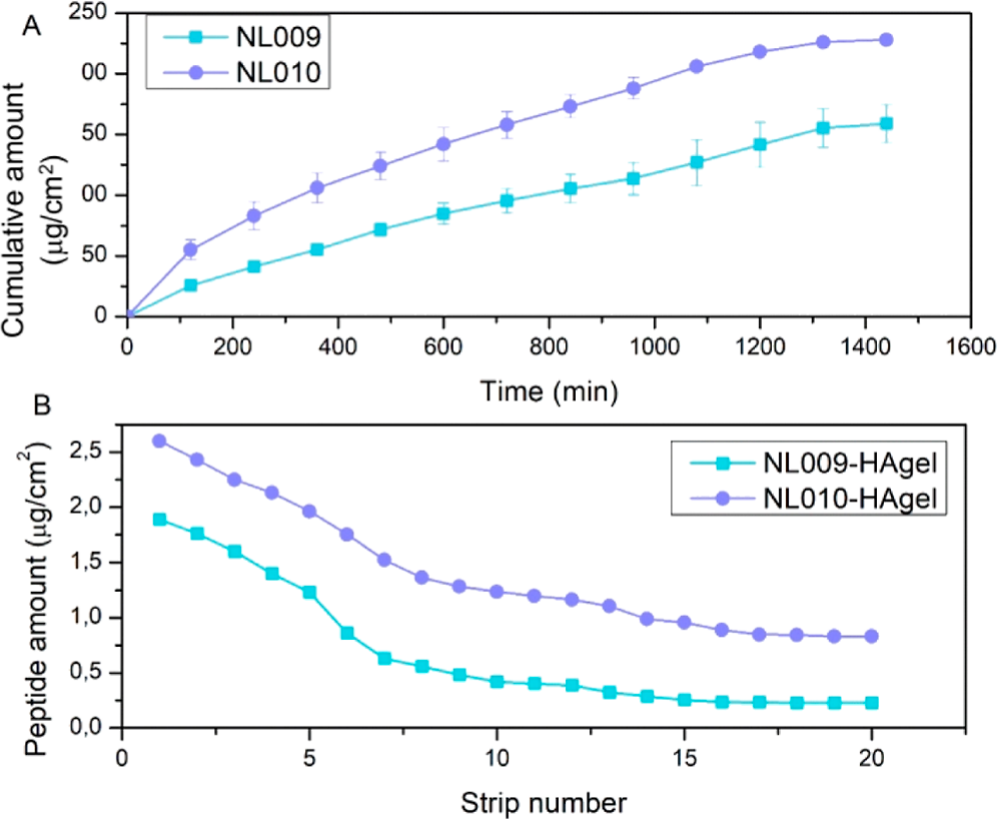
Quantitative evaluation of the ex vivo skin permeation studies. (A) Cumulative amount of peptide permeated from NL009-HAgel and NL010-HAgel and (B) accumulation within the skin layers via tape stripping (μg/cm^2^) for NL009-HAgel and NL010-HAgel.

### In Vitro Evaluation of Biocompatibility and Bioactivity of the CMP Hydrogels

To evaluate the bioactivity of the CMP hydrogels, we conducted cell viability and scratch assays using a human keratinocyte cell line (see Figure S21. Microscopic scratch closures). Both NL009-HAgel and NL010-HAgel were tested for their biocompatibility and effects on cell proliferation. Notably, NL010-HAgel demonstrated a statistically significant improvement compared to both the negative (*p* < 0.001) and positive controls. This compelling evidence led to the selection of NL010-HAgel as the primary dosage due to its safety and bioactivity. Overall, NL010-HAgel exhibited greater cell viability compared to NL009-HAgel across concentrations ranging from 0.01% to 0.5% w/v. However, at lower concentrations (0.01–0.05% w/v), both formulations showed minimal cell viability, even less than the negative control. At concentrations exceeding 0.1% w/v, a further decrease in cell viability was observed (Figure 10A).

**Figure 10:**
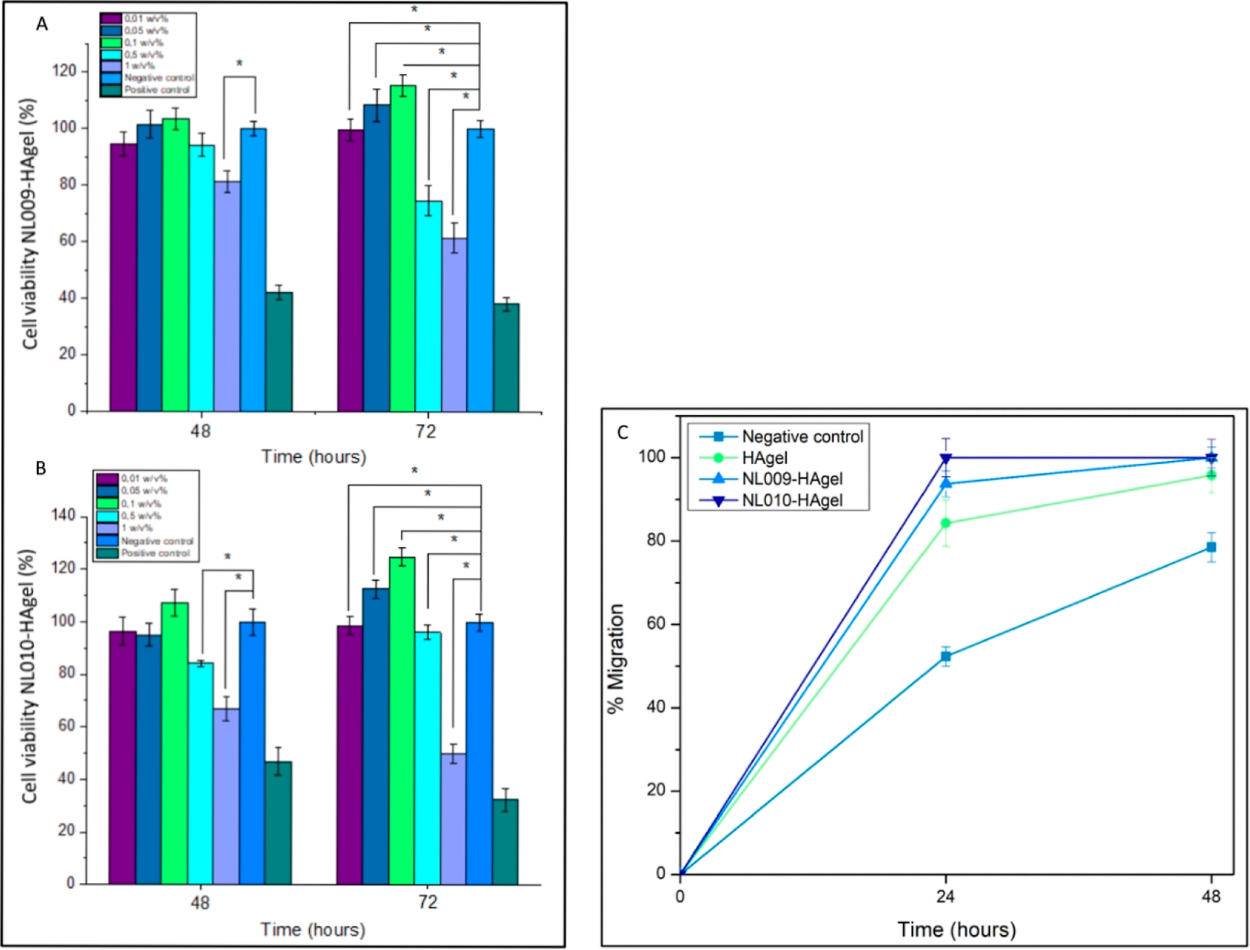
Cell viability (%) via an MTT assay for (A) NL009-HAgel and (B) NL010-HAgel over 48 and 72 h (*n* = 3) and (C) migration of the cells over time in relation to HAgel, NL009-HAgel, and NL010-Hagel in *HaCaT keratinocytes*.

Scratch assays were employed to quantify the bioactivity of the hydrogels by measuring the rate of scratch closure over time, represented as % migration (Figure 10B). Within 24 h, NL010-HAgel achieved a maximum migration of 99.99% ± 4.6%, while NL009-HAgel showed a migration rate of 93.7% ± 3.1%. HAgel alone reached 84.3% ± 5.6%, significantly outperforming the negative control, which showed only 52.3% ± 2.3% migration. Both NL009 and NL010-HAgels exhibited statistical significance over the negative control, with *p*-values <0.05. Additionally, NL010-HAgel showed significant results relative to HAgel (*p*-values <0.05). Although there was no statistical difference between NL009-HAgel and NL010-HAgel (*p* > 0.05), NL010-HAgel demonstrated the most substantial impact on cell migration. The maximum closure rates observed for the negative control and HAgel were 78.5% ± 3.5% and 95.8% ± 4.2%, respectively, at the 48 h mark (Figure 10C). These findings highlight the superior bioactivity of the CMP hydrogels, particularly NL010-HAgel, in promoting cell viability and migration, essential factors for effective wound healing. An opportunity for further development is the inclusion of coculturing keratinocytes and fibroblasts. Co-culturing these cell types has been shown to more accurately mimic the complex cellular interactions involved in wound healing and skin regeneration.^64^

Taking the cell viability and scratch assay into account, these in vitro findings supported the hypothesis that the CMPs have the ability to promote cell viability and do not hamper cell growth, as well as to enhance cell migration and scratch closure.^27^ NL010-HAgels were noted to promote cell proliferation and migration to a greater extent than the NL009-HAgel, thus evidencing this formulation as the superior optimal option.

### In Vivo Evaluation of Wound Closure Rates and Histopathological Analysis

Over the three day study period, the following processes were observed: the wound beds were red and exudating as a result of inflammation. In the negative control group, the wound beds were rounded and showed pus formation, as seen by the yellowing of the wound surface. The wounds were also moderately exudating. In the 7 day study, the wounds presented as dark red and lightly exudating. Wound exudate was more evident in the negative control and the HAgel groups. The wound beds showed almost complete closure with a central area of tissue that showed maturing tissue. For the negative control group, this central tissue presented as slightly red, which was indicative of ongoing healing and repair at this time point. The other groups, including the HAgel group, showed maturing tissue that presented as the skin tone (Figure 11A).

**Figure 11:**
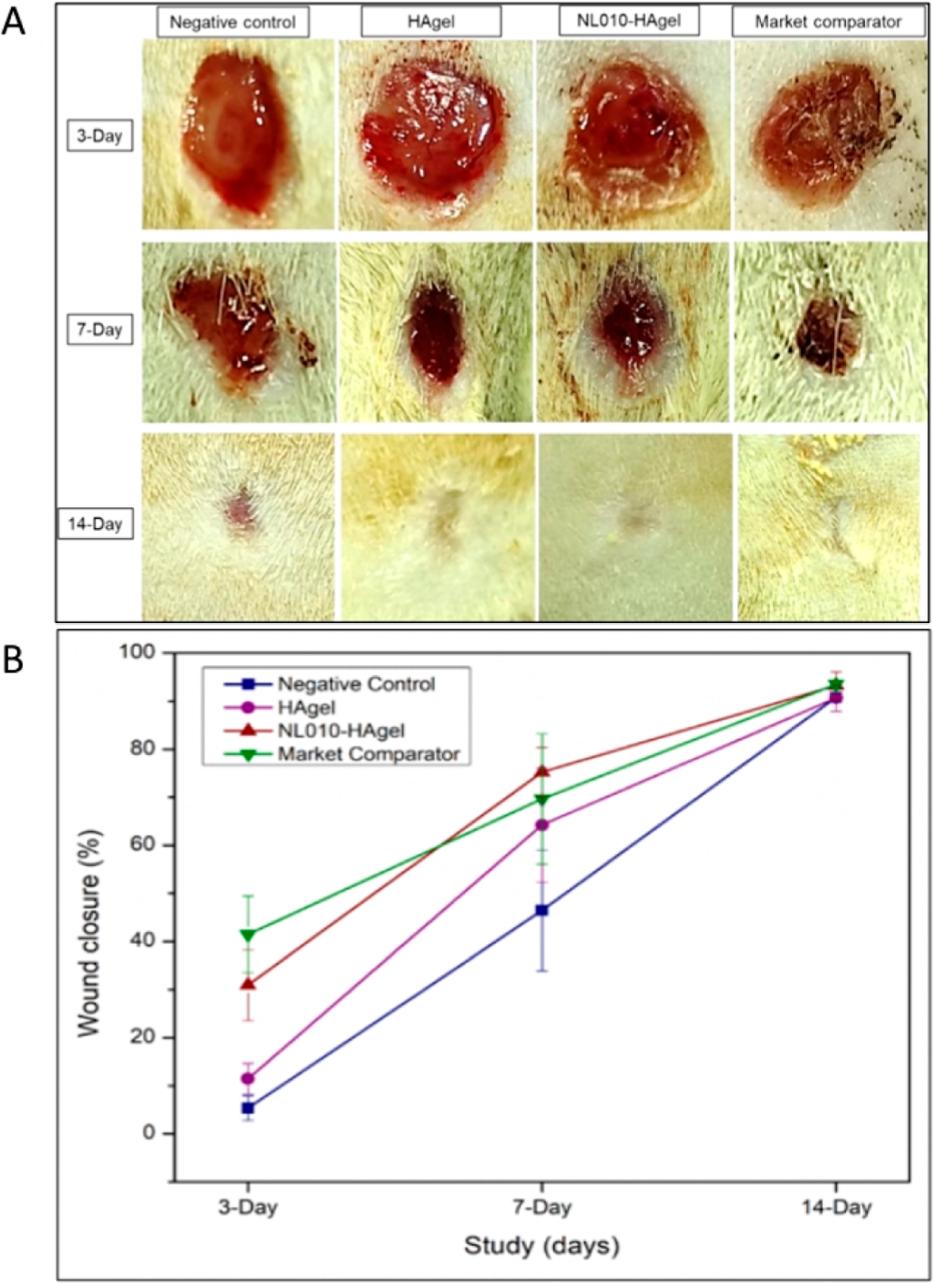
(A) Visual wound closure of rats with treatment of NL010-HAgel and compared to the market standard and negative control, HAgel, for a 3, 7, and 14 day period. (B) shows a quantitative wound closure %.

A quantitative assessment of wound closure was conducted, and the results are presented as a percentage in Figure 11B. At the 3 day time point, the Puramatrix group demonstrated the highest wound closure rate at 41.49 ± 7.98%, followed by the NL010-HAgel at 30.95 ± 7.36%. Statistically significant differences were observed among all groups (*p* < 0.05), except between the negative control and HAgel (*p* = 0.59914) and between NL010-HAgel and Puramatrix (*p* = 0.19633). Although NL010-HAgel showed a difference in % wound closure compared to Puramatrix, it was not statistically significant.

At the 7 day time point, NL010-HAgel showed improved closure at 75.24 ± 5.17%, while Puramatrix gel exhibited a closure rate of 69.66 ± 13.55%. The means were not statistically significant (*p* > 0.05). By the 14 day mark, Puramatrix gel showed 93.53 ± 0.83% wound closure, while NL010-HAgel recorded 93.26 ± 2.85%, with no significant difference (*p* > 0.05).

Histological examinations conducted after 3 days revealed that the NL010-HAgel (Figure 12A) presented as a linear, horizontal defect extending into the subcutis, filled with fibrinopurulent exudate and loose red blood cells. There was moderate infiltration of lymphocytes and macrophages along with mild epithelialization observed at the wound periphery, where the epithelial layers appeared hyperplastic. Histological assessment (Figure 12B) also showed a mild presence of haphazardly arranged thin collagen fibers, which appeared light blue.

**Figure 12:**
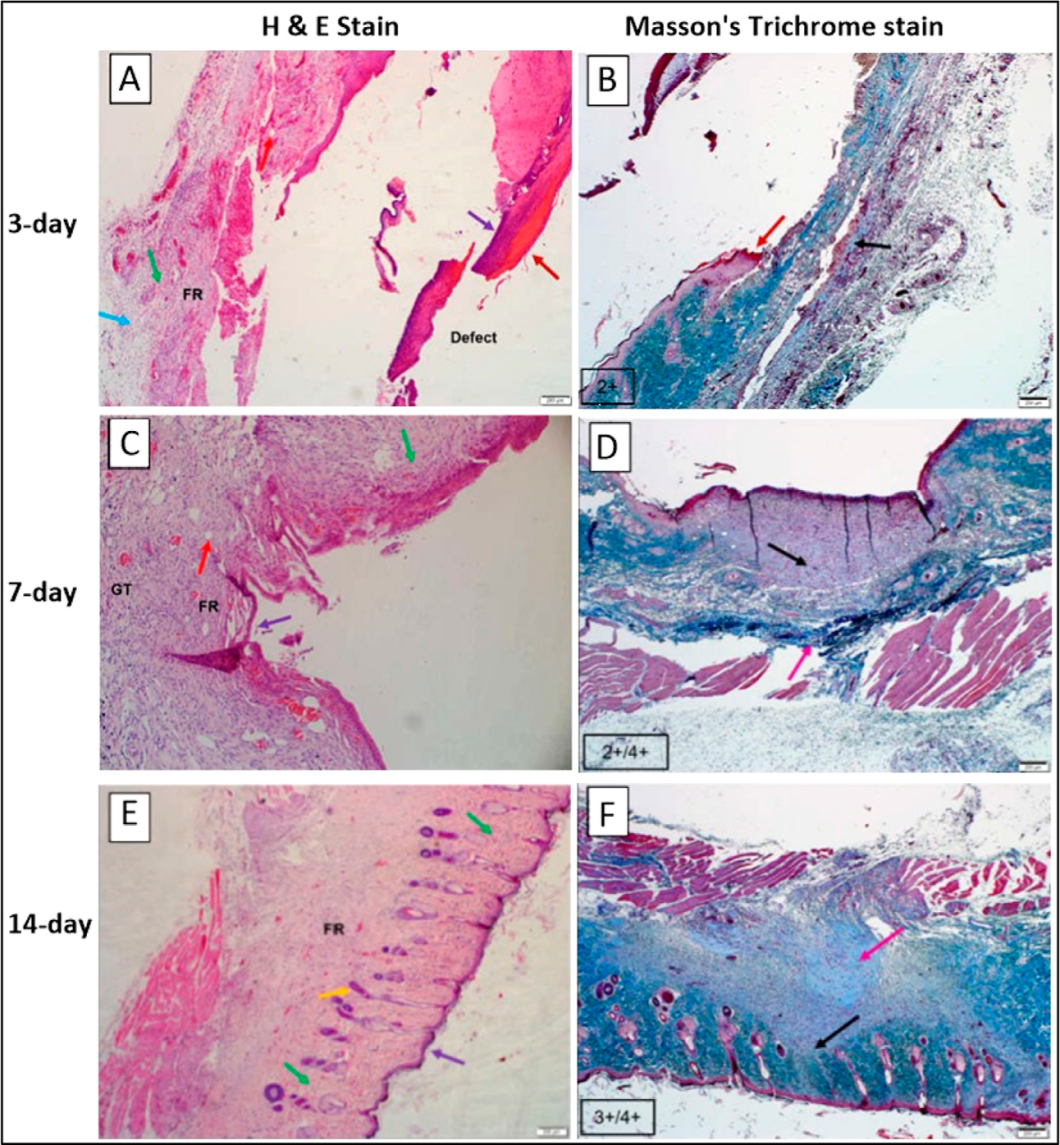
H&E stain and Masson’s trichrome stain on histology samples for the (A,B): 3 day, (C,D): 7 day, and (E,F): 14 day study. The images were viewed at a 4× magnification (scale bar 200 μM). FR: fibroblast region; GT: granulation tissue; dark red arrow: scab; yellow arrow: fibrinopurulent area; light-blue arrow: inflammatory cells; green arrow: collagen; red arrow: blood vessels; and purple arrow: epithelialization. Black arrow: collagen fibers and pink arrow: numerous prominent collagen fibers.

At the 7 day time point, the NL010-HAgel (Figure 12C) displayed a cup-shaped depressed invagination extending into the superficial and deep subcutis, filled with granulation tissue.

Moderate infiltration of fibroblasts was noted, showing both haphazard and horizontal (parallel) arrangements within the deep subcutis. Blood vessels were present within the fibroblastic reaction, indicating granulation tissue formation. Epithelialization was complete, with the epidermal layer extending across the fibroblastic region, which partially filled the defect. NL010-HAgel (Figure 12D) exhibited a mild presence of horizontally arranged collagen fibers in the superficial reaction, characterized by poor light-blue staining and medium thickness, along with a moderate number of nuclei.

After 14 days, NL010-HAgel (Figure 12E) displayed no visible defect. Fibroblasts were randomly arranged and associated with a moderate quantity of thin collagen fibers throughout the tissue. Single capillaries extended into the subcutaneous fibrosing reaction, with the epidermis intact and epithelialization complete, showing focal mild hyperplasia. Normal arrangement and presence of hair follicles were observed within the vertical linear dermal fibrosis. NL010-HAgel (Figure 12F) presented moderate amounts of haphazard to horizontally arranged collagen fibers in the superficial stromal reaction, characterized by thin light-blue staining and moderately prominent nuclei (fibroblasts). In the deeper reaction (deep subcutis), numerous and prominent dark-blue staining collagen fibers were observed, being thick and wavy with few nuclei/fibroblasts present.

Overall, the results indicate that NL010-HAgel demonstrates effective wound healing capabilities, promoting collagen deposition and tissue regeneration over time, comparable to those of the established Puramatrix standard. However, the combined effect of VEGF signaling and collagen deposition was not evident. A study by Saklani et al. recently showed that relative to angiogenic peptides (DYVRLAI and CDYVRLAI), angiogenic-collagen peptides (PGPIKVAV and Ac-PGPIKVAV) did not show any angiogenic signaling while comparable collagen deposition was observed.^65^

The prepared NL010-Hagel system demonstrates significant potential for treating infected or chronic wounds. It can efficiently encapsulate and deliver antimicrobial peptides, and CMPs are advantageous where ECM remodeling is impaired. Localized delivery reduces microbial burden, prevents infection, minimizes systemic toxicity, and enhances tissue regeneration, making it a promising strategy for managing complex wound environments. Hwang et al. recently demonstrated the potential of collagen-mimetic peptide (CMP)-modified nanocarriers for the sustained release of antimicrobial agents for chronic wound healing.^66^ In summary, this warrants further development of NL010-Hagel.

## Conclusions

This study evaluated a hyaluronic acid (HA) hydrogel embedded with collagen-mimetic peptides (CMPs) to develop functional biomaterials for wound healing. The CMPs exhibited stability in solution for 14 days, enabling the creation of a self-assembled, physically cross-linked hydrogel that functions effectively as a wound dressing. The formulation process was straightforward, yielding good reproducibility and minimal waste. HA’s excellent gelling properties, combined with its wound healing capabilities, facilitated the formation of a three-dimensional hydrogel characterized by a rapid sol–gel transition rate. Morphological analysis revealed a spongy bioplatform with micropores (<30 μm), ideal for wound dressing applications.

The physicomechanical properties of the CMP hydrogels showed optimal hardness, compressibility, adhesiveness, cohesiveness, and elasticity, making them suitable for maintaining prolonged contact with wound beds. Rheological assessments confirmed their viscoelastic nature and shear-thinning behavior. Both NL009 and NL010-HAgels demonstrated a high encapsulation efficiency for CMPs. In vitro bioactive release studies indicated an initial burst release within 12 h, followed by controlled release over 7 days, with NL010-HAgel showing superior skin permeation due to its lipophilic adamantane moiety. Cell-based studies on HaCaT keratinocytes revealed a dose-dependent relationship between CMP concentration and cell viability, with both hydrogels promoting faster wound closure compared to controls. Enhanced collagen production and deposition were observed at the 7 and 14 day marks, particularly with NL010-HAgel, which exhibited a more organized collagen fiber arrangement in the deeper dermis.

Future work should include an evaluation of protease stability and enzyme degradation parameters as these factors are crucial for understanding their impact on the system. To accurately assess cell migratory behavior, incorporating mitomycin C or a similar proliferation inhibitor will help isolate migration effects from cell division. Additionally, transcriptomic studies should be conducted to identify key metabolic pathways involved in wound healing, with findings validated through polymerase chain reaction (PCR) and RNA sequencing. A deeper understanding of signaling mechanisms at different healing stages will provide insights into CMP interactions and promote more effective wound healing strategies.

In conclusion, the benefits of the CMP hydrogel as a wound dressing include its ecofriendly self-assembly, mechanical and textural strength, controlled biodegradability, and enhanced bioactivity. The formulation significantly improved healing by promoting epithelialization and granulation tissue deposition through fibroblast and collagen responses, influencing key stages of wound healing: inflammation, proliferation, and re-epithelialization. NL010-HAgel demonstrated superior collagen synthesis compared to a market comparator, indicating its promising biological performance for in vivo wound dressing applications.

## Supplementary Material



## Notes

Details of the equipment used for measurements and analysis; overview of the methods applied during the experiments; step-by-step protocols followed in the experiments; list of the compounds synthesized for the study; tables showing characterization data; nuclear magnetic resonance (NMR) spectroscopy, mass spectrometry (MS), and circular dichroism (CD) results for the peptides; results of computational models used in the study; showing microscopic images of scratch closure assays for the peptide and peptide hydrogels; and rheology results for the peptide hydrogels (PDF)
