# Supplementary material for: Synthesis of a retro-GFOGER Adamantane-Based Collagen Mimetic Peptide Imbibed in a Hyaluronic Acid Hydrogel for Enhanced Wound Healing
Source: ACS Appl Bio Mater. 2025 Feb 19;8(6):4657–72. doi: 10.1021/acsabm.4c01895 (PMC12175121; doi:10.1021/acsabm.4c01895)
Supplement: Supplementary file 1 [file mt4c01895_si_001.pdf]

Supplementary Information

Synthesis of a *retro*-GFOGER adamantane-based collagen mimetic peptide imbibed in a hyaluronic acid hydrogel for enhanced wound healing

*Variksha Singh[1], Thashree Marimuthu[1], Ntlama F Lesotho[2]Maya M Makatini[2], Thandokuhle Ntombela[2], Armorel Van Eyk[3] and Yahya E Choonara[1]**

[1]*Wits Advanced Drug Delivery Platform Research Unit, Department of Pharmacy and Pharmacology, School of Therapeutic Sciences, Faculty of Health Sciences, University of the Witwatersrand, Johannesburg, 7 York Road, Parktown 2193, South Africa*

[2]*Molecular Sciences Institute, School of Chemistry, University of the Witwatersrand, Private Bag 3, PO WITS, 2050, South Africa.*

[3]*Division of Pharmacology, Department of Pharmacy and Pharmacology, School of Therapeutic Sciences, Faculty of Health Sciences, University of the Witwatersrand, Johannesburg, 7 York Road, Parktown 2193, South Africa*

*Correspondence: yahya.choonara@wits.ac.za

Contents

1. GENERAL INFORMATION 3

2. INSTRUMENTATION 3

3. GENERAL TECHNIQUES 4

4. GENERAL PROCEDURES 6

5. SUMMARY OF SYNTHESIZED COMPOUNDS 7

6. MASS SPECTRA OF SELECTED PEPTIDES 15

7. NMR SPECTRA OF SELECTED PEPTIDES 18

8. COMPUTATIONAL MODELLING DETAILS 29

9. Peptide bioactivity assessed on HaCaT keratinocytes 33

10. Peptide bioactivity assessed on 3T3 fibroblasts 34

11. Textural analysis 35

12. Peptide hydrogels bioactivity assessed on HaCaT keratinocytes 36

Electronic Supplementary Information (S1)

1. GENERAL INFORMATION

1.1 Chemicals and reagents

All organic solvents and reagents were obtained from commercial sources. Fmoc protected amino acids, (1-[Bis(dimethylamino)methylene]-1H-1,2,3-triazolo[4,5-b]pyridinium 3-oxide hexafluorophosphate, Hexafluorophosphate Azabenzotriazole Tetramethyl Uronium) (HATU), N,N,N′,N′-Tetramethyl-O-(1H-benzotriazol-1-yl)uraniumhexafluorophosphate (HBTU), and 2-chlorotrityl chloride resin were purchased from DLD scientific. Reagents such as diisopropylethylamine (DIPEA), piperidine, Triisopropylsilane (TIS), formic acid, trifluoroacetic acid (TFA), and adamantane were obtained from Sigma Aldrich (South Africa). Organic solvents such as dimethylformamide (DMF), HPLC grade methanol, HPLC grade acetonitrile, dimethylsulfoxide and diethylether were purchased from Radchem and Pyramid scientific.

2. INSTRUMENTATION

2.1 Ultra-High-Performance Liquid Chromatography Mass Spectrometry (UHPLC-MS)

Analytical LC-MS analysis was performed on an Ultra-High-Performance Liquid Chromatography (Thermo Scientific Ultimate 3000, RS diode array detectors) with a Diode Array (190, 195, 215, 254, and 300 nm) coupled to a Bruker Compact Q-TOF high-resolution mass spectrometer.

2.2 Spectroscopic and physical data

Nuclear magnetic resonance (NMR) spectra were recorded on either a Bruker AVANCE 300, 400 or Bruker AVANCE III 500 MHz spectrometer. Peptides were dissolved in deuteurated DMSO, $CDCl_3$ or d_6-acetone. Coupling constant (J-values are given in Hertz (Hz)). All chemical shift values are reported in parts per million referenced against trimethylsilane which is given an assignment of zero parts per million.

For conformational analysis, the experiment was conducted at different temperatures such as 300, 323 and 353 K on Bruker AVANCE 400 MHz instrument. For 2D experiments such as COSY, HSQC, HMBC, TOCSY, NOESY and ROESY, the spectra were recorded at the phase-

sensitive mode using time proportional phase increment (TPPI) on a Bruker III 500 MHz spectrometer. The NOESY experiments were recorded with mixing times of 150, 200 and 250 ms; the ROESY spectrum was recorded with mixing times of 100, 150, 200, 250 and 300 ms; while TOCSY was recorded with mixing times of 48, 64, 70, 80 and 100 ms. The residual water peak of DMSO-d6 was suppressed by a presaturation pulse of 2 s duration. 2048 data points were collected per experiment.

2.3 Circular dichroism

The circular dichroism spectra were recorded on a JASCO J-18 spectropolarimeter with the following settings; wavelength scan - 0.1 mm pathlength; 0.2 mg/mL protein concentration; 225 µl volume; 1.0 nm bandwidth; 0.5 nm resolution; 8 scans; 4 secs response; 20 nm/minute scan speed; scan range 280 – 160 nm while keeping HT voltage less than 600 V. The wavelength range was set between 190 to 250 nm while the temperature was set to 20 °C. The peptides were prepared in phosphate buffer at a concentration of 0.1 mg/mL. A 0.1 cm pathlength cuvette holding 300 µl was used for all CD measurements.

2.4 Ultra-violet Visible (UV-Vis)

Absorbance values used to determine the loading capacity of the resin were recorded on the Varian Cary Eclipse (Cary 50) UV-vis spectrophotometer.

3. GENERAL TECHNIQUES

3.1 UHPLC-MS Analysis

The samples were analysed using a binary solvent system where solvent A consisted of H_2O and 0.1% Formic acid (v/v), and solvent B consisted of Acetonitrile and 0.1% Formic acid (v/v). 20 µL of the sample was injected into a C18 column (5 µm, 100 Å, 4.60 mm × 150 mm). The system flow rate was set at 0.3 mL/minute in the positive mode. The mass spectrum analysis was processed using the Bruker Daltonics data analysis software. The ultra-pure water used in this study was obtained from the Millipore Direct-Q®3 UV water purification (ZRQSVPO30) system.

The samples were analysed in the positive ionization mode and the MS was scanned at m/z 100-3000 range during separation and detection. Nitrogen gas (N_2) was used as the dry and

nebulizer gas. The nebuliser gas was operated at a pressure of 1.8 bars whereas the drying gas was operated at a flow rate of 9 L/minute.

3.2 ^{1}H Nuclear Magnetic Resonance (^{1}H NMR)

The chemical shifts (δ) values are reported in parts per million (ppm) relative to deuterated dimethyl sulfoxide (DMSO-d_6, 2.49 ppm) and referenced against the internal standard, tetramethylsilane (TMS, 0.00 ppm). The spectra were analyzed as first order and the values of the coupling constant (J) are reported as Hertz (Hz). Multiplicity of signals is expressed as: s = singlet, br s = broad singlet, d = doublet, dd = double of doublet, t = triplet, q = quartet, m = multiplet.

3.3 ^{13}C NMR

The chemical shift values are reported in (ppm) relative to deuterated (DMSO-d_6, 39.52 ppm) and TMS as an internal standard.

3.4 2D NMR

Spectra for COSY, ^{13}C-HSQC, TOCSY, NOESY and ROESY experiments, were recorded at 293, 300, and 308 K on a 500 MHz NMR Bruker III 500 MHz spectrometer. All 2D spectra were recorded at the phase-sensitive mode using time proportional phase increment (TPPI). The residual water peak of DMSO-d_6 was suppressed by a pre-saturation pulse of 2.0 *s* duration. The first NOESY experiments were recorded with mixing time of 150, 200 and 250 ms; the ROESY spectrum was recorded with mixing times of 100, 150, 154 200, 250 and 300 ms; while TOCSY was recorded with mixing times of 48, 64, 70, 80 and 100 ms; each increment was the sum of 32 scans with a relaxation delay of 2.0 s; 2048 data point were collected per experiment.

4. GENERAL PROCEDURES

4.1 General procedure A: Activation and coupling of the first amino acid to 2-chlorotitryl resin.

2-Chlorotrityl-chloride resin (600 mg, 0.174 mmol/g) was added to a 50 mL centrifuge tube. Dry DCM (10 mL) and 2mL of thionyl chloride were added to the tube. The mixture was shaken overnight then filtered using suction and washed with dry DCM (5 × 4 mL). To avoid deactivation of the water-sensitive resin, DCM (10 mL), 1 mL of DIPEA (1.0 M, 2.0 mL) and the first amino acid (0.987g, 0.2 M) were immediately added to the dry resin in a sintered-glass reaction vessel. This mixture was reacted for 2 hours and then dried using suction. Finally, the resin was washed with DCM (3 x 5 mL).

4.2 General procedure B: Analysis of the substitution of the first amino acid

The resin, with the first amino acid bonded, was weighed out in duplicate samples of 5 to 10 mg in Eppendorf tubes then 20% piperidine in DMF (1.0 mL) was added to the resin to deprotect the amino acid. The resin and was shaken for 20 minutes then centrifuged down. From the centrifuged sample, 100 µL of the supernatant was transferred into a tube with DMF (10.0 mL). The mixture was thoroughly mixed, and the solution (2.0 mL) was pipetted into one cuvette cell and DMF (2.0 mL) was added into another cuvette cell. The cuvette containing DMF was used to zero the spectrophotometer. The sample was checked for absorbance at 301 nm three times and used to calculate the substitution using the following equation:

$$Loading\ capacity\left(\frac{mmol}{g}\right) of\ resin = \frac{(101 \times Average\ Absorbance)}{(7.8 \times mg\ of\ resin\ beads)}$$

The substitution of fmoc-amino acid loaded was then used to calculate the loading capacity and the theoretical yield:

Theoretical yield = sub (mmol/g) × mass of resin (g) × Mr of peptide (g/mol)

5. SUMMARY OF SYNTHESIZED COMPOUNDS

5.1 DGD-GG-GFOGER-GG-Adamantane (NL008)

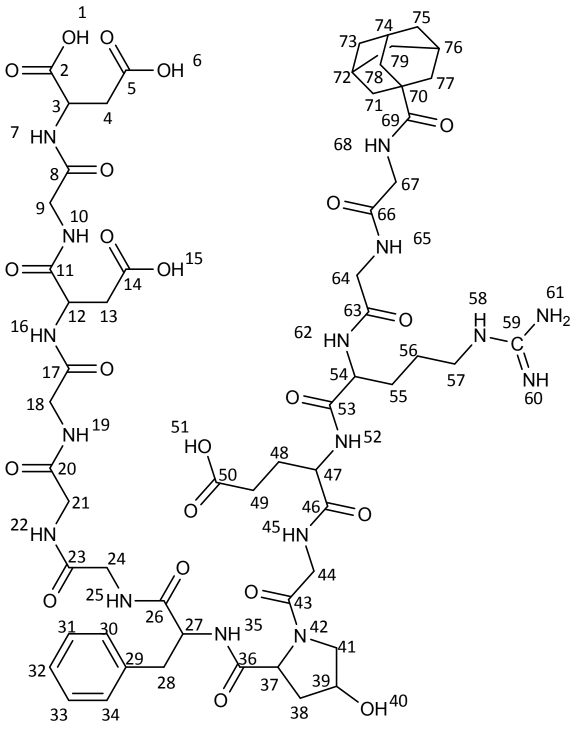

Figure S1. DGD-GG-GFOGER-GG-Adamantate

UHPLC (Thermo Scientific Dionex Ultimate 3000) Peak 4.91 minutes

HRMS (ESI) (Bruker Compact Q-TOF): Calculated for $C_{58}H_{82}N_{16}O_{22}$ = 1354.5790; found 1355.5960 ([M + H^+]).

^{1}H NMR (400 MHz, DMSO) δH = 8.66 (1H, m, NH amide), 8.53 (1H, m, NH amide), 8.27 (1H, m, NH amide), 8.26 (1H, m, NH amide), 8.25 (2H, m, NH amide), 8.09 (1H, m, NH amide), 8.08 (2H, m, NH amide), 8.01 (2H, m, NH amide), 7.94 (1H, m, NH amide), 7.78 (1H, m, NH amide), 7.26 (1H, m, ArH), 7.19 (1H, m, ArH), 7.14 (1H, m, ArH), 4.72 (1H, m, Hα Phe), 4.65 (1H, m, Hα Asp), 4.64 (1H, m, Hα Asp), 4.58 (1H, m, Hα Gly), 4.44 (1H, m, Hα Hyp), 4.41 (1H, m, Hα Gly), 4.33 (1H, m, Hα Arg), 4.30 (1H, m, Hα Gly), 4.24 (1H, m, Hα Hyp), 4.02 (1H, m, Hα Gly), 3.78 (1H, m, Hα Gly), 3.71 (1H, m, Hα Gly), 3.67 (1H, m, Hα Gly), 3.60 (1H, m, Hα Hyp), 3.59 (1H, m, Hα Glu), 3.34 (1H, m, Hβ Glu), 3.10 (2H, m, Hβ Asp), 3.10 (1H, m, Hβ Asp), 3.09 (1H, m, Hβ Arg), 2.87 (1H, m, Hβ Glu), 2.79 (1H, m, Hβ Phe), 1.91 (1H, m, Hγ Hyp), 1.86 (1H, m, Hγ Hyp), 1.65 (2H, m, Adamantane), 1.63 (3H, m, Adamantane), 1.62 (3H, m, Adamantane), 1.59 (2H, m, Adamantane), 1.57 (2H, m, Adamantane), 1.51 (2H, m, Hβ Arg)

^{13}**C NMR** (400 MHz, DMSO) δC = 174.78 (C = O), 173.86 (C = O), 172.92 (C = O), 172.62 (C = O), 172.05 (C = O), 171.79 (C = O), 171.76 (C = O), 171.53 (C = O), 170.99 (C = O), 170.51 (C = O), 169.62 (C = O), 169.35 (C = O), 169.19 (C = O), 168.41 (C = O), 163.90 (C = NH), 129.61 (ArC), 128.50 (ArC), 129.50 (ArC), 69.02 (OH-C-H), 66.99 (Cα), 66.38 (Cβ Hyp), 56.50 (Cα), 52.79 (Cα), 52.45 (Cα), 42.53 (Cα), 36.60 (Cβ), 30.80 (Cβ), 31.01 (Cβ), 29.60 (C-Aliphatic), 28.10 (C-Aliphatic), 27.06 (C-Aliphatic), 22.83 (C-Aliphatic), 20.07 (C-Aliphatic), 19.81 (C-Aliphatic), 19.01 (C-Aliphatic), 15.63 (C-Aliphatic)

Table S1. Characterization table of DGD-GG-GFOGER-GG-Adamantane

No.	δ ^{1}H	δ ^{13}C	δ COSY	δ HSQC	δ HMBC
1	11	-	-	-	-
2	-	54.30	-	54.30, 4.64	54.30, 8.66
3	4.64	54.30	4.64, 8.66	4.64, 54.30	4.64, 162.49
4	3.10	37.40	3.10, 4.65	3.10, 37.40	3.10, 165.43
5					165.43, 3.10
6	11	-	-	-	-
7	8.66	-	8.66, 4.64	-	8.66, 169.98
8					166.56, 4.41
9	4.41	58.01	4.41, 8.58	4.41, 58.01	4.41, 166.56
10	8.53	-	8.53, 4.41	-	8.53, 166.23
11					175.64, 4.65
12	4.65	54.30	4.65, 3.10	4.65, 54.30	4.64, 162.49
13	3.10	37.40	3.10, 4.65	3.1037.40	3.10, 165.43
14					165.43, 3.10
15	11	-	-	-	-
16	8.27	-	8.27, 4.65	-	8.26, 169.28
17					169.58, 3.78
18	3.78	42.39	3.78, 8.26	3.78, 42.39	3.78, 169.42
19	8.26	-	8.26, 3.78	-	8.26, 169.28
20					169.66, 3.71
21	3.71	54.30	3.71, 8.09	3.71, 54.30	3.71, 169.42
22	8.09	-	8.09, 3.71	-	8.09, 169.02
23					172.69, 4.58
24	4.58	49.66	4.58, 8.08	4.58, 49.66	4.58, 171.82

No.	δ ^{1}H	δ ^{13}C	δ COSY	δ HSQC	δ HMBC
25	8.08	-	8.08, 4.58	-	8.08, 171.29
26					165.18, 4.76
27	4.76	60.00	4.76, 3.10	4.65, 60.00	4.76, 165.18
28	3.10<br>2.86	36.57	3.10, 4.65	3.10, 36.57	2.82, 168.47
29	-	138.51	-	-	138.51, 7.26
30	7.14	126.66	7.14, 7.26	7.14, 126.15	7.14, 138.51
31	7.26	129.66	7.26, 7.14	7.26, 129.45	7.26, 138.51
32	7.18	128.50	7.18, 7.26	7.18, 127.84	7.18, 138.51
33	7.26	129.66	7.26, 7.14	7.26, 129.45	7.26, 138.51
34	7.14	126.66	7.14, 7.26	7.14, 126.15	7.26, 138.51
35	8.03	-	8.03, 4.72	-	8.05, 171.60
36					168.65, 4.17
37	4.17	67.35	4.17, 1.89	4.17, 67.35	4.17, 168.65
38	1.85	38.03	1.85, 4.24	1.85, 38.03	1.85, 172.40
39	4.24	69.00	4.24, 3.60	4.24, 69.00	4.24, 33.73
40	3.54				3.54, 69.38
41	3.60	54.33	3.60, 4.24	3.60, 54.33	3.61, 69.95
42	-	-	-	-	-
43	-		-	-	171.79, 4.17
44	4.30	59.23	4.30, 8.25	4.30, 59.23	4.30, 171.79
45	8.25	-	8.25, 4.30	-	8.25, 169.28-
46	-	-	-	-	168.68, 3.59
47	3.59	54.47	3.59, 7.78	3.59, 54.47	3.59, 168.68
48	2.43	37.32	2.43, 3.59	2.43, 37.32	2.43, 54.39
49	3.34	54.48	3.34, 2.87	3.34, 54.48	3.35, 37.56
50	-	-	-	-	170.75, 3.32
51	11	-	-	-	-
52	7.79	-	7.79, 3.59	-	7.79, 168.53-
53	-	-	-	-	171.91, 4.33-
54	4.33	52.34	4.33, 8.01	4.33, 52.34	4.33, 171.91
55	1.91<br>1.97	37.92	1.91, 4.33	1.91, 37.92	1.95, 28.25
56	1.51<br>1.62	29.25	1.51, 1.91	1.51, 29.25	1.55, 36.75

No.	δ ^{1}H	δ ^{13}C	δ COSY	δ HSQC	δ HMBC
57	3.09	40.77	3.09, 7.54	3.09, 40.77	3.09, 28.25
58	7.54		7.54, 3.09		7.56, 158.20
59	-	157.31	-	-	-
60	-	-	-	-	-
61	-	-	-	-	-
62	8.01	-	8.01, 4.33	-	8.01, 169.33
63					165.75, 4.01
64	4.01<br>3.84	42.02	4.02, 8.00	4.01, 42.02	4.01, 165.75
65	8.01	-	8.01, 4.04	-	8.01, 169.33
66					170.22, 3.67
67	3.67	49.08	4.53, 8.48	4.53, 49.08	4.55, 172.97
68	7.94	-	7.94, 3.67	-	7.94, 170.19
69					176.78, 1.60
70		36.33	-	-	36.33, 1.60
71	1.60	38.90	1.60, 4.31	1.60, 38.92	1.60, 176.78
72	1.63	30.27	1.63, 3.02	1.63, 30.27	1.63, 39.11
73	1.53	36.51	1.53, 3.02	1.56, 36.53	1.53, 36.72

5.2 DGD-GG-GFOGER-GG-TTK-Adamantane (NL010)

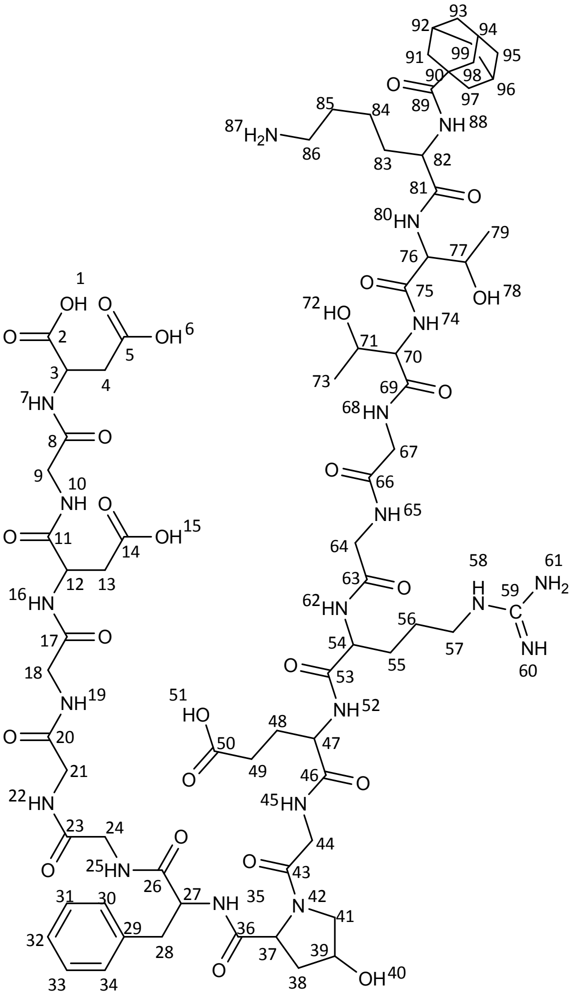

Figure S2: DGD-GG-GFOGER-GG-TTK-Adamantate

UHPLC (Thermo Scientific Dionex Ultimate 3000) Peak 6.01 minutes

HRMS (ESI) (Bruker Compact Q-TOF): Calculated for $C_{72}H_{108}N_{20}O_{27}$ = 1684.7693; found 843.3929 ($[M + 2H]^{2+}$).

^{1}H NMR (400 MHz, DMSO) δ_H = 8.60 (1H, m, NH amide), 8.54 (1H, *m*, NH amide), 8.60 (1H, *m*, NH amide), 8.36 (1H, *m*, NH amide), 8.13 (1H, *m*, NH amide), 8.15 (1H, *m*, NH amide), 8.14 (1H, *m*, NH amide), 8.32 (1H, *m*, NH amide), 8.23 (1H, *m*, NH amide), 7.69 (2H, *m*, NH amide), 8.12 (1H, *m*, NH amide), 7.79 (1H, *m*, NH amide), 7.79 (1H, *m*, NH amide), 7.99 (1H, *m*, NH amide), 7.14 (2H, *m*, ArH), 7.27 (2H, *m*, ArH), 7.17 (1H, *m*, ArH), 4.63 (2H, *m*, H$^\alpha$ Asp), 4.41 (1H, *m*, H$^\alpha$ Gly), 3.80 (1H, *m*, H$^\alpha$ Gly), 3.77 (1H, *m*, H$^\alpha$ Gly), 4.54 (1H, *m*, H$^\alpha$ Gly), 4.66 (1H, *m*, H$^\alpha$ Phe), 4.43 (1H, *m*, H$^\alpha$ Hyp), 4.27 (1H, *m*, H$^\alpha$ Gly), 4.33 (1H, *m*, H$^\alpha$ Glu), 4.31 (1H, *m*, H$^\alpha$ Arg), 4.06

(1H, *m,* H^{α} Gly), 4.24 (1H, *m*, H^{α} Gly), 3.82 (2H, *m*, H^{α} Thr), 4.35 (1H, m, H^{α} Lys), 2.81 (2H, m, H^{β} Asp), 2.85 (1H, *m,* H^{β} Phe), 1.90 (1H, *m*, H^{β} Arg), 4.23 (1H, *m*, H^{γ} Hyp), 3.61 (1H, *m*, H^{δ} Hyp), 2.53 (1H, *m*, H^{β} Glu), 3.05 (1H, m, H^{γ} Glu), 1.89 (1H, m, H^{α} Arg), 1.51 (1H, *m*, H^{β} Arg), 3.00 (1H, *m*, H^{γ} Arg), 8.82 (1H, *m*, NH guanidyl), 4.04 (2H, *m*, H^{β} Thr), 4.32 (2H, m, OH Thr), 1.04 m, (2H, *m*, H^{γ} Thr), 1.88 (1H, *m*, H^{β}), 1.57 (1H, *m*, H^{γ}), 1.73 (1H, *m*, H^{δ}), 2.27 (1H, *m*, H^{ϵ})

^{13}C NMR (400 MHz, DMSO) δ_C = 174.78 (C = O), 172.72 (C = O), 172.67 (C = O), 172.05 (C = O), 171.80 (C = O), 170.99 (C = O), 170.51 (C = O), 169.35 (C = O), 169.19 (C = O), 168.41 (C = O), 163.90 (C = O), 129.61 (ArC), 128.50 (ArC), 69.02 (C^{α}), 66.99 (C^{α}), 65.38 (C^{α}), 56.50 (C^{α}), 52.80 (C^{α}), 52.45 (C^{α}), 42.53 (C^{β}), 40.88 (C^{β}), 39.18 (C - Aliphatic), 38.98 (C - Aliphatic), 36.56 (C - Aliphatic), 31.07 (C - Aliphatic), 30.80 (C - Aliphatic), 29.60 (C - Aliphatic), 28.10 (C - Aliphatic), 27.06 (C - Aliphatic), 22.83 (C - Aliphatic), 20.07 (C - Aliphatic), 19.80 (C - Aliphatic), 19.01 (C - Aliphatic), 15.63 (C - Aliphatic)

Table S2. DGD-GG-GFOGER-GG-TTK-Adamantane characterization table

No.	δ ^{1}H	δ ^{13}C	δ COSY	δ HSQC	δ HMBC
1	11.0	-	-	-	-
2	-	-	-	-	165.63, 4.63-
3	4.63	54.22	4.63, 8.60	4.63, 54.22	4.63, 165.63
4	2.81	40.77	2.81, 4.63	2.81, 40.77	2.81,54.66
5	-	-	-	-	170.30, 2.83-
6	11.0	-	-	-	-
7	8.60	-	8.59, 4.63	-	8.56, 171.72
8	-				166.73, 4.41
9	4.41	54.47	4.41, 8.54	4.41, 54.47	4.41, 166.68
10	8.54		8.54, 4.41	-	8.54, 179.77
11	-				165.63, 4.63
12	4.63	54.22	4.63, 8.60	4.63, 54.22	4.63,135.63
13	2.81	40.77	4.63, 2.81	2.81, 40.77	2.81, 54.66
14	-	-	-	-	170.30, 2.83-
15	11.0	-	-	-	
16	8.60		8.60, 4.63	-	8.60, 171.15
17	-				171.97, 3.80
18	3.80	49.33	3.80, 8.36	3.80, 49.33	3.80, 171.97
19	8.36	-	8.36, 3.80	-	8.36, 163.90
20	-	-	-	-	169.85, 3.77-
21	3.77	44.91	3.77, 8.13	3.77, 44.91	3.77, 169.88
22	8.13	-	8.13, 3.77	-	8.13, 171.26
23	-	-	-	-	171.48, 4.54-
24	4.54	50.22	4.54, 8.15	4.54, 50.22	4.54, 171.36
25	8.15	-	8.15, 4.54	-	8.15, 177.49

26	-	-	-	-	171.34, 4.66-
27	4.66	58.53	4.66, 8.14	4.66, 58.64	4.66, 178.07
28	2.85	38.25	2.81, 4.65	2.85, 38.25	2.85, 179.66
29	-	138.19	-	-	138.19, 7.19
30	7.14	127.84	7.14, 7.27	7.14, 126.71	7.14, 126.71, 36.92, 138.11
31	7.27	129.49	7.27, 7.17	7.27, 129.39	
32	7.17	126.15	7.17, 7.27	7.17, 128.86	
33	7.27	129.49	7.27, 7.17	7.27, 129.39	
34	7.14	127.84	7.14, 7.27	7.14, 126.71	7.14, 126.71
35	8.14	-	8.14, 4.66	-	8.14, 171.16
36	-				172.69, 4.43
37	4.43	54.47	4.43, 8.32	4.43, 54.47	4.53, 171.85
38	1.90	37.53	1.90, 4.23	1.90, 37.53	1.89, 69.04
39	4.23	69.03	4.23, 3.61	4.23, 69.03	4.23, 67.03
40	3.58	-	-	-	3.55, 69.21
41	3.61	54.48	3.61, 4.23	3.61, 54.48	3.62, 65.08
42	-	-	-	-	
43	-				
44	4.27	49.35	4.27, 8.32	4.27, 49.35	4.27, 172.20
**No.**	**δ ^{1}H**	**δ ^{13}C**	**δ COSY**	**δ HSQC**	**δ HMBC**
45	8.32	-	8.32, 4.27	-	8.32, 175.43
46	-				172.31, 4.32
47	4.33	52.51	4.33, 8.23	4.33, 52.51	4.33, 172.03
48	2.53	40.16	2.53, 4.20	2.53, 40.16	2.53, 49.32
49	3.05	40.77	3.05, 2.52	3.05, 40.77	2.02, 40.16
50	-	-	-	-	165.94, 3.04-
51	11.0	-	-	-	
52	8.23	-	8.23, 4.33	-	8.23, 170.07
53	-	-	-	-	170.55, 4.31-
54	4.31	52.51	4.31, 7.69	4.31, 52.51	4.31, 172.29
55	1.89	28.03	1.89, 4.31	1.89, 52.68	1.92, 169.12
56	1.51	59.42	1.51, 1.89	1.51, 59.42	1.51, 28.03
57	3.00	55.35	3.02, 1.51	3.02, 55.35	3.02, 59.42
58	8.82	-	8.82, 3.07	-	8.82, 55.35
59					156.80, 8.82
60	-	-	-	-	-
61					
62	7.69		7.69, 4.31	-	7.69, 170.62
63					174.00, 4.07
64	4.06	52.60	4.06, 7.69	4.06, 52.60	4.06, 170.53
65	7.69		7.69, 4.07	-	7.69, 170.62
66	-	-	-	-	170.88, 4.23
67	4.24	58.76	4.24, 8.12	4.24, 58.76	4.24, 171.09
68	8.12	-	8.12, 4.24	-	8.12, 173.58
69	-	-	-	-	169.58, 3.82-
70	3.82	52.90	3.82, 7.99	3.82, 52.90	3.82, 169.68
71	4.04	66.87	3.82, 4.04	4.04, 66.87	4.04,

72	4.32				4.32, 66.87
73	1.04	19.79	1.04, 4.04	1.04, 19.79	1.04, 66.87
74	7.79	-	7.79, 3.82	-	7.79, 179.67
75	-	-	-	-	169.58, 3.82--
76	3.82	52.90	3.82, 7.99	3.82, 52.90	3.82, 169.58
77	4.04	66.87	3.82, 4.04	4.04, 66.87	4.04,
78	4.32				4.32, 66.87
79	1.04	19.79	1.04, 4.04	1.04, 19.79	1.04, 66.87
80	7.79	-	7.79, 3.82	-	7.79, 179.67
81	-	-	-	-	-
82	4.35	59.45	4.35, 7.99	4.35, 59.45	4.35, 172.31
83	7.99	-	7.99, 4.35	-	7.79, 179.67
84	1.88	37.91	1.88, 4.29	1.88, 37.91	1.88, 37.23
85	1.57	37.65	1.57, 1.88	1.57, 37.65	1.57, 36.64
86	1.73	29.14	1.73, 1.57	1.73, 29.14	1.73, 40.50
87	2.27	31.08	2.27, 1.73	2.27, 31.08	2.27, 25.33
88	-	-	-	-	-
89	-	-	-	-	178.05, 1.60
90	-	36.33	-	-	36.33, 1.60
91	1.60	38.90	1.60, 4.31	1.60, 38.92	1.60, 36.33
**No.**	**δ ^{1}H**	**δ ^{13}C**	**δ COSY**	**δ HSQC**	**δ HMBC**
92	1.63	30.27	1.63, 3.02	1.63, 30.27	1.60, 176.78
93	1.53	36.51	1.53, 3.02	1.56, 36.53	1.53, 37.53
94	1.63	30.27	1.63, 3.02	1.63, 30.27	1.65, 36.65
95	1.63	36.49	1.53, 3.02	1.63, 36.49	1.63, 39.08
96	1.63	30.27	1.63, 3.02	1.63, 30.27	1.63, 38.08
97	1.67	38.94	1.67, 4.31	1.67, 38.94	1.70, 39.10
98	1.63	36.50	1.53, 3.02	1.63, 36.50	1.67, 176.78
99	1.67	38.94	1.67, 4.31	1.67, 38.94	1.67, 176.78

6. MASS SPECTRA OF SELECTED PEPTIDES

6.1 DGD-GG-GFOGER-GG-Adamantane (NL008)

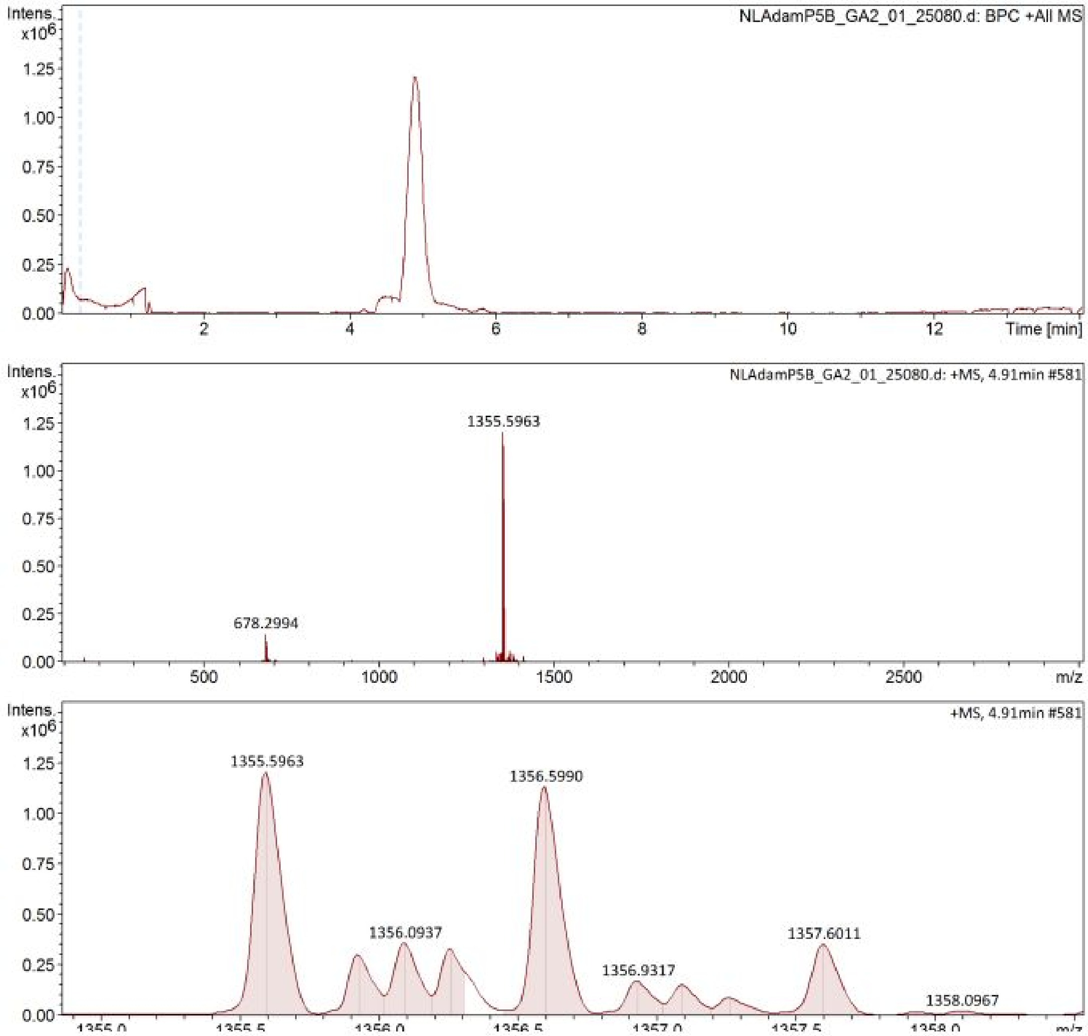

Figure S3: Mass spectrum of DGD-GG-GFOGER-GG-Adamantate

6.2 DGD-GG-GFOGER-GG-TTK-Adamantate (NL010)

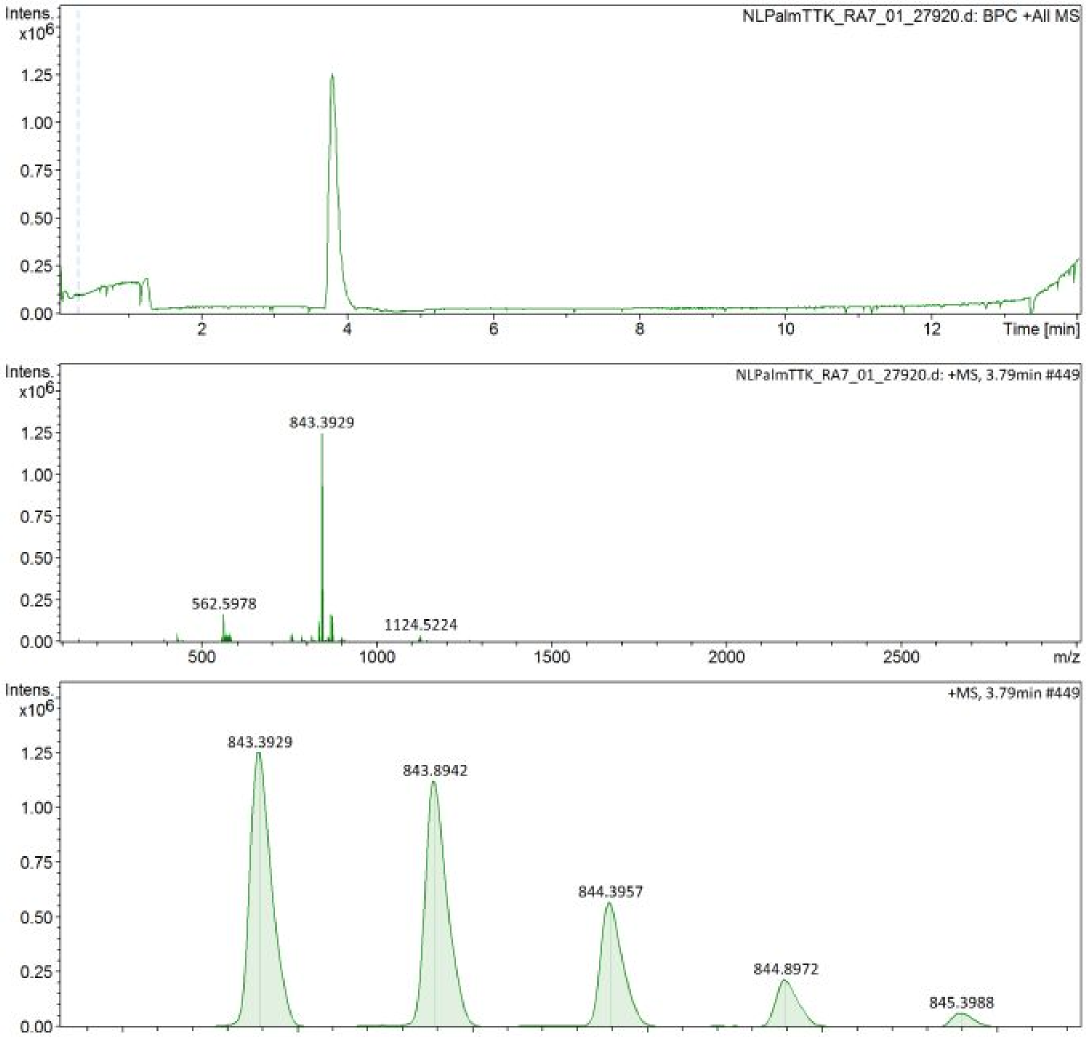

Figure S4.1: Mass spectrum of DGD-GG-GFOGER-GG-TTK-Adamantate

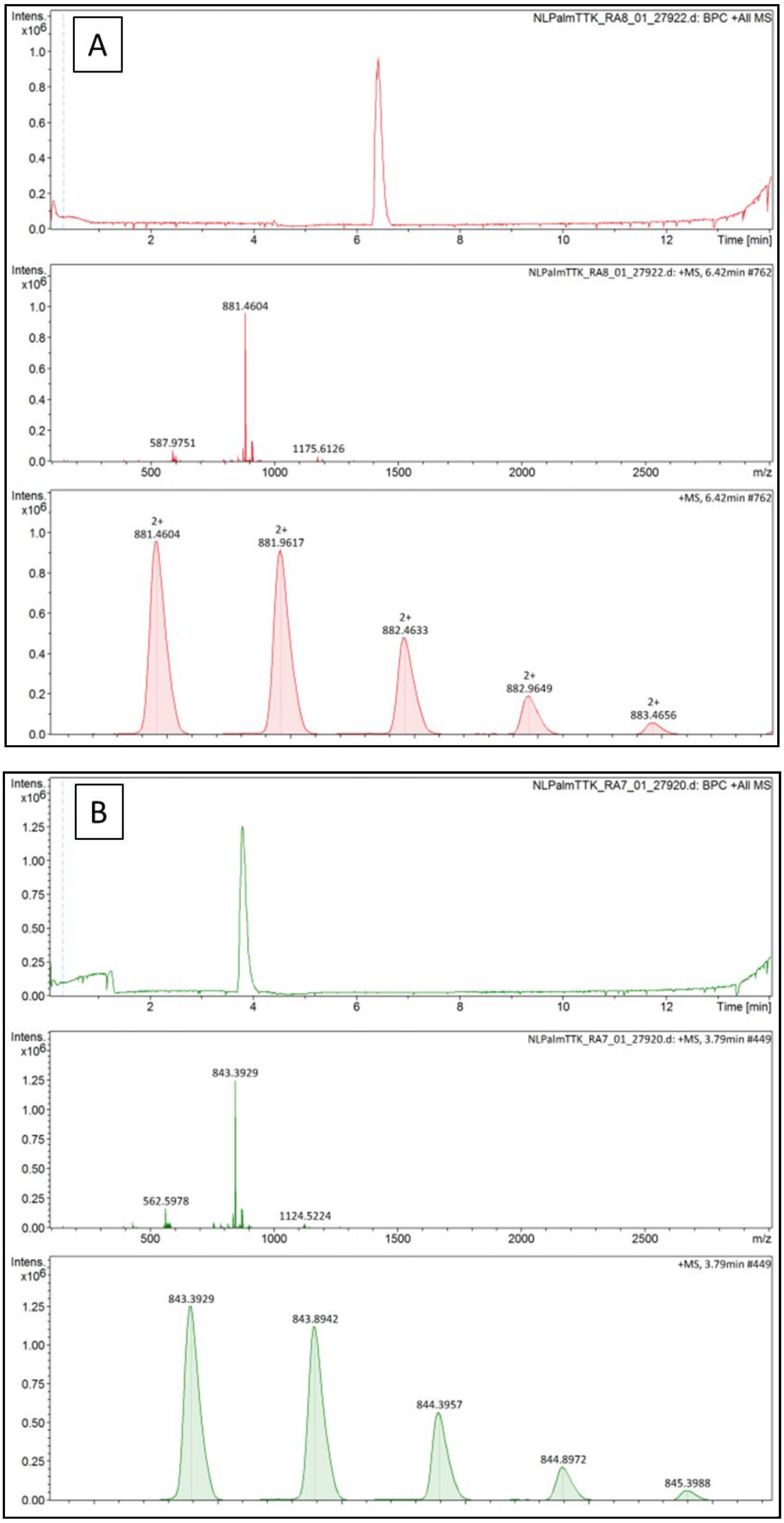

Figure S4.2 HPLC-MS spectrum of A) NL009 and B) NL010 after 14 days.

7. NMR SPECTRA OF SELECTED PEPTIDES

a)

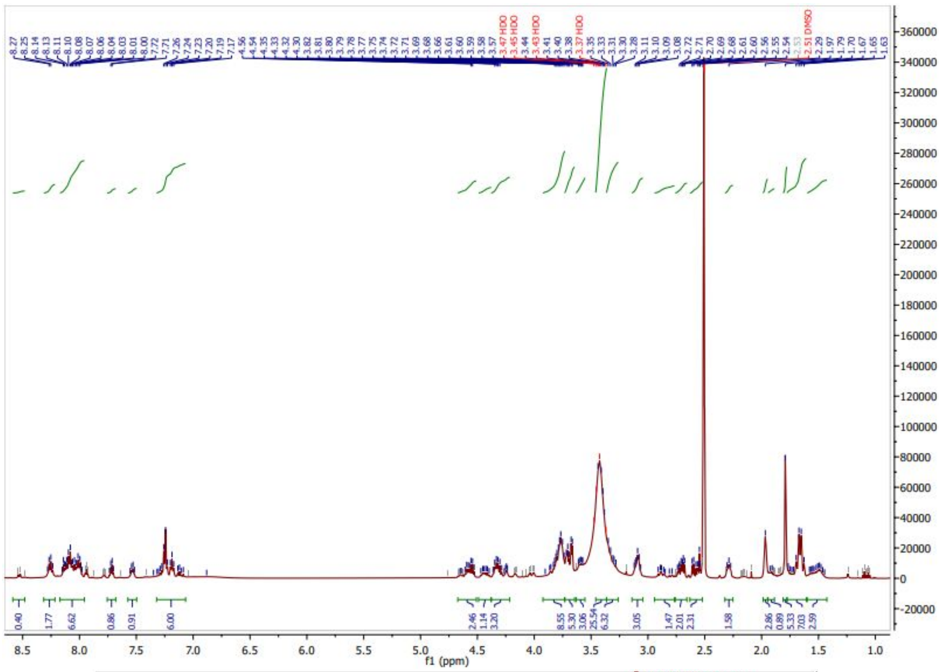

b)

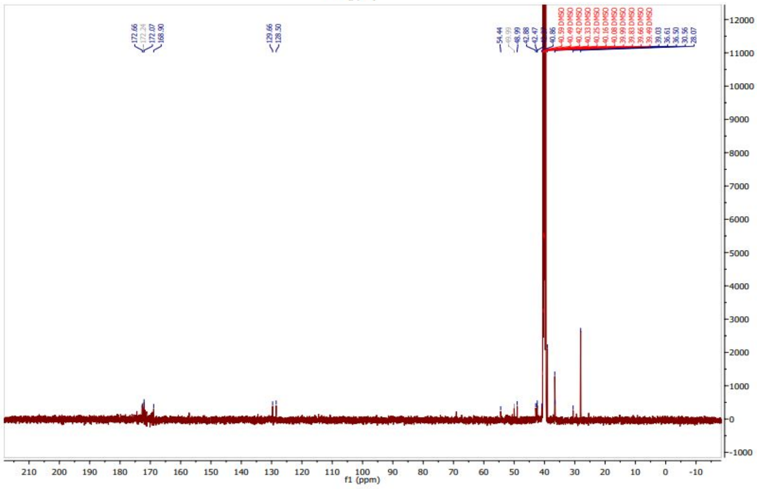

c)

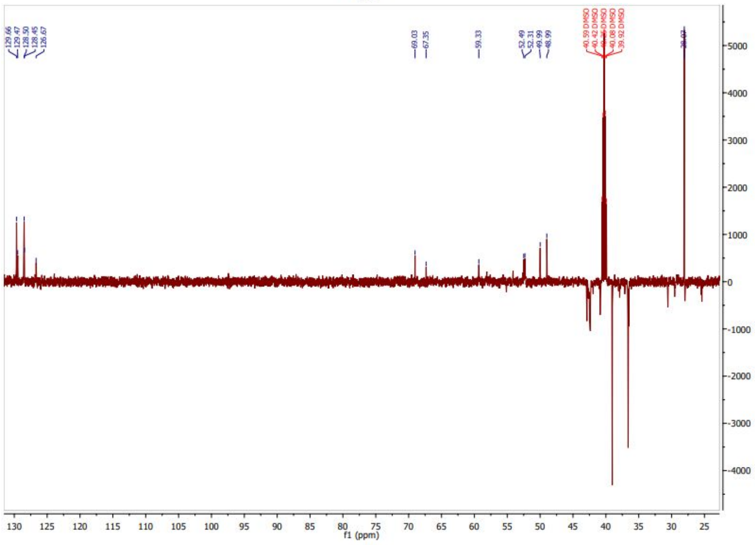

Figure S5 a) ^{1}H NMR *and* **b)** ^{13}C NMR *and c)* DEPT 135 spectrum of DGD-GG-GFOGER-GG-Adamantane(NL009)

a)

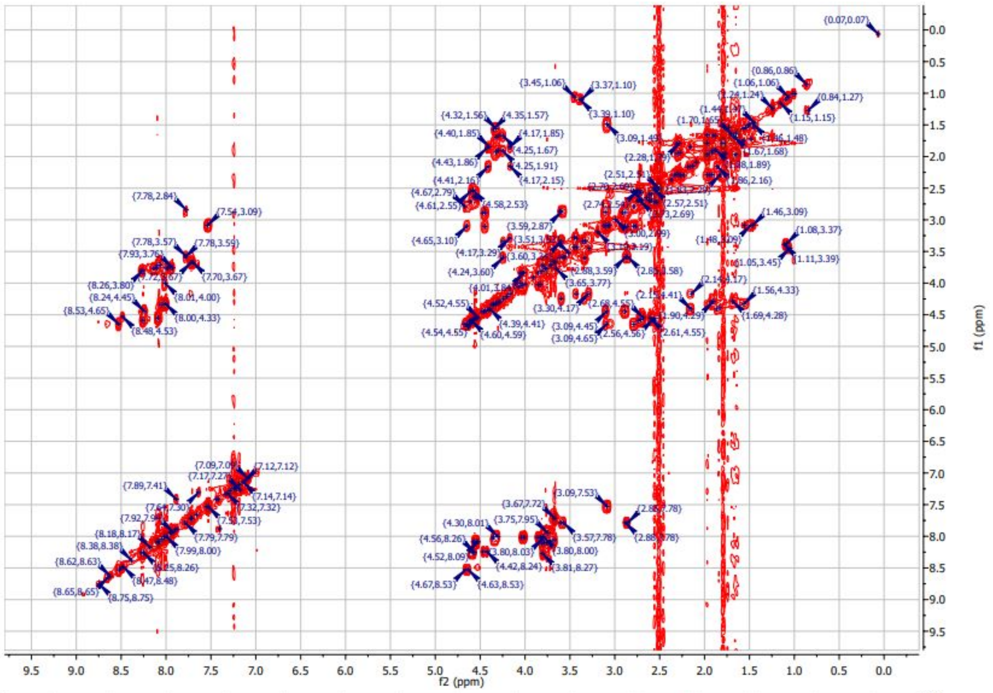

b)

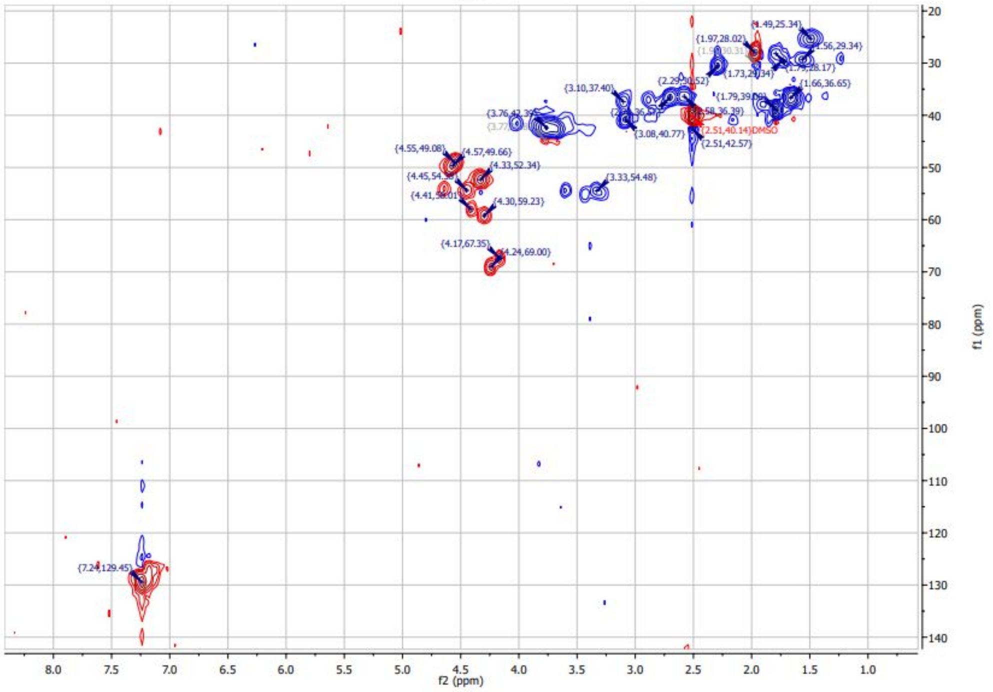

Figure S6 a)COSY and **b)** HSQC spectrum of DGD-GG-GFOGER-GG-Adamantane

a)

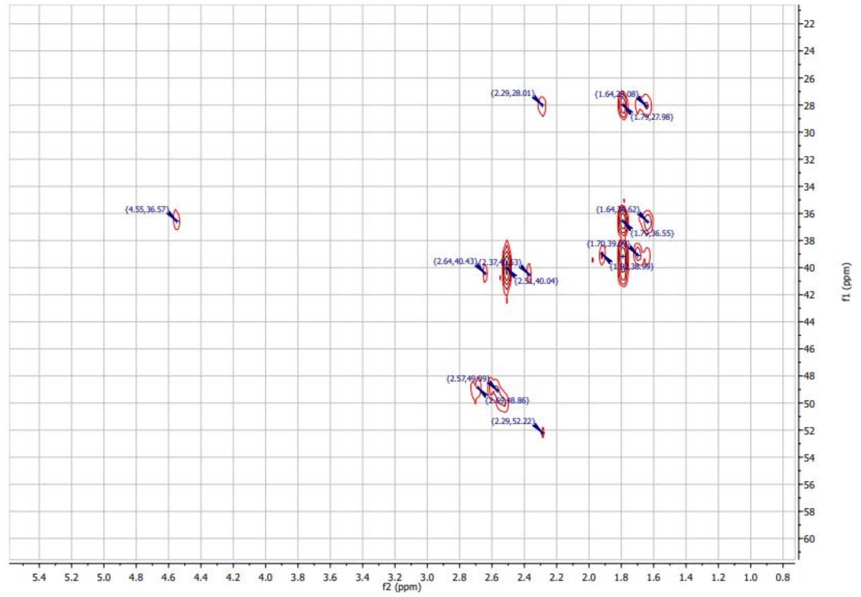

b)

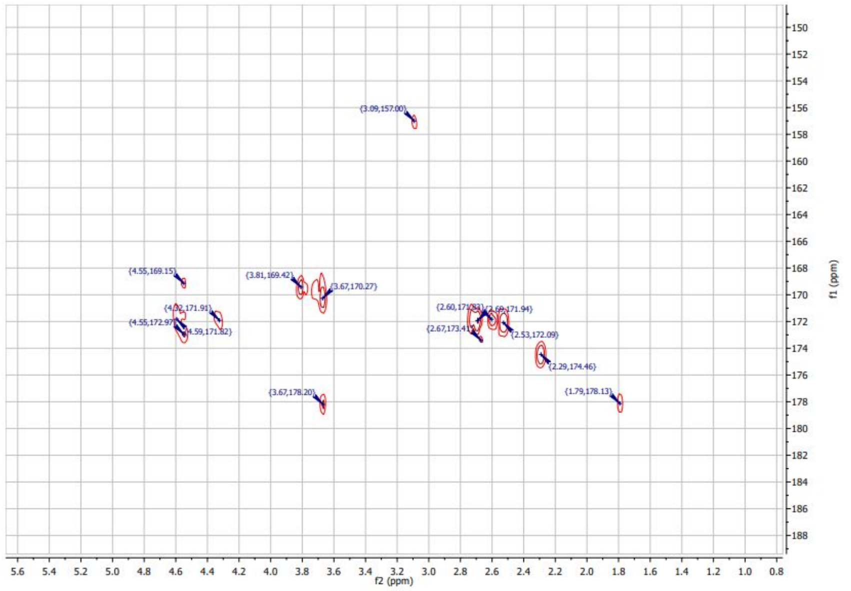

c)

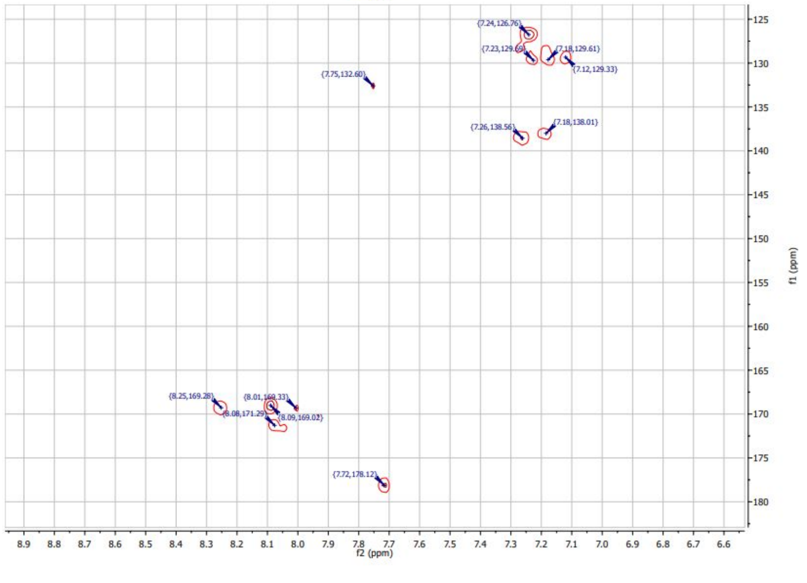

Figure S7 a) HMBC**, b)** Aliphatic, and **c)** Amide-aromatic section of HMBC spectrum of DGD-GG-GFOGER-GG-Adamantane

a)

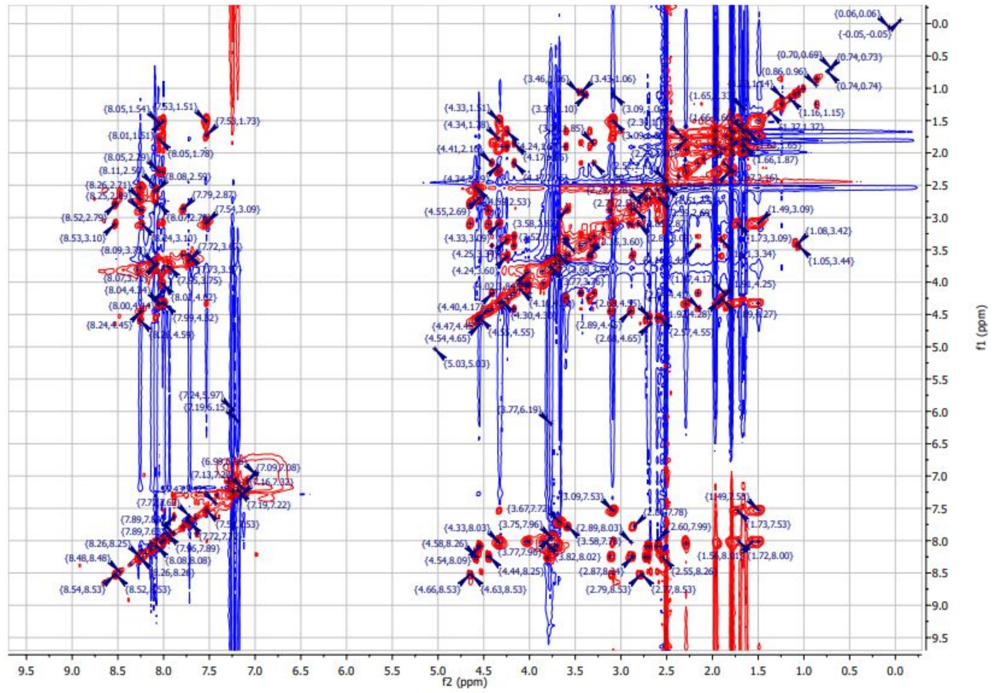

b)

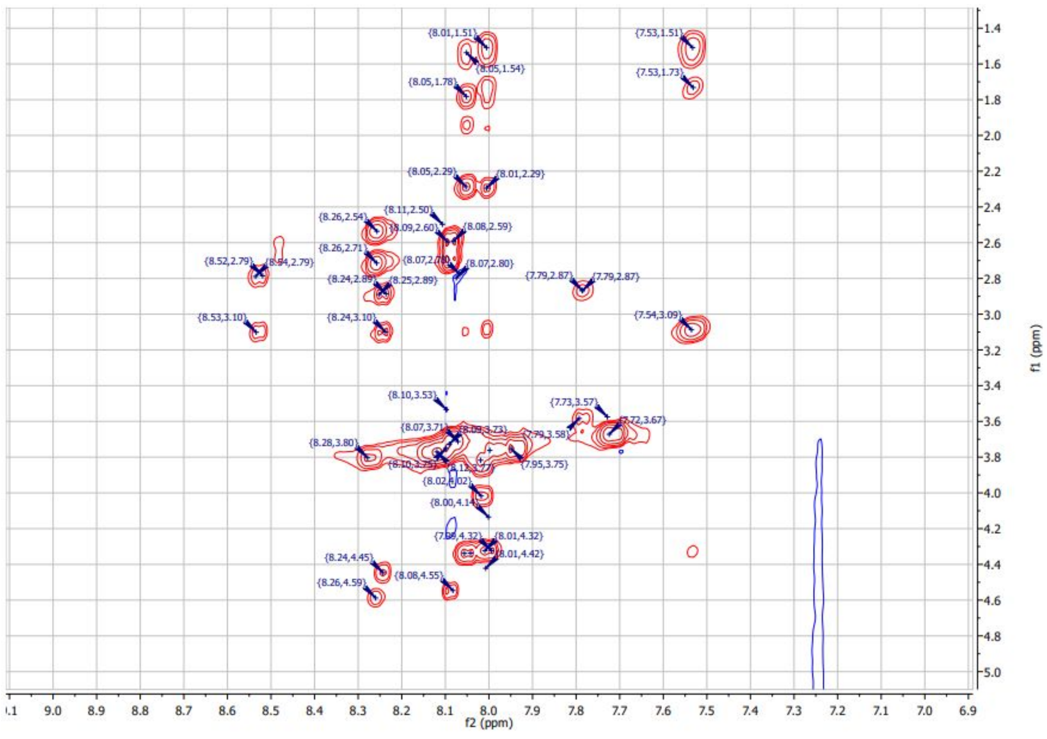

Figure S8 a) TOCSY spectrum and **b)** Amide section of TOCSY spectrum of DGD-GG-GFOGER-GG-Adamantane

a)

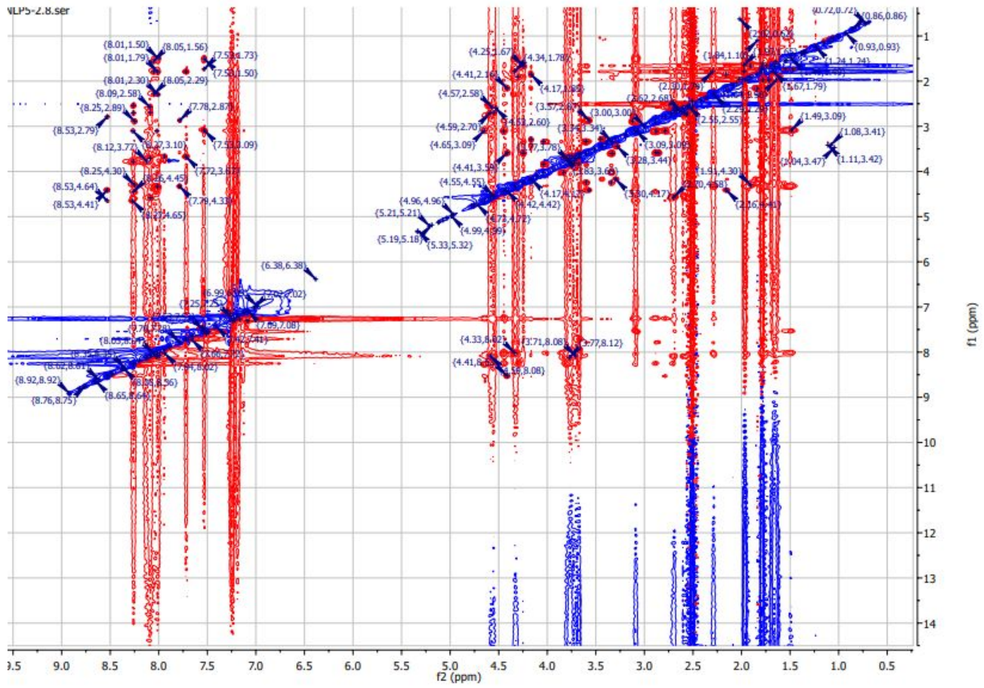

b)

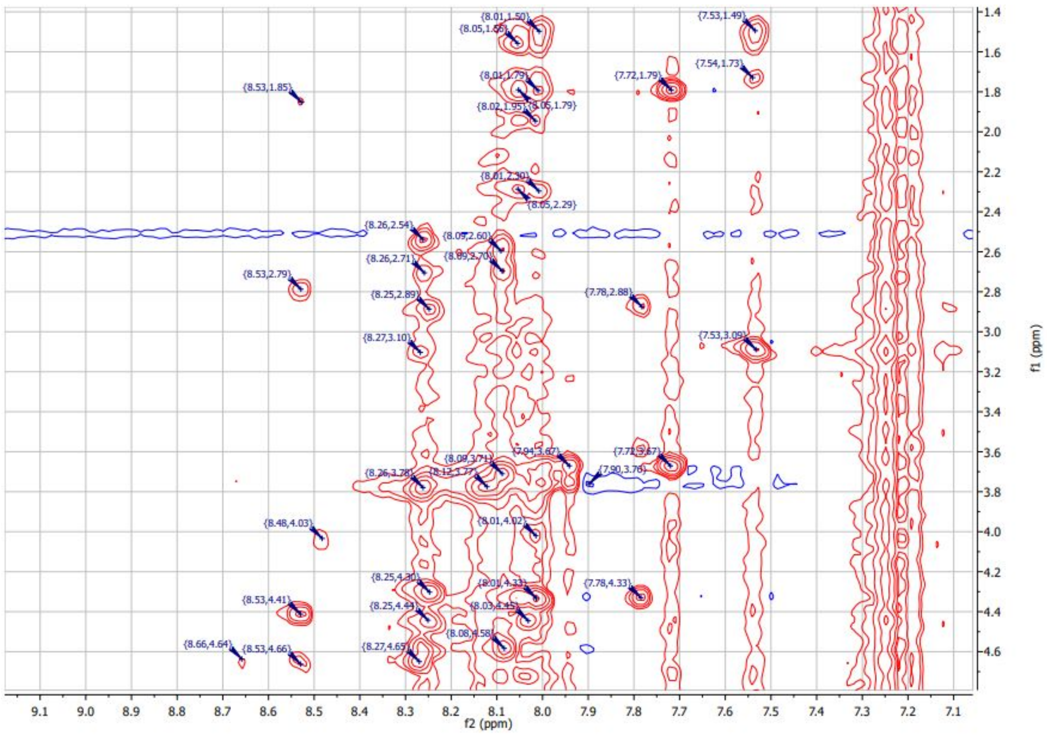

Figure S9 a) ROESY and **b)** Amide section of ROESY spectrum of DGD-GG-GFOGER-GG-Adamantane

a)

b)

c)

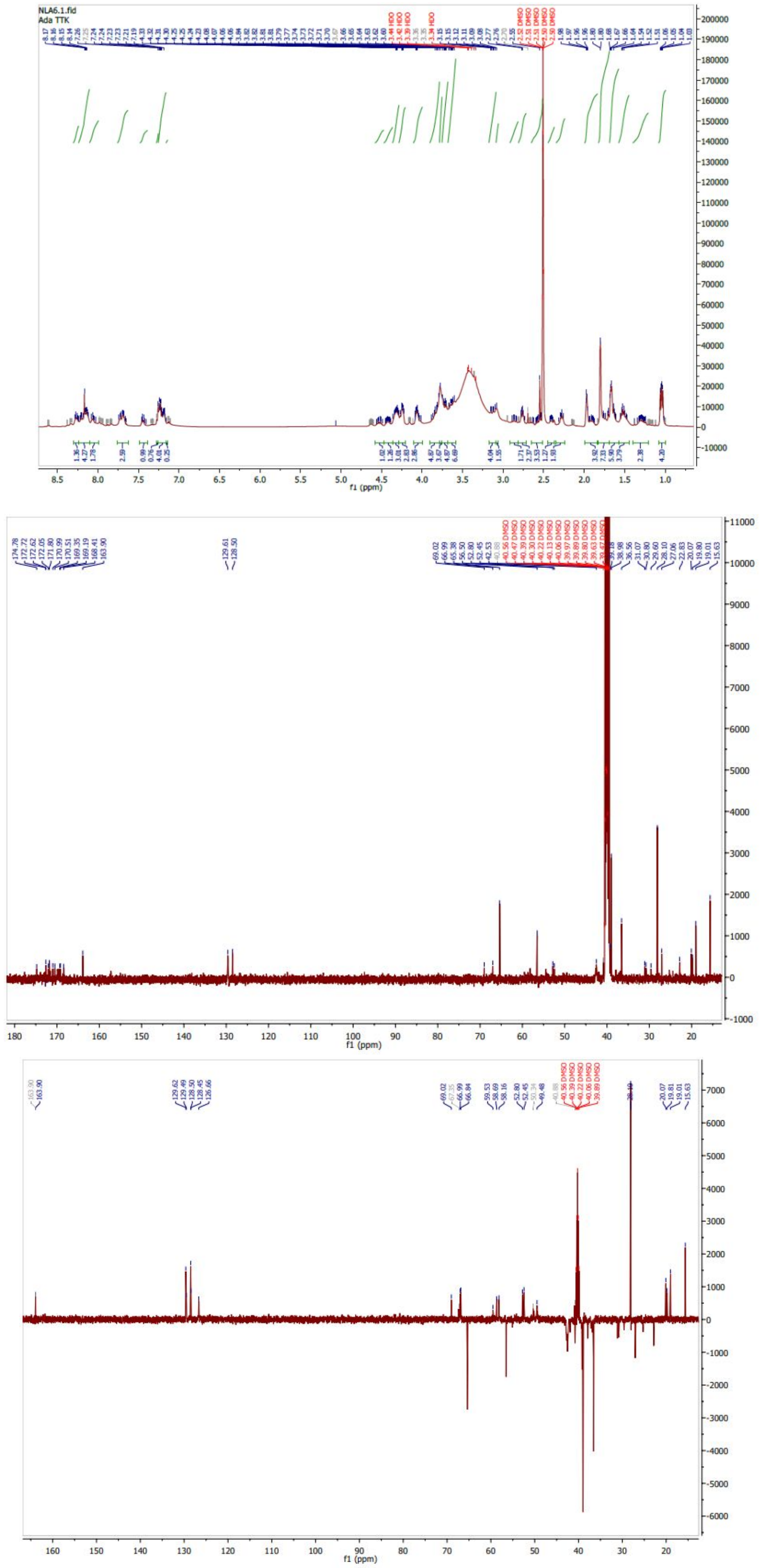

Figure S10 a) ^{1}H NMR **b)** ^{13}C NMR and **c)** DEPT135 NMR spectrum of DGD-GG-GFOGER-GG-TTK-Adamantane(NL010)

a)

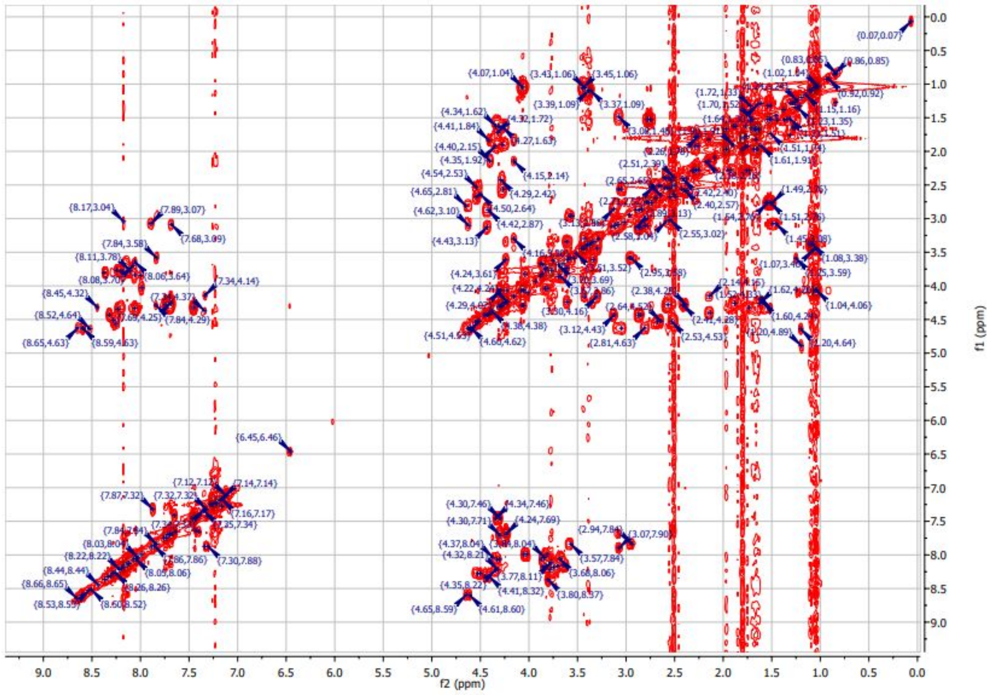

b)

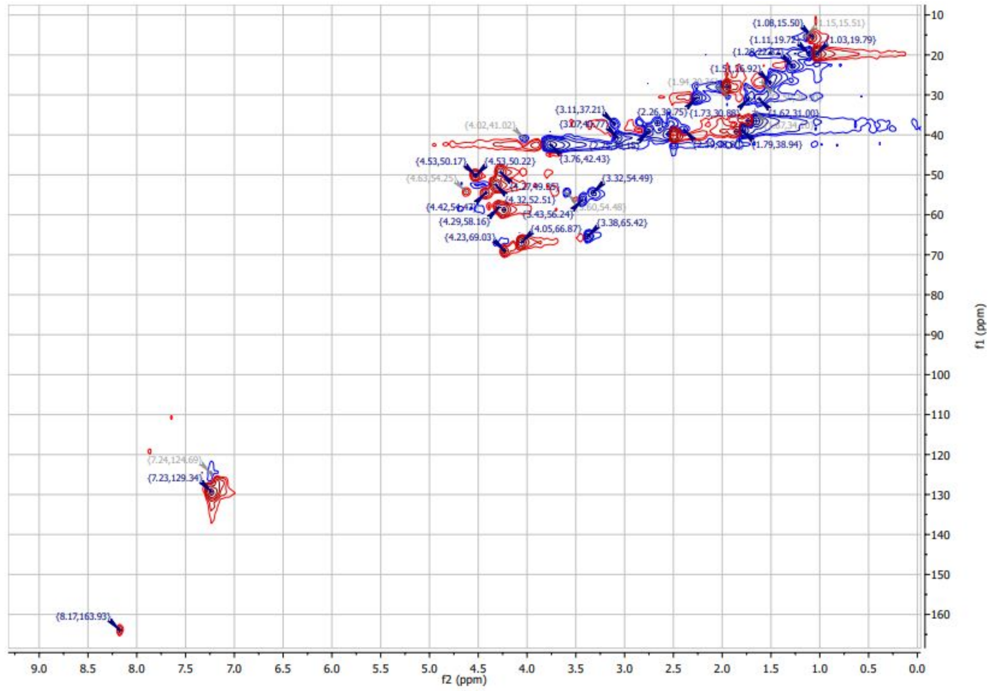

Figure S11 a) COSY and **b)** HSQC spectrum of DGD-GG-GFOGER-GG-TTK-Adamantane

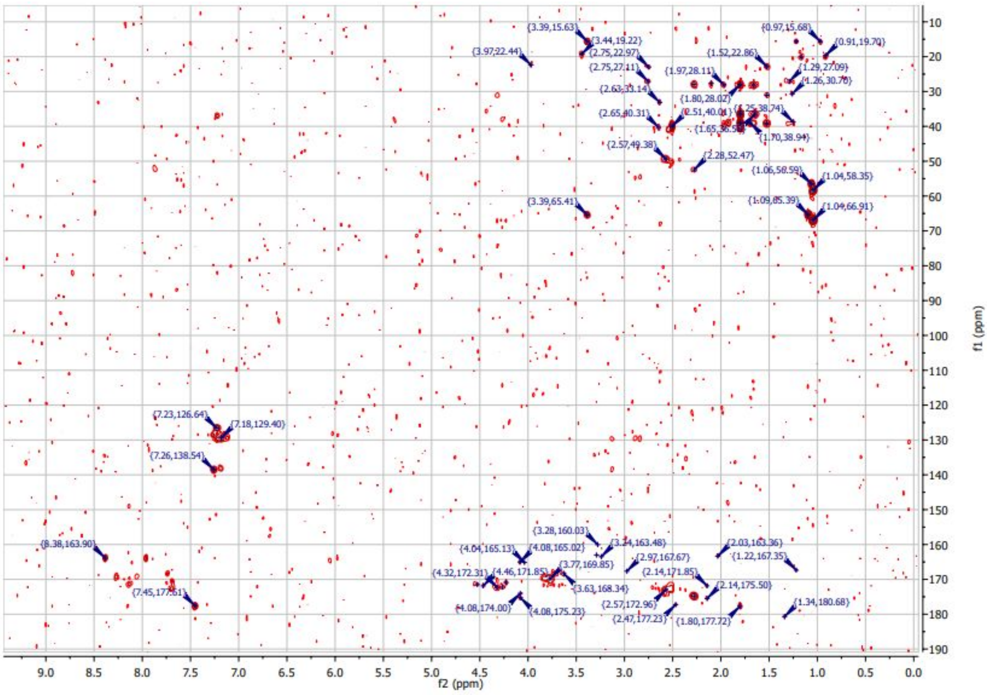

Figure S12 HMBC, spectrum of DGD-GG-GFOGER-GG-TTK-Adamantane

a)

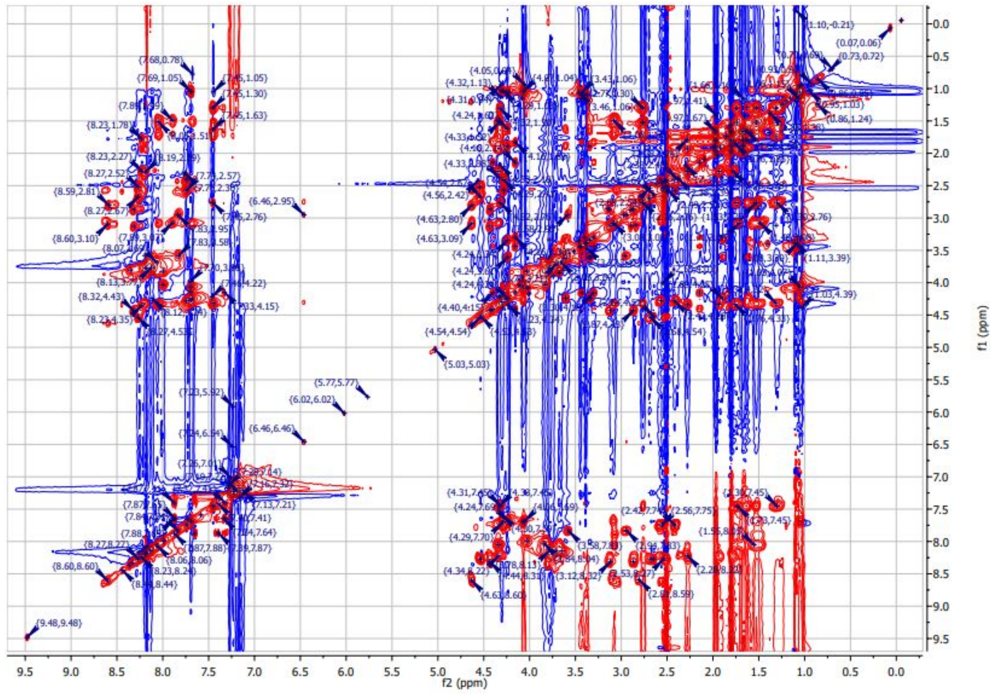

b)

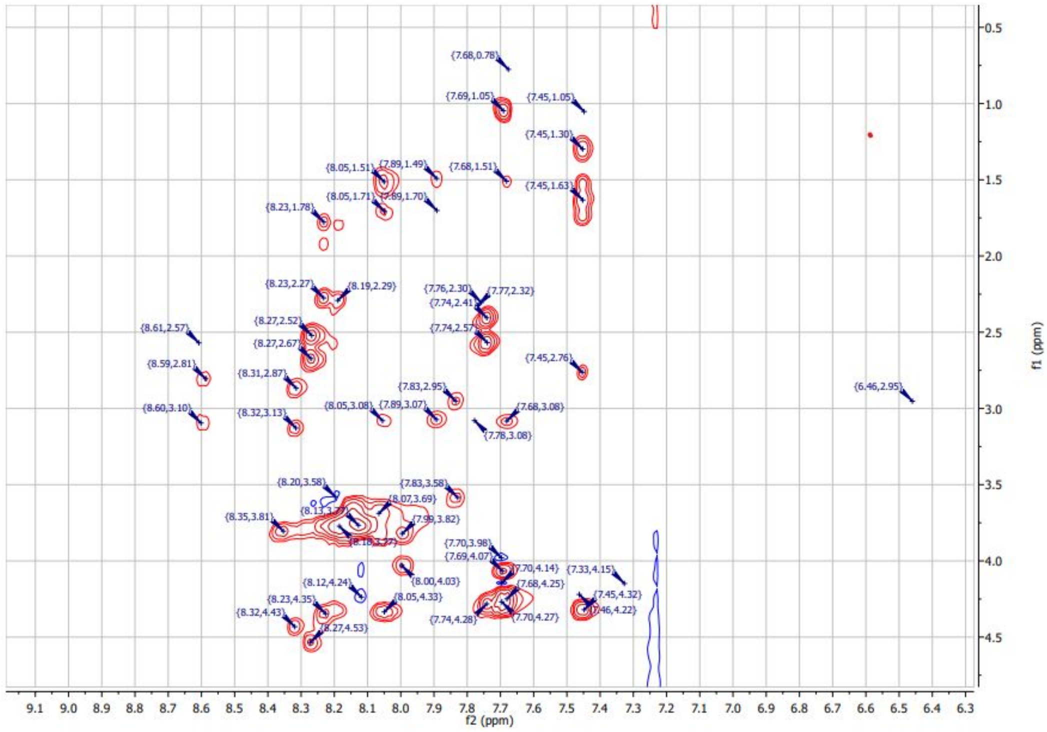

Figure S13 a) TOCSY and **b)** Amide section of TOCSY spectrum of DGD-GG-GFOGER-GG-TTK-Adamantane

a)

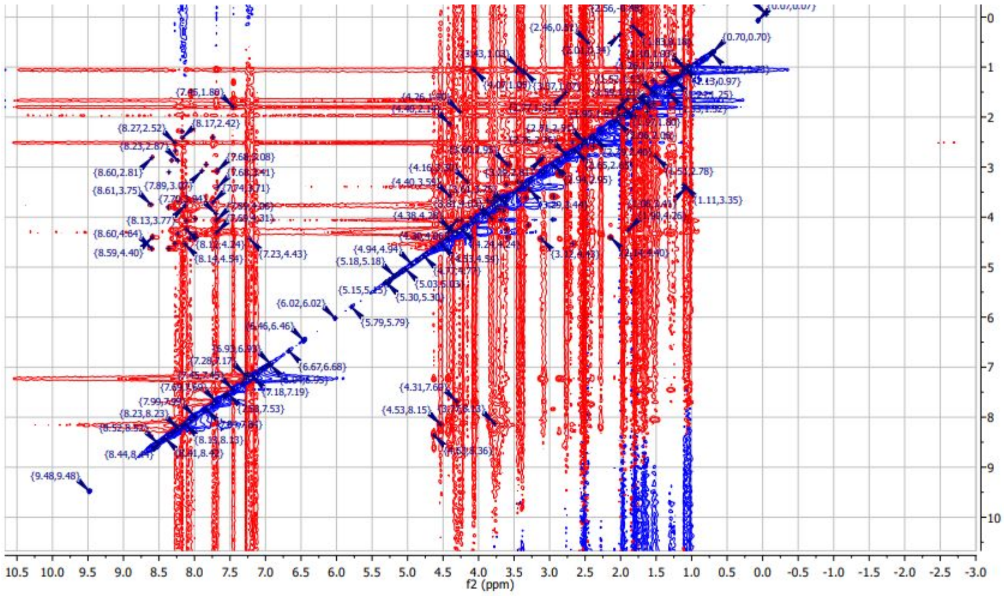

b)

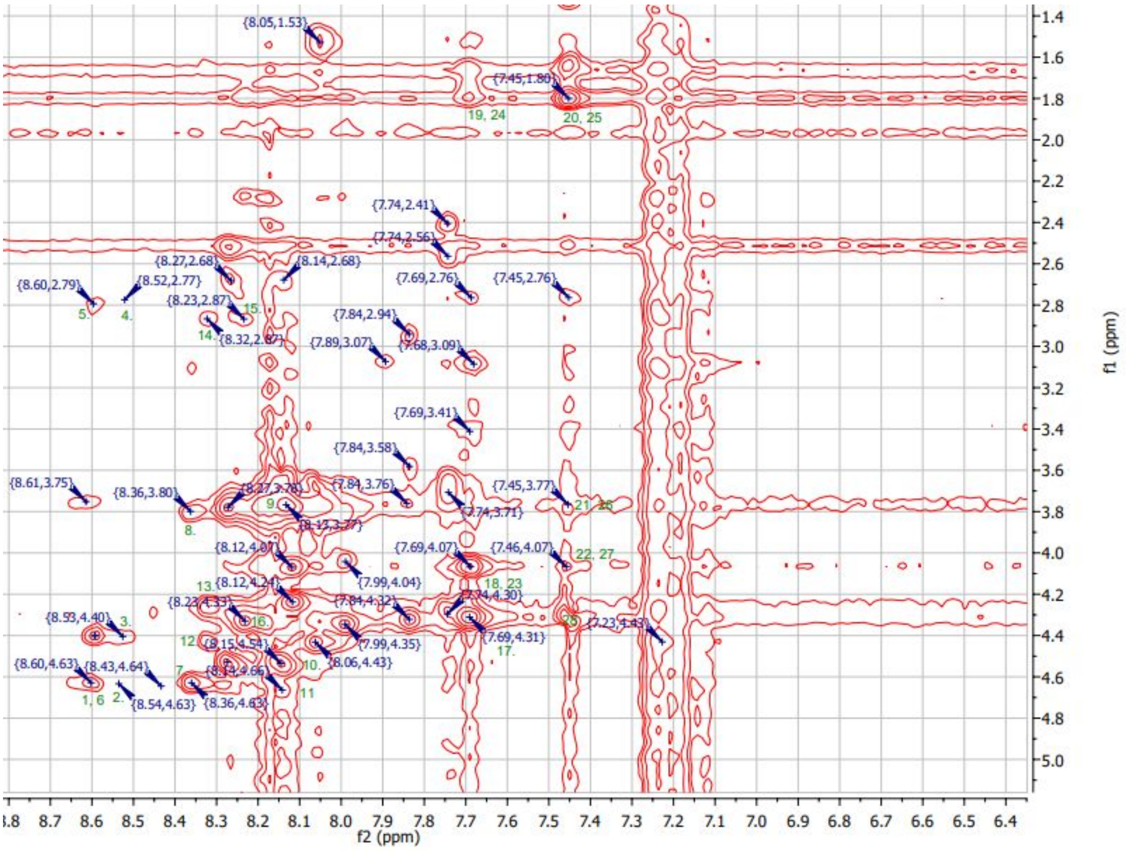

Figure S14 a) ROESY and **b)** Amide section of spectrum of DGD-GG-GFOGER-GG-TTK-Adamantane

CIRCULAR DICHROISM (CD) OF SELECTED PEPTIDES

8.2 DGD-GG-GFOGER-GG-TTK-Adamantane peptide

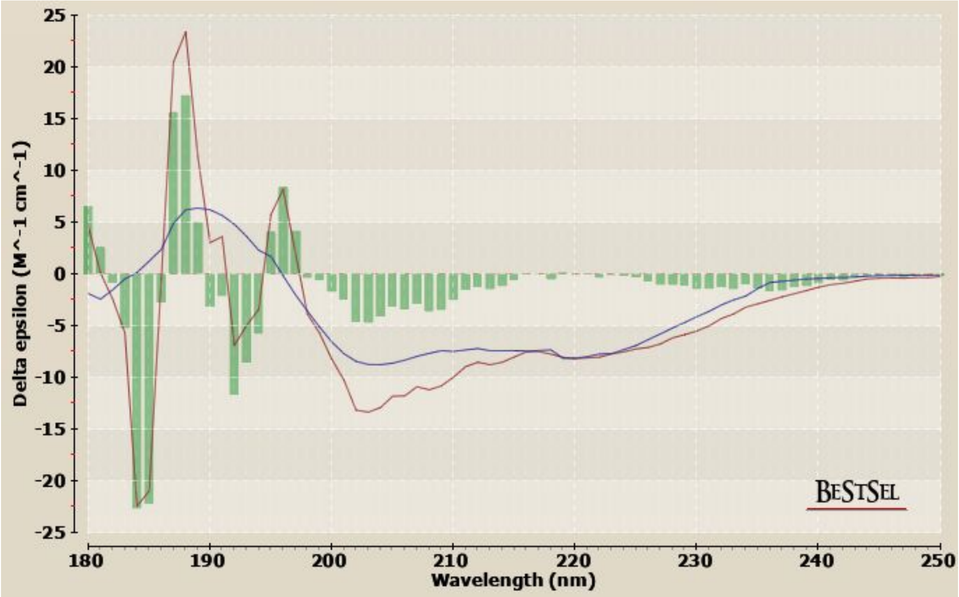

Figure S15. CD spectrum of DGD-GG-GFOGER-GG-TTK-Adamantane peptide (brick red = experimental; blue = fitted)

8. COMPUTATIONAL MODELLING DETAILS

The Lengau Linux cluster hosted by the Centre for High-Performance Computing (CHPC) in South Africa was used to perform all the molecular dynamics (MD) simulations for peptides (NL008, NL009, and NL010). The DESMOND GPU program was used to execute the simulations.

8.1 MOLECULAR DYNAMICS SIMULATIONS

All molecular dynamics (MD) simulations were performed using the Desmond implemented in Schrodinger[1, 2]. The OPLS4 force field[3] was used to model all peptides in the TIP3P solvent model[4]. The long-range electrostatic interactions with a grid spacing of 0.8 were calculated using the particle-mesh Ewald method (PME)[5]. In contrast, the short-range electrostatic interactions and van der Waals were truncated at 9.0 Å. The Nose–Hoover thermostat[6] was used to keep the simulation temperature constant, and the pressure was monitored by Barostat using the Martina–Tobias–Klein method[7]. A RESPA integrator[8] was used with a time step of 2.0 fs for bonded interactions and an outer time step of 6.0 fs for non-bonded interactions beyond the cutoff.

The default settings provided in Desmond were used to equilibrate peptides using a series of restrained minimizations and molecular dynamics simulations designed to relax the system while maintaining the peptides' initial coordinates. The maximum 2000 steps of steepest descent minimization were performed with a harmonic restraint of 50 kcal/mol per Å2 on all solute atoms. Subsequently, four sequential molecular dynamics simulations were performed. The initial simulation was run for 12 ps at a temperature of 10 K in the NVT (constant number of particles, volume, and temperature) ensemble with heavy solute atoms restrained with a force constant of 50 kcal/mol per Å2. Another restraints simulation with the NPT (constant number of particles, pressure, and temperature) ensemble was simulated for 12 ps at 10 K. The next simulation was performed for 24ps with the temperature raised to 325K in the NPT ensemble and the force constant retained. Finally, a 24 ps simulation with all restraints removed was performed at 325 K in the NPT ensemble. The peptides were equilibrated at 5000 ps via the default equilibration NPT simulation. A 200 ns NPT production run was performed at 325 K with configurations saved at 50 ps intervals for each peptide conjugate.

8.2 POST-MD TRAJECTORY ANALYSIS

MD trajectories of the simulated peptides were analyzed using a “Simulation interaction diagram” to assess the simulation parameters over 200 ns MD simulations. The MD production runs were performed in 50ns and later merged to give us a combined 200ns MD trajectory. The root-mean-square deviation (RMSD) and root-mean-square fluctuations (RMSF) were used to measure the dynamic behavior and fluctuations of the amino acid during the 200 ns MD simulations, respectively. All the MD trajectories were clustered based on the peptide amino acid Cα backbone RMSD, especially the snapshots that showed the most defined conformations (α-helices and β-sheet), using the “Desmond Trajectory Clustering utility,” and two clusters were generated, each with a representative structure.

COMPUTATIONAL RESULTS

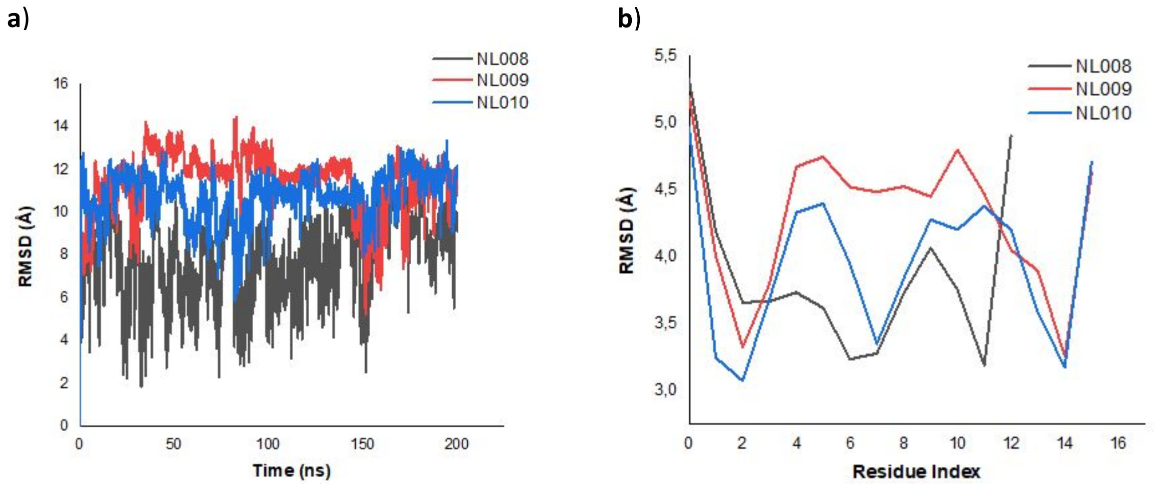

Figure S16. The plots show **a)** the root-mean-square deviations (RMSD) and **b)** root-mean-square fluctuations (RMSF) of the peptides' C-α backbone stability and flexibility after 200 ns MD simulations.

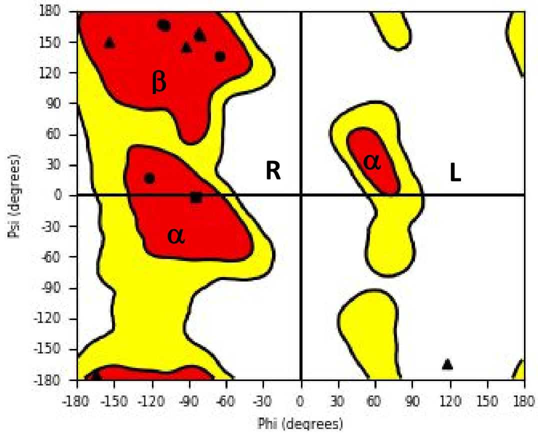

NL008

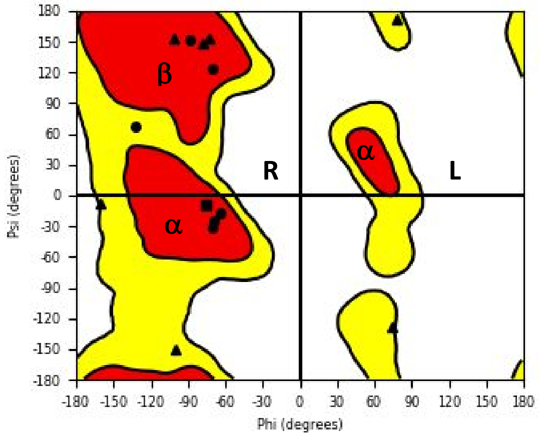

NL009a

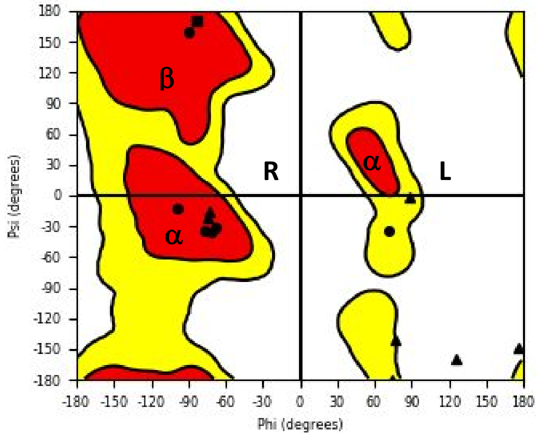

NL009b

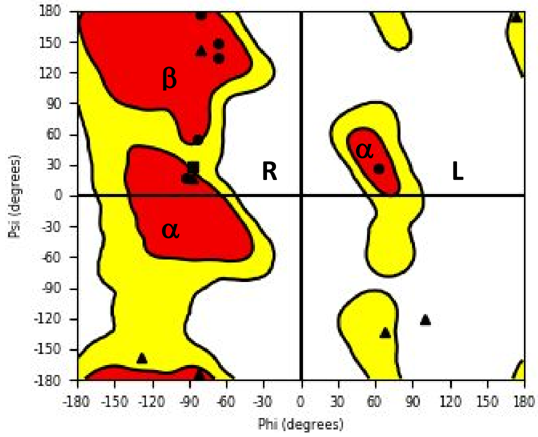

NL010a

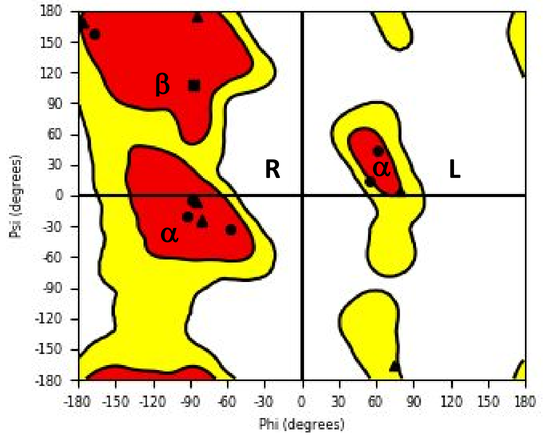

NL010b

Figure S17. Ramachandran plots show the statistical distribution of the psi (***Ψ***) – phi (***ϕ***) dihedral angles of the investigated peptides. The red region represents the favored region, the yellow represents the allowed region, and the white represents the disallowed region. The alpha- helix region is highlighted with α and beta sheet region highlighted with a **β.** **R**–right hand and **L**– left handed.

9. Peptide bioactivity assessed on HaCaT keratinocytes

a)

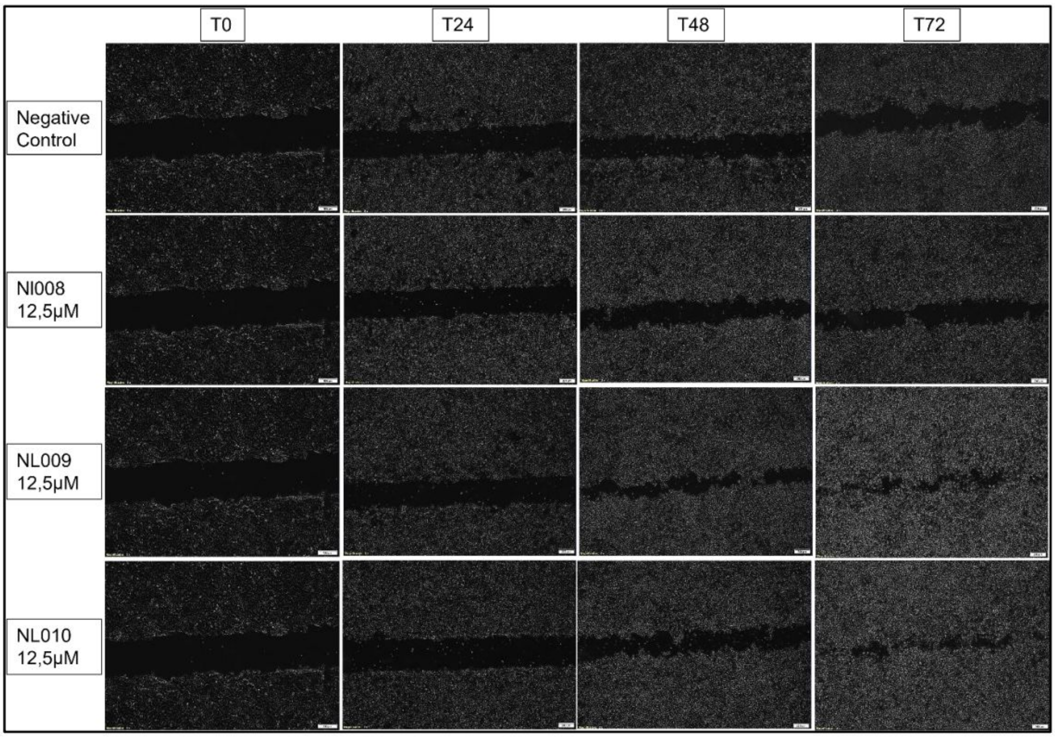

b)

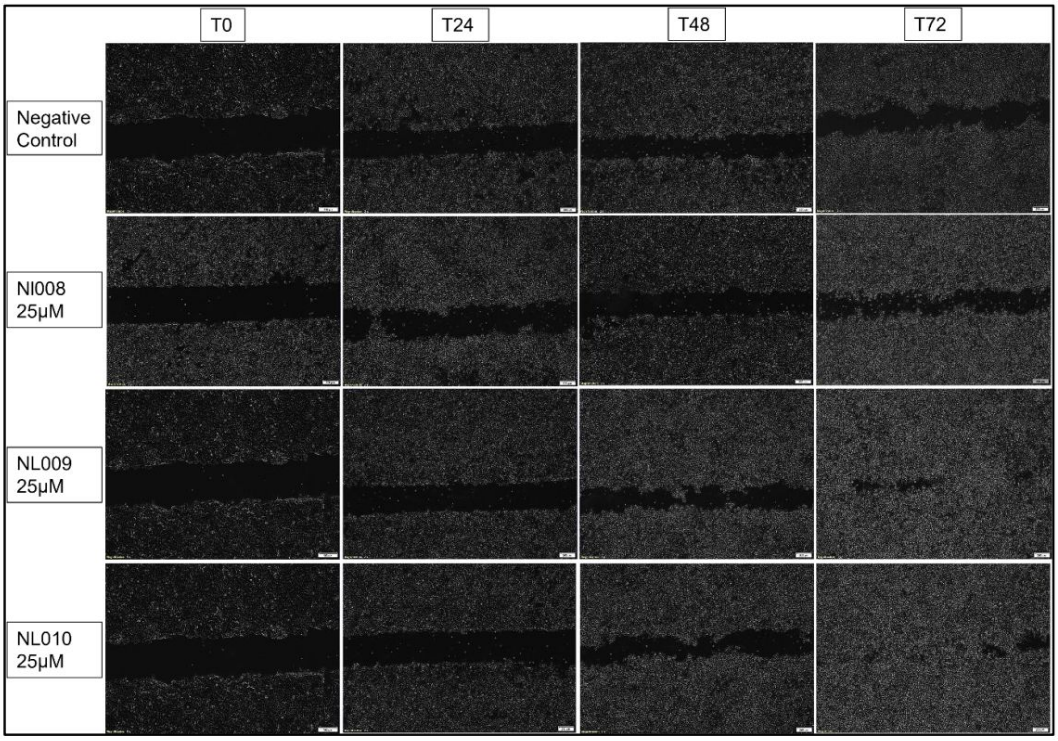

Figure S18. Microscopic scratch closures over time for the three CMPS (NL008, NL009 and NL010) at **a)** 12.5 µM and **b)** 25 µM concentration evaluated on the HaCaT cell line.

10. Peptide bioactivity assessed on 3T3 fibroblasts

a)

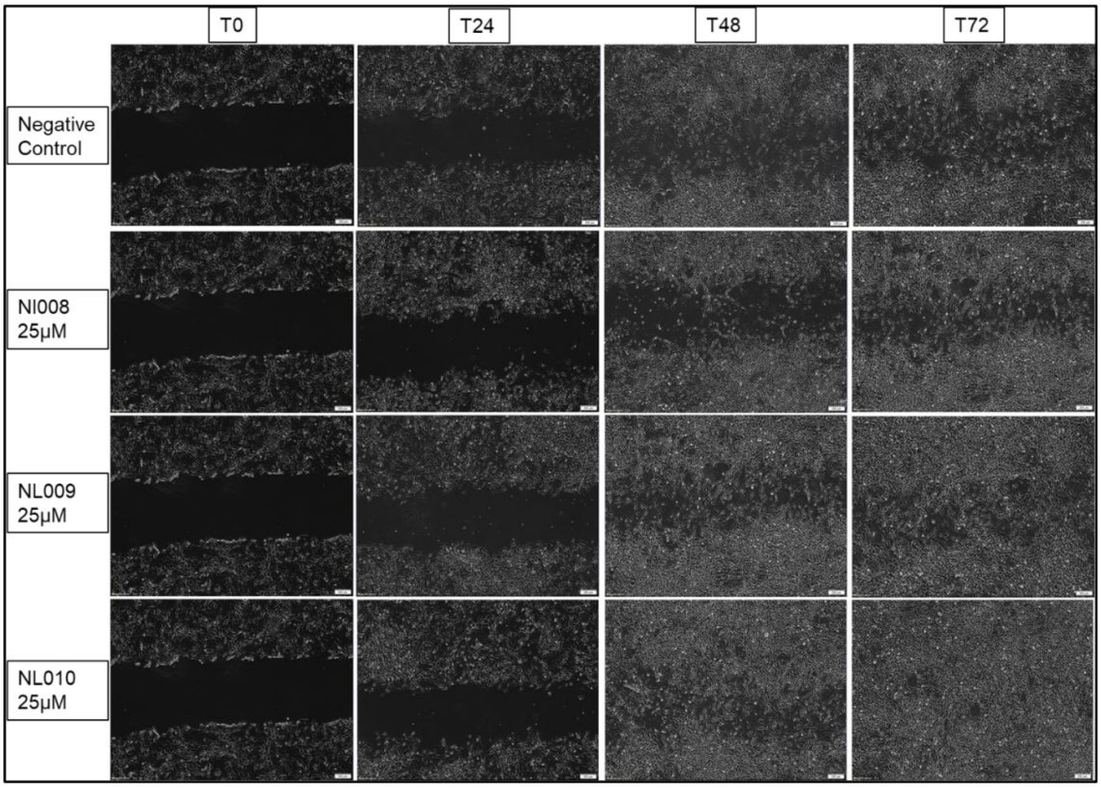

b)

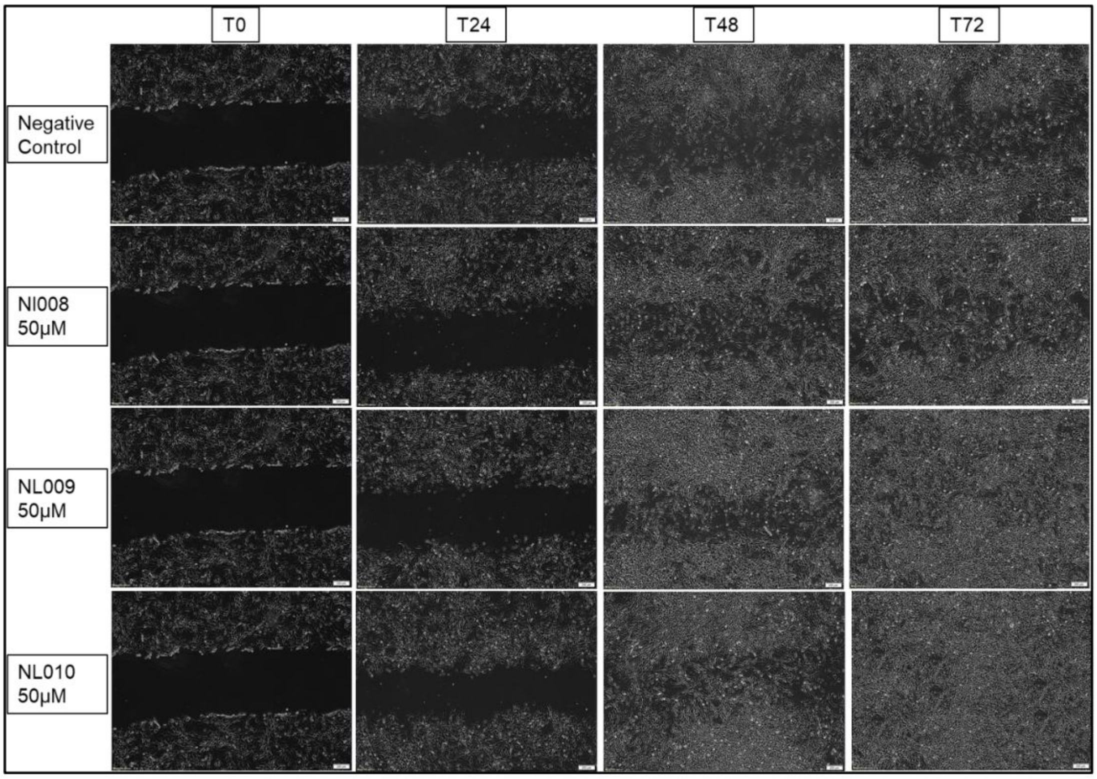

Figure S19. Microscopic scratch closures over time for the three CMPS (NL008, NL009 and NL010) at **a)** 25 µM and **b)** 50 µM concentration evaluated on the 3T3 cell line.

11. Textural analysis

a)

b)

c)

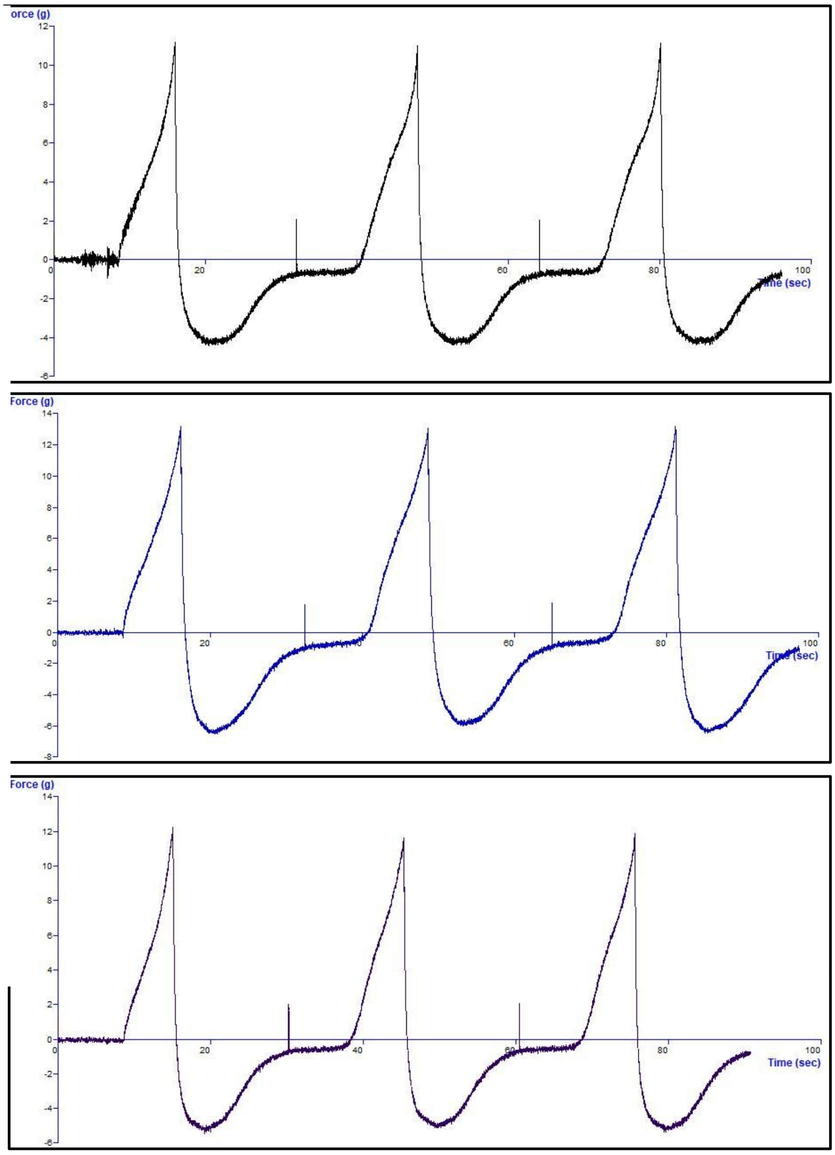

Figure S20. Mechanical profiles of **a)** HAgel; **b)** NL009-HAgel; and **c)** NL010-HAgel represented as Force (N) vs time (sec).

12. Peptide hydrogels bioactivity assessed on HaCaT keratinocytes

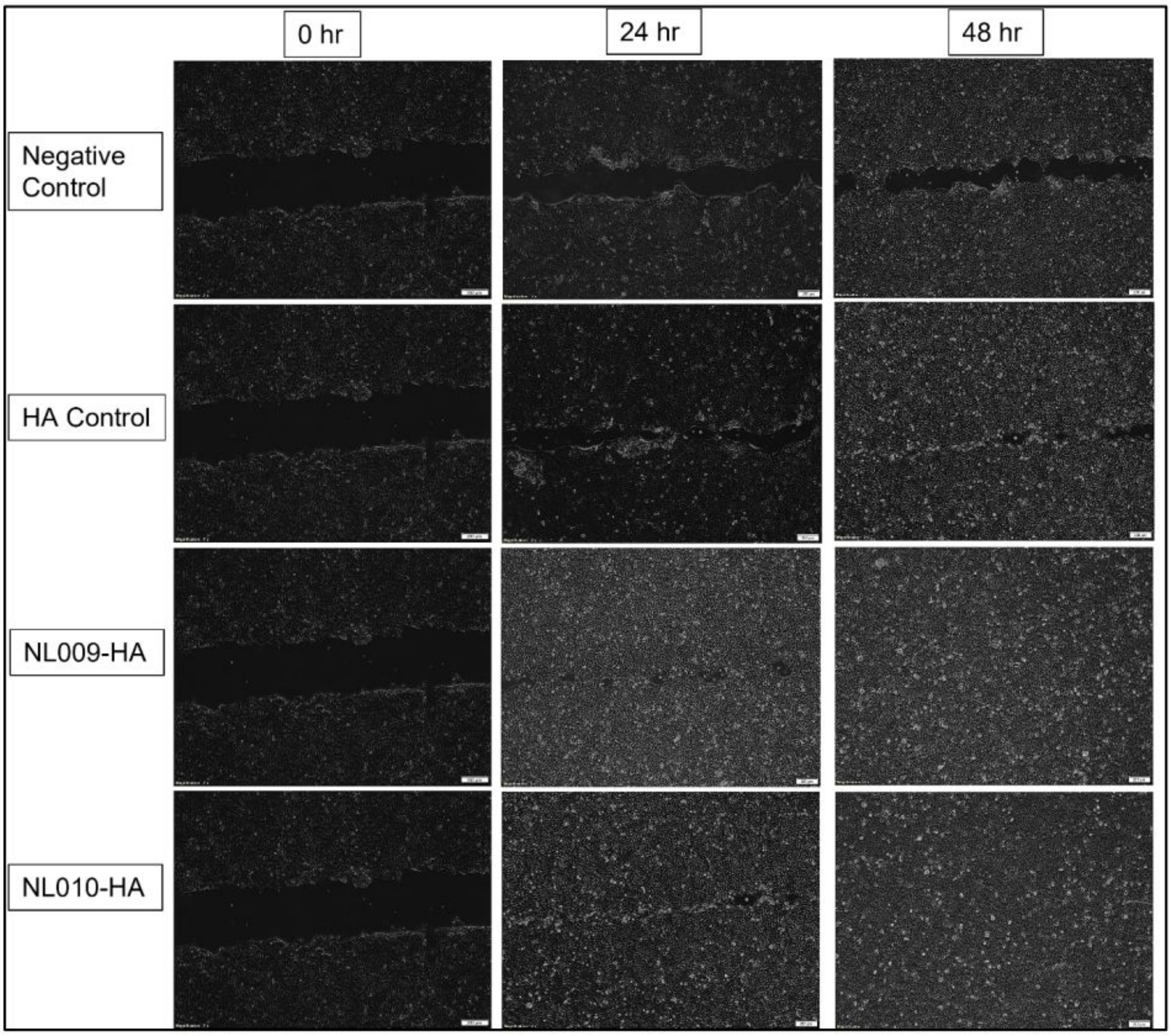

Figure S21. Scratch closure over 48 hours after treatment with HAgel, NL009-HAgel and NL010-HAgel.

References

(1) Bowers, K. J.; Chow, E.; Xu, H.; Dror, R. O.; Eastwood, M. P.; Gregersen, B. A.; Klepeis, J. L.; Kolossvary, I.; Moraes, M. A.; Sacerdoti, F. D. In *Scalable algorithms for molecular dynamics simulations on commodity clusters*, Proceedings of the 2006 ACM/IEEE Conference on Supercomputing, 2006; 2006; pp 84-es.

(2) Bergdorf, M.; Robinson-Mosher, A.; Guo, X.; Law, K.-H.; Shaw, D. E., Desmond/GPU performance as of April 2021. *DE Shaw Research, Tech. Rep. DESRES/TR–2021-01* **2021**.

(3) Lu, C.; Wu, C.; Ghoreishi, D.; Chen, W.; Wang, L.; Damm, W.; Ross, G. A.; Dahlgren, M. K.; Russell, E.; Von Bargen, C. D., OPLS4: Improving force field accuracy on challenging regimes of chemical space. *Journal of chemical theory and computation* **2021,** 17, (7), 4291-4300.

(4) Jorgensen, W. L.; Chandrasekhar, J.; Madura, J. D.; Impey, R. W.; Klein, M. L., Comparison of simple potential functions for simulating liquid water. *The Journal of chemical physics* **1983,** 79, (2), 926-935.

(5) Essmann, U.; Perera, L.; Berkowitz, M. L.; Darden, T.; Lee, H.; Pedersen, L. G., A smooth particle mesh Ewald method. *The Journal of chemical physics* **1995,** 103, (19), 8577-8593.

(6) Hoover, W. G., Canonical dynamics: Equilibrium phase-space distributions. *Physical review A* **1985,** 31, (3), 1695.

(7) Martyna, G. J.; Tobias, D. J.; Klein, M. L., Constant pressure molecular dynamics algorithms. *The Journal of chemical physics* **1994,** 101, (5), 4177-4189.

(8) Humphreys, D. D.; Friesner, R. A.; Berne, B. J., A multiple-time-step molecular dynamics algorithm for macromolecules. *The Journal of Physical Chemistry* **1994,** 98, (27), 6885-6892.
